# Anti-aquaporin 4 IgG Is Not Associated With Any Clinical Disease Characteristics in Neuromyelitis Optica Spectrum Disorder

**DOI:** 10.3389/fneur.2021.635419

**Published:** 2021-03-12

**Authors:** Oliver Schmetzer, Elisa Lakin, Ben Roediger, Ankelien Duchow, Susanna Asseyer, Friedemann Paul, Nadja Siebert

**Affiliations:** ^1^Corporate Member of Freie Universität Berlin, Humboldt-Universität zu Berlin, Charité – Universitätsmedizin Berlin, Berlin, Germany; ^2^Berlin Institute of Health, NeuroCure Clinical Research Center (NCRC) and Experimental and Clinical Research Center (ECRC), Max Delbrück Center for Molecular Medicine (MDC), Berlin, Germany; ^3^Department of Neurology, Charité – Universitätsmedizin Berlin, Berlin, Germany; ^4^Novartis Institutes for Biomedical Research - Autoimmunity, Transplantation and Inflammation, Basel, Switzerland

**Keywords:** immunology, autoimmunity, neuromyelitis optica, aquaporin, channelopathies, neuromyelitis optica, AQP4, anti-AQP4-IgG

## Abstract

**Background:** Neuromyelitis optica spectrum disorder (NMOSD) is a clinically defined, inflammatory central nervous system (CNS) disease of unknown cause, associated with humoral autoimmune findings such as anti-aquaporin 4 (AQP4)-IgG. Recent clinical trials showed a benefit of anti-B cell and anti-complement-antibodies in NMOSD, suggesting relevance of anti-AQP4-IgG in disease pathogenesis.

**Objective:** AQP4-IgG in NMOSD is clearly defined, yet up to 40% of the patients are negative for AQP4-IgG. This may indicate that AQP4-IgG is not disease-driving in NMOSD or defines a distinct patient endotype.

**Methods:** We established a biobank of 63 clinically well-characterized NMOSD patients with an extensive annotation of 351 symptoms, patient characteristics, laboratory results and clinical scores. We used phylogenetic clustering, heatmaps, principal component and longitudinal causal interference analyses to test for the relevance of anti-AQP4-IgG.

**Results:** Anti-AQP4-IgG was undetectable in 29 (46%) of the 63 NMOSD patients. Within anti-AQP4-IgG-positive patients, anti-AQP4-IgG titers did not correlate with clinical disease activity. Comparing anti-AQP4-IgG-positive vs. -negative patients did not delineate any clinically defined subgroup. However, anti-AQP4-IgG positive patients had a significantly (*p* = 0.022) higher rate of additional autoimmune diagnoses.

**Conclusion:** Our results challenge the assumption that anti-AQP4-IgG alone plays a disease-driving role in NMOSD. Anti-AQP4-IgG might represent an epiphenomenon associated with NMOSD, may represent one of several immune mechanisms that collectively contribute to the pathogenesis of this disease or indeed, anti-AQP4-IgG might be the relevant factor in only a subgroup of patients.

**Key Messages:**

- No clinical differences between anti-AQP4-IgG positive or negative patients
- No significant change of anti-AQP4-IgG levels during disease progression, but a non- significant increase in the mean anti-AQP4-IgG titer was visible only after multiple relapses
- anti-AQP4-IgG^+^ patients have a significantly (p = 0.022) higher rate of additional autoimmune diagnoses.

## Introduction

Neuromyelitis optica spectrum disorder (NMOSD) is a rare [prevalence about 1:100,000; incidence 0.1-0.4 per 100,000 (1–4)], devastating chronic inflammatory CNS disease. There is no clear cause for the disease, and NMOSD in the past had a grim prognosis in many cases due to limited therapeutic options (1, 5–7). NMOSD is clinically characterized by attacks of uni- or bilateral optic neuritis (ON), acute myelitis and/or brain/brainstem encephalitis (8) and is associated with specific autoantibodies (aAbs) such as anti-aquaporin-4 (AQP4), targeting an astrocytic water channel (9), or anti-myelin oligodendrocyte glycoprotein (MOG)-IgG (5, 10). Approximately 60% of all NMOSD patients are positive for anti-AQP4-IgG (11–13). Anti-AQP4-IgG is therefore thought to play a key or even causal role in disease pathogenesis, characterizing the anti-AQP4-IgG positive subset of NMOSD as a channelopathy (11).

There are several other lines of evidence suggesting that NMOSD is a humoral autoimmune disease: (1) Detection of substantial local deposition of vasculocentric, activated complement in active lesions in patients (14); (2) Recapitulation of NMOSD-like clinical features in rodents following passive transfer of patient serum (15, 16); (3) Capacity of NMO-IgG to bind to the extracellular domain of AQP4 on astrocytes and activate complement *in vitro* (17, 18); and (4) Quantitative measures of complement-mediated injury to AQP4-expressing cells *in vitro* could be correlated with clinical disease progression in a limited number of patients (19). Indeed, a recent clinical trial with eculizumab, a complement factor 5 (C5) antibody, demonstrated clinical efficacy in anti-AQP4-IgG-positive patients, supporting the notion that NMOSD is a complement-dependent disorder of the CNS (20, 21). Furthermore, complement-independent AQP4-antibody-mediated astrocytopathies have been proposed from *in vitro* cell culture models (22) and B cell targeting therapies such as anti-CD20 and anti-CD19 have been shown to prevent relapses and are now used as first line therapeutic options for this disease (23–25).

Despite this evidence, more recent trials have failed to show a correlation of complement-mediated cell killing activity with relapse rates or relapse severity (26) and questioned the role of anti-AQP4-IgG (27). In addition, in most rodent *in vivo* models, neither induction of anti-AQP4-IgG by immunization, its presence by transgenic anti-AQP4 expression nor continuous infusion of anti-AQP4-IgG was sufficient to cause disease (16). In these preclinical models, NMOSD-like pathology could only be recapitulated if human (not murine) complement was co-administered intracerebrally.

The question of the role and relevance of anti-AQP4-IgG is of high therapeutic relevance, as only approximately between half and two-thirds of the patients are positive for these antibodies, and eculizumab has only been trialed in AQP4-positive patients. Although NMOSD patients are clinically well-defined and relatively homogeneous, and robust endotypes have not been identified to date, the question of whether there exist clinically relevant, AQP4-positive and AQP4-negative subtypes has not been formally addressed.

Here, we investigated 63 clinically well-characterized NMOSD patients to better understand the pathogenesis of this debilitating disease and assess the relevance of anti-AQP4-IgG positivity. Our results challenge the currently assumed, disease-driving role for anti-AQP4-IgG, which holds therapeutic relevance for NMOSD sufferers. Our findings also expand our present understanding of antibody driven autoimmunity, complement and neurodegeneration. However, we finally cannot prove nor rule out that anti-AQP4-IgG might indeed play a major disease-driving role in a subset of patients.

## Materials and Methods

### NMOSD Patients

Demographic characteristics, diagnoses, medical history, age at disease onset, time to diagnosis, duration of clinical observation, clinical attacks, attack-related factors such as clinical presentation, attack treatment, attack outcome and remission rate, resulting disability, other therapies, short-term remission status, laboratory values from routine tests, AQP4- and MOG-aAb status as determined by cell-based assays [due to its high relevance in this disease group (28–30)], annualized relapse rate (ARR), optical coherence tomography (OCT) (24) as well as magnetic resonance imaging (MRI) results (31) and an array of other variables (Supplementary Table 1) have been recorded from over 100 adult NMOSD patients from an ongoing prospective longitudinal observational study conducted at the NeuroCure Clinical Research Center of Charité Universitaetsmedizin Berlin according to standardized Wingerchuk 2015 criteria (32). In addition, an extensive test battery including Expanded Disability Status Scale (EDSS), Multiple Sclerosis Functional Composite (MSFC), The Short Form (36) Health Survey (SF-36), fatigue severity scale (FSS), Fatigue Scale for Motor and Cognitive Functions (FSMC), Beck Depression Inventory (BDI), McGill Pain Questionnaire (MPQ), painDETECT questionnaire (PDQ), Brief Pain Inventory (BPI), National Eye Institute Visual Function Questionnaire (NEI-VFQ), National Eye Institute Visual Function Questionnaire (NEI-VFQ), Neuro-Ophthalmic Supplement (NOS) and visual analog scales (VAS) for general well-being, cognition, pain and fatigue, were also included. A complete list of all 1,232 variables can be found in Supplementary Table 1. We could include 351 of these variables in our analyses, all of which resulted in a numeric, non-descriptive value or which could be turned into a numeric result, and for which we had a reasonable amount of tested subjects.

Inclusion criterion for patients was a diagnosis of NMOSD according to Wingerchuk et al.'s 2015 criteria (32). The data collection started in 2008 in order to merge and facilitate clinical and research activities around NMOSD. Sixty-three patients had full datasets and were included in further analyses, demographic data and aAb status can be found in Supplementary Table 2. These patients (79% female, mean age at diagnosis 45 years) had a mean duration of disease of 4.1 years (median 5.5 years) with a mean of 2.73 relapses (median two relapses) and a mean disability of 7.42 points in the EDSS (median nine points) after enrollment.

All data was stored, archived and disposed in a safe and secure manner during and after the conclusion of the research project in line with current data protection regulations (General Data Protection Regulation: DSGVO). We have established policies and procedures to manage data handled electronically as well as through non-electronic means in accordance with good laboratory practice.

### Ethics Statement

All data collected from patients and investigated in this study was used after written informed consent as approved by the ethics committee of the Charité–Universitätsmedizin Berlin (proposal EA1/041/14) according to the Declaration of Helsinki (59th WMA General Assembly, Seoul, October 2008).

### Statistical Analyses

Principal Component Analyses (PCA) and heatmap visualization were performed using ‘ClustVis’ (33) as we did it before (34). In brief, SVD (Singular Value Decomposition) with imputation (as the most common method) or Nipals PCA model was used to calculate the principal components (PC). PC 1 and 2 were presented on the X and Y axis while the percentages of represented total variances were shown in brackets. Different methods (Pareto, vector, or mostly Unit variance) were used as scaling method applied to the rows or no scaling was used. Imputation and SVD (or Nipals PCA) were iteratively performed until estimates of missing values converged. We drew ellipses for each dataset, datapoints fall with a probability of 0.95 inside these ellipses.

In an attempt not to miss a fitting model due to artificial fragmentation of patients' populations due to overanalyses, we used a stepwise approach based on our initially collected 1,232 variables. The most extensive set consisted of all 351 numeric variables (listed in Supplementary Figure 1 on the Y-axis). We also tested a medium sized set of 47 variables which was left after the extensive clinical score details and deeply investigating single symptom areas had been removed, leaving only the score results in the dataset. Two further reduced sets of a limited number of 20 and 25 key variables were also analyzed, after variables dealing with the relapses had been removed. This was done because we had only data on relapses for a fraction of all patients. Finally, a core set of 18 variables was analyzed after additional other diagnoses (mainly further autoimmune diseases) of the patients were removed.

Cladograms and heatmaps were generated after phylogenetic cluster analyses were performed. Patients were clustered in columns (shown on the X-axis) and variables were clustered in rows (presented on the Y-axis) which were centered. Different (correlation, binary, Canberra, Manhattan, maximum, euclidean and most often correlation) distance and different (*Ward as unsquared distances, simple Ward, McQuitty, complete, single and* most often average) linkage methods were used as models, as stated in the legends. Unit variance scaling was in some analyses applied only to the variables in the rows. On top of the rows, anti-AQP4 status or as an example the SF-36 variable: “limitations due to pain” are shown in red and blue. Imputation was used for missing value estimation. The results were expressed as Z-scores.

Additional autoimmune diseases correlated with anti-AQP4-IgG-positivity and we used chord plotting with “Circos” (35) to demonstrate this.

Normal distribution was tested with the Anderson-Darling, the D'Agostino & Pearson, the Shapiro-Wilk and the Kolmogorov-Smirnov tests. An approximately Gaussian distribution served as basis to perform Student's t and ANOVA tests using GraphPad Prism version 8.4.3 for Windows, GraphPad Software, La Jolla California USA, www.graphpad.com. Most data were not normally distributed, we therefore used a non-parametric, unpaired and two-tailed Mann–Whitney test. The null hypothesis of random results was rejected if the *P*-value was very small <0.05.

Exon expression was used from GTEx (Genotype-Tissue Expression) Analysis Release V8 (dbGaP, database of Genotypes and Phenotypes, Accession phs000424.v8.p2) based on expression data of AQP4: ENSG00000171885.13 from HGNC (Human Genome Organization, HUGO Gene Nomenclature Committee; HGNC Accession: 637; https://www.gtexportal.org/home/gene/AQP4).

## Results

### Anti-AQP4-IgG Status Does Not Define a Subgroup

In order to better understand the spectrum of clinical and pathological features of NMOSD, we established a biobank of 63 clinically well-characterized NMOSD patients regularly seen at our institution (Supplementary Tables 1, 2) as part of an ongoing observational study with an extensive annotation of 351 singular symptoms, patient characteristics (such as biographic, demographic, and social data), laboratory results, clinical scores, imaging (MRI and OCT) findings and physical examination results. Of these 63 patients, 34 were anti-AQP4-IgG^+^ and 29 were anti-AQP4-IgG^−^. anti-MOG-IgG could be identified in 18 patients, one patient was double positive for anti-AQP4-IgG and anti-MOG-IgG. From this, we wanted to find a mathematical model, which fits to the anti-AQP4-IgG-status and correlates with the clinical variables. Therefore, we used numerous mathematical models to test whether aAbs such as anti-AQP4-IgG could play a role in the pathophysiology and the clinical characteristics of NMOSD. We repeated those analyses for different collections of variables as well as for the full set. This was performed as it was not clear, how many and which variables would be congruent and related to the anti-AQP4-IgG-status. Specifically, we applied phylogenetic clustering, PCA and longitudinal causal interference analysis.

**Figure 1:**
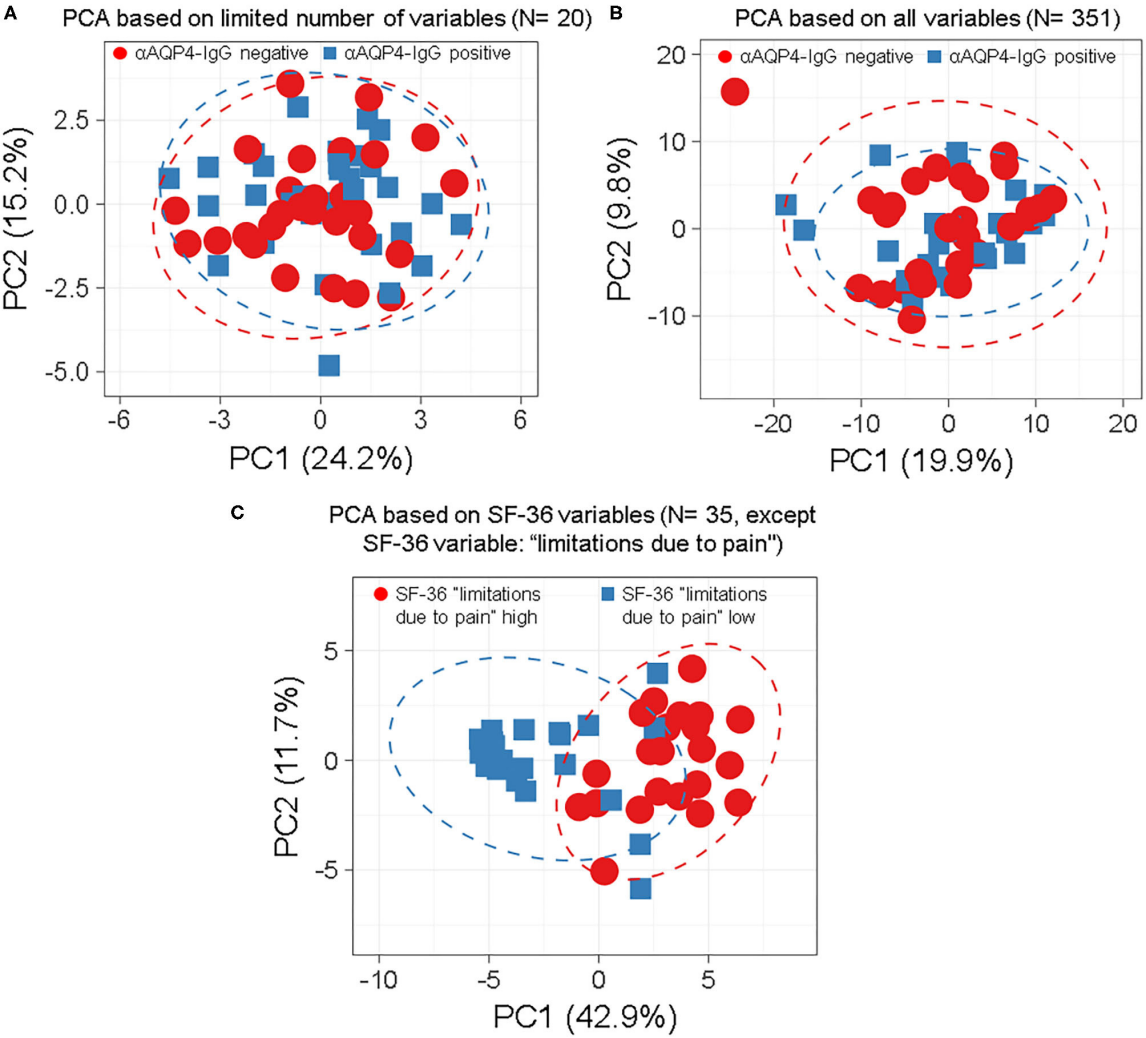
Anti-aquaporin 4 (AQP4)-IgG status does not define distinct subgroups of patients by principle-component analyses (PCA) independent by model, number or type of markers used. Anti-AQP4-IgG status does not define a subgroup (such as anti-AQP4-IgG positive and -negative patients) as demonstrated by the full overlap of the 95% prediction ellipses in principle-component analyses (PCA). This is irrespective of how many and which markers are, or which model is used. Patients with Neuromyelitis optica spectrum disorder (NMOSD) were colored according to their anti-AQP4-IgG-status **(A,B)** or the second pain variable, describing levels of pain disturbing work during the last 4 weeks (question 22 from the “36-item containing short form health survey,” SF-36 score; in **C**). Singular value decomposition (SVD) with imputation was used here in all PCAs to calculate principal components (PC). Prediction ellipses are such that with a probability of 0.95, a new observation from the same group will fall inside the ellipse. **(A)** A PCA was performed with all variables (but not anti-AQP4 statuses) and the patients were marked according to their anti-AQP4-IgG status. The PCA-plot shows PC1 and PC2, explaining 24 and 15% of the total variance, respectively, and was performed using only a limited number of key variables (*N* = 20) such as the Expanded Disability Status Scale (EDSS), disease activity and severity, sex and age. Prediction ellipses were drawn such that a new observation from the same group will fall inside the ellipse with a probability of 0.95. However, those prediction ellipses as well as the data points completely overlap. No difference based on anti-AQP4-IgG status is visible. **(B)** As in A, but all 351 variables (symptoms, disabilities, lab values, anamnestic, psychosomatic scores, and clinical markers) which have been recorded for the patients are included in the analysis, except for the anti-AQP4 statuses. Again, no difference based on anti-AQP4-IgG status is visible, in marked contrast to **(C)**: Same as in A but shown are patients having high vs. low levels of interference of pain with normal work (including work outside the home and housework) during the past 4 weeks. A PCA based on the remaining 35 variables from the SF-36 score (except the mentioned pain variable two, for which the patient cases have been colored) is shown. Here, the prediction ellipses for a probability of 0.95, as well as the dots *per se*, show a remarkable difference between the two groups. AQP4, aquaporin 4; EDSS, Expanded Disability Status Scale; NMOSD, neuromyelitis optica spectrum disorder; PC, principal components; PCA, principle-component analysis; SF-36, 36-item short form health survey; SVD, singular value decomposition.

**Figure 2:**
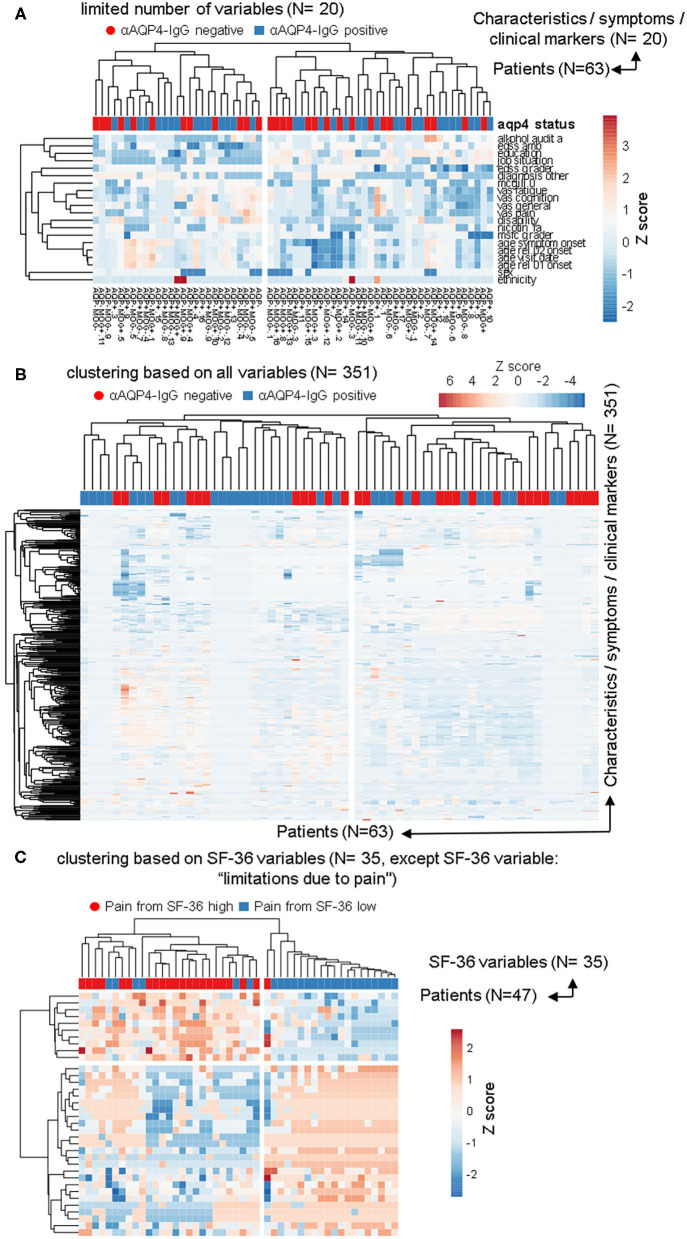
The anti-aquaporin 4 (AQP4)-IgG status does not define a subgroup that would be detectable in heatmap-based clustering analyses. In addition to the PCAs, the same data sets as in Figure 1 were used to perform calculations based on phylogenetic clustering analyses using heatmaps. As before, no difference based on anti-AQP4-IgG status were identifiable. This is in contrast to the two clearly visible different groups, which are based on the second pain variable taken from the 36-item short form health survey (SF-36). **(A,B)** Patients with Neuromyelitis optica spectrum disorder (NMOSD; *N* = 63) were clustered in columns using correlation distance and average linkage based either on only a limited number of key variables, such disease activity, severity, sex and age etc. (**A**; *N* = 20) or based on up to 351 symptoms and clinical markers (**B**; shown here in rows) were clustered. Unit variance scaling is applied to the symptoms and the clinical markers. On top of the rows, anti-AQP4 status is shown. No group based on anti-AQP4-IgG status is visible. **(C)** As in **(A,B)**, only the 35 variables of the SF-36 score were used and the same patients were clustered. In contrast to anti-AQP4-IgG, on the left side of the dendrogram, clearly visible a major group based on a high pain variable was formed. A difference to the anti-AQP4-IgG status can be seen due to the cluster on the left, formed by the residual variables of the SF-36 score. All patients are positive for a high second pain variable. On top of the rows, the second pain variable taken from the SF-36 score is color encoded shown.

We found that anti-AQP4-IgG status does not associate with any subgroup of patients, characterized by patterns of symptoms, patient characteristics, laboratory results, clinical scores or findings (Figures 1, 2; Supplementary Figures 1–7), as demonstrated by overlap of the 95% prediction ellipses in PCA. Here all variables were converted to two most principal components, to be able to plot it in 2D. There were no anti-AQP4-IgG-dependent subgroups of patients identifiable, irrespective of whether a PCA was performed with a basic set of symptoms and clinical markers in the database (*N* = 20; Figure 1A) or with the complete set (351 variables; Figure 1B; Supplementary Figure 1). The anti-AQP4-IgG positive and negative patients form again fully overlapping groups in all 57 cluster analyses performed for this study, independent of PCA method, model used, as well as type or amount of input data. Only two of the 57 models were exemplary, shown in Figure 1. There was also a near full matching of the 0.95 CI prediction ellipses of anti-AQP4-IgG positive and negative patients in the PCAs. To show a contrary example, two distinct groups of patients could be defined using the variables of the “36-item containing short form health survey” (SF-36 score). The best group-defining single value from those 36 variables was the second pain variable (question 22) of this score, assessing the level of interference of pain with normal work (including work outside the home and housework) during the past 4 weeks. Figure 1C shows a PCA based on the remaining 35 variables taken from this score, except the mentioned pain variable two, for which the patient cases have been colored. Patients having high (values 50, 75 and 100) vs. low levels (values 0 and 25) of this work disturbing pain therefore form two different subgroups in the PCA (based on the remaining 35 variables) with largely non-overlapping 0.95 CI prediction ellipses. This finding is independent of model and data used.

We used the same datasets in Figure 2 for heatmap-based phylogenetic clustering analyses. Again, a low number of 20 variables (Figure 2A) as well as a complete set of our database (Figure 2B) leads to two major groups, which are independent of anti-AQP4-IgG status (shown in red and blue on top of the heatmaps). In Figure 2A the patient group in the left main cluster are characterized by a higher age at disease onset, a higher degree of unemployment and disability, lower levels of education, higher scores in pain and fatigue questionnaires as compared to the cluster on the right side. In contrast, the patients in the right main cluster were younger at disease onset, report lower pain levels, but score worse in functional scores such as EDSS and MSFC.

In contrast, phylogenetic cluster analysis e.g., based only on the variables from the SF-36 score as done for Figure 1C leads to separation of two groups (Figure 2C): One mainly with high levels of work disturbing pain (Figure 2 left side) and a group of patients where all but one have low levels of the pain variable 2 (Figure 2 right side).

The anti-AQP4-IgG positive and negative patients form almost always fully heterogeneous groups in all 57 cluster analyses performed for this study, independent of clustering method, model used, as well as type or amount of input data (Figures 2A,B; Supplementary Figures 1–5).

Some subgroups roughly related only to a negative anti-AQP4-IgG status could be found, but only with certain, uncommon clustering methods (marked with green boxes in Supplementary Figure 6) and if the number of variables were extraordinarily reduced to 18. Specifically, only removal of all information dealing with relapses, drugs, therapies and response to therapy, all variables from more extensive clinical score details (which investigated single symptom areas such as motor function, vision, psychological scores, detailed characteristics of pain etc.), all imaging data, as well as by removing all data about additional other diagnoses led to formation of these single subgroups with a negative anti-AQP4-IgG status. Those remaining 18 variables consisted mainly of a set of limited biographical data, while only the age at symptom onset and diagnosis, the disability level and selected EDSS score results were the remaining disease relevant markers. As the more severely ill patients still always formed subgroups with completely mixed anti-AQP4 status, this finding represents very likely a stochastic artifact due to a high number of clustering attempts.

### Anti-AQP4-IgG-Titer Does Not Correlate With Disease Activity

**Figure 3:**
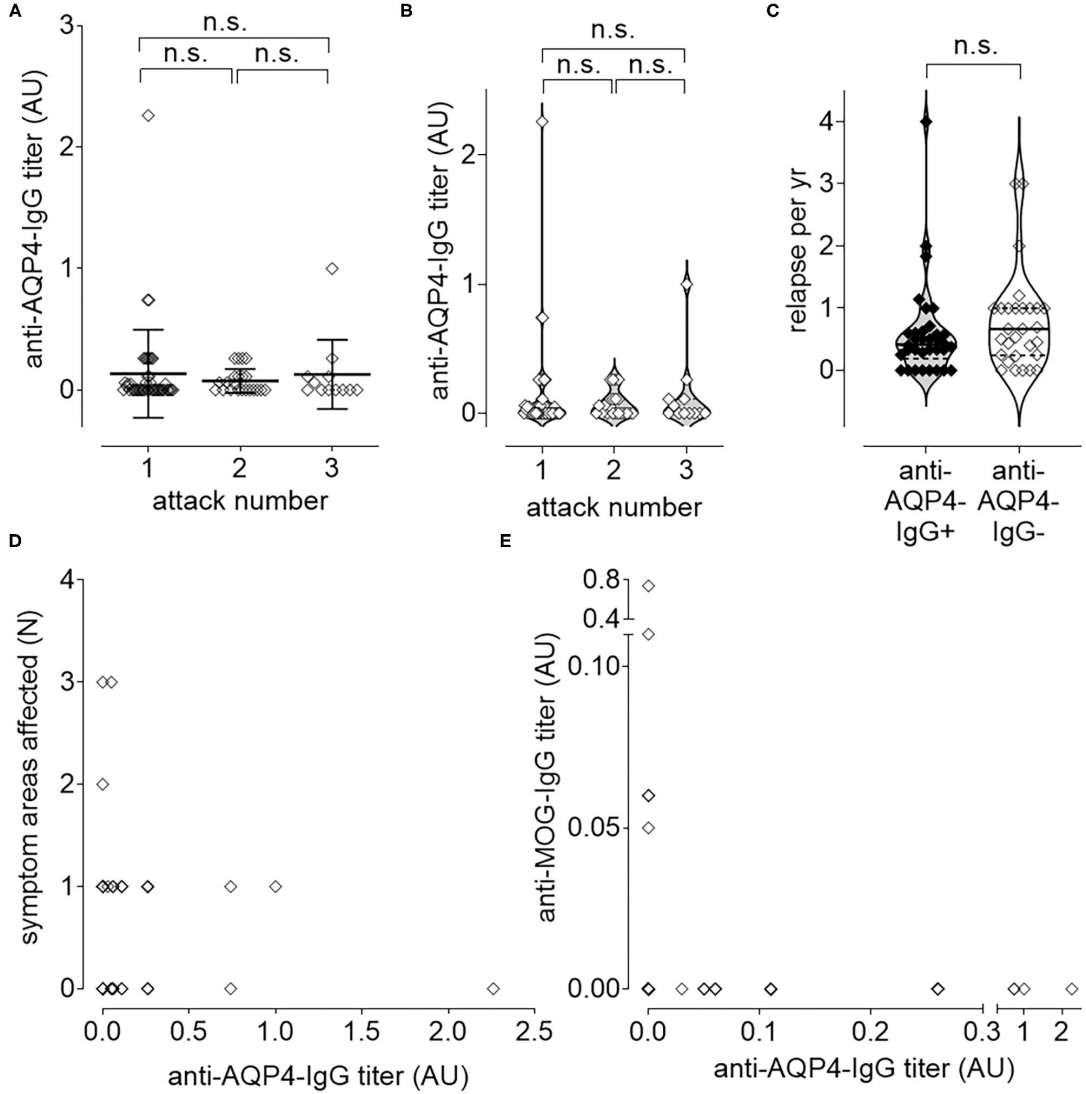
Longitudinal causal interference analysis: anti-AQP4-IgG-Titer does not correlate with disease activity or progression. The level of anti-AQP4-IgG antibody titers remain unchanged after increased number of attacks **(A,B)**. Also, the number of relapses per year (ARR) do not correlate with the anti-AQP4-IgG status **(C)**. The level of anti-AQP4-IgG titers are not increased if more symptom areas are affected **(D)**. A dichotomy can be seen if the level of anti-AQP4-IgG is plotted vs. the anti-MOG-IgG titer **(E)**: NMOSD patients which are anti-MOG-IgG positive do not have anti-AQP4-IgG and vice versa. AU, arbitrary unit.

Anti-AQP4-IgG titers were determined as described previously (36), but did not increase significantly with increased number of attacks (Figures 3A,B) nor with increased disease activity, measured as increased number of relapses per year (ARR; Figure 3C). There was also no significant increase of the titer with the number of affected symptom areas as a surrogate for disease severity (Figure 3D). As higher disease activity (defined as increased number of ARR) was not associated with an increase of anti-AQP4-IgG titers, a biological gradient was not apparent. Even if no significant change of anti-AQP4-IgG levels could be identified after two relapses, a non-significant increase in the mean anti-AQP4-IgG titer was visible with multiple relapses. The mean titers (from 0.075 to 0.128), but not of the median, increased after the third relapse as compared to the second one. In addition, a non-significant increase in the median could be noted between relapse one and two (from 0 to 0.025), but no increase was found in the mean anti-AQP4-IgG levels.

Mean titer of anti-AQP4-IgG after attack 1 was 0.134 (median 0; range 0–2.26; SD 0.362; 95% CI 0.026–0.241; *N* = 46), after attack 2 it was 0.075 (median 0.025; range 0—.26; SD 0.099; 95% CI 0.031–0.119; *N* = 22) and after attack 3 0.128 (median 0; range 0–1.00; SD 0.2857; 95% CI −0.053–0.309; *N* = 12). There was no increase in titer, if the first attack was compared with the second (*P* = 0.365; 95% CI −0.239–0.575) or the second with the third attack (*P* = 0.803; 95% CI −0.517–0.626). Also, after two attacks there was no significant difference between the titer from attack 1 and 3 (*P* = 0.606; 95% CI −0.676–0.451; Figures 3A,B).

In addition, the ARR did not differ significantly (*P* = 0.351; 95% CI −0.206–0.571) between anti-AQP4-IgG positive (Mean 0.590; Median 0.414; range 0–4.000; SD 0.764; *N* = 34) and negative (Mean 0.773; Median 0.667; range 0–3.000; SD 0.774; *N* = 29) patients (Figure 3C).

The number of symptom areas affected (mean 0.563; median 0.500; range 0–3.000; SD 0.653; 95% CI 0.417–0.708; *N* = 80) did not correlate (Pearson *r* = −0.050; *P* = 0.657) with the titer of anti-AQP4-IgG (mean 0.117; median 0.000; range 0–2.260; SD 0.299; 95% CI 0.050–0.183; *N* = 80; Figure 3D). The number of attacks (mean 1.575; median 1.000; range 1–3; SD 0.743; 95% CI 1.410–1.740; *N* = 80) did also not correlate (Pearson *r* = −0.036; *P* = 0.750) with the titer of anti-AQP4-IgG (not shown).

NMOSD patients were either anti-MOG-IgG or anti-AQP4-IgG single positive, or double negative (Figure 3E).

The titer of anti-AQP4-IgG (mean 0.121; median 0.000; range 0–2.260; SD 0.204; 95% CI 0.052–0.190; *N* = 77) did not correlate (Pearson *r* = −0.066; 95% CI −0.286–0.160; *P* = 0.568) with the titer of anti-MOG-IgG (mean 0.014; median 0.000; range 0–0.740; SD 0.086; 95% CI −0.005–0.033; *N* = 77; Figure 3E).

In addition to the cluster analyses and PCAs, we could not identify any difference related to anti-AQP4-IgG in more classical analyses of various markers of disease history and severity, including age at relapses, treatment plans, pain, visual function, fatigue and psychometric scores (Supplementary Figure 7).

There was no significant difference in the temporal characteristics of the history of disease (disease onset, diagnosis, start and stop of therapies, ages at the first visit in our clinic as well as ages at the relapses) for Anti-AQP4-IgG positive and negative patients.

In full detail, in the “mean age at symptom onset (years)” with a *P* of 0.54 (mean age 41.82 vs. 39.37 years (yrs); median 42.50 vs. 39.00 a; range 14–65 vs. 7–70; SD 14.68 vs. 16.37; 95% CI 36.70–46.95 vs. 32.89–45.85; *N* = 34 vs. 27), in the “mean age at first diagnosis (years)” with a *P* of 0.84 (mean age 43.42 vs. 44.22 yrs; median 43.00 vs. 44.00 a; range 15–67 vs. 17–79; SD 14.26 vs. 15.51; 95% CI 38.37–48.48 vs. 38.09–50.36; *N* =3 3 vs. 27), in the “mean age at start of current therapy (years)” with a *P* of 0.72 (mean age 45.32 vs. 43.82 ysr; median 45.00 vs. 47.00 a; range 15–68 vs. 20–72; SD 14.23 vs. 15.05; 95% CI 39.80–50.84 vs. 37.15–50.49; *N* = 28 vs. 21), in the “mean age at start of first therapy (years)” with a *P* of 0.98 (mean age 44.15 vs. 44.00 yrs; median 41.50 vs. 45.00 a; range 20–67 vs. 19–70; SD 13.33 vs. 15.30; 95% CI 37.91–50.39 vs. 35.17–52.83; *N* = 20 vs. 14), in the “mean age at stop of current therapy (years)” with a *P* of 0.85 (mean age 46.10 vs. 45.21 yrs; median 44.00 vs. 46.00 a; range 28–68 vs. 20–71; SD 11.63 vs. 15.14; 95% CI 40.65–51.55 vs. 36.47–53.95; *N* =2 0 vs. 14), in the “mean age at first visit date (years)” with a *P* of 0.54 (mean age 49.04 vs. 46.50 yrs; median 50.00 vs. 48.50 a; range 21–73 vs. 21–79; SD 14.28 vs. 15.43; 95% CI 43.39–54.68 vs. 40.27–52.73; *N* = 27 vs. 26), in the “mean age at relapse 1 (years)” with a *P* of 0.79 (mean age 41.58 vs. 40.44 yrs; median 42.50 vs. 39.00 a; range 14–66 vs. 7–70; SD 14.31 vs. 15.98; 95% CI 35.80–47.36 vs. 33.85–47.03; *N* = 26 vs. 25), in the “mean age at relapse 2 (years)” with a *P* of 0.67 (mean age 44.38 vs. 42.25 yrs; median 46.00 vs. 44.00 a; range 14–66 vs. 9–70; SD 15.07 vs. 16.94; 95% CI 37.52–51.24 vs. 34.32–50.18; *N* = 21 vs. 20) or in the “mean age at relapse 3 (years)” with a *P* of 0.36 (mean age 43.25 vs. 37.33 yrs; median 43.50 vs. 40.50 a; range 15–66 vs. 10–67; SD 15.86 vs. 17.23; 95% CI 34.80–51.70 vs. 26.38–48.28; *N* = 16 vs. 12) as shown in Supplementary Figure 7A.

There was also no significant difference for Anti-AQP4-IgG positive and negative patients regarding different treatments. Anti-AQP4-IgG positive and negative patients did not differ significantly in receiving Rituximab (mean 0.56 vs. 0.38 cycles; median 1.00 vs. 0; range 0-1 for both; SD 0.50 vs. 0.49; 95% CI 0.38–0.73 vs. 0.19–0.57; *N* = 34 vs. 29) with a *P* of 0.16, Azathioprine (mean 0.15 vs. 0.14 cycles; median 0 for both; range 0-1 for both; SD 0.36 vs. 0.35; 95% CI 0.02–0.27 vs. 0.00–0.27; *N* = 34 vs. 29) with a *P* of 0.92 or Mycophenolate mofetil (mean 0.03 vs. 0.07 cycles; median 0 for both; range 0-1 for both; SD 0.17 vs. 0.26; 95% CI −0.03–0.09 vs. −0.03–0.17; *N* = 34 vs. 29) with a *P* of 0.47 (Supplementary Figure 7B).

We could also not identify any significant difference in pain as well as in visual function scores. Anti-AQP4-IgG positive and negative patients demonstrated no difference in the “McGill Pain Questionnaire (MPQ)” (mean 30.50 vs. 28.69; median 36 vs. 29; range 6–55 vs. 5–50; SD 14.38 vs. 15.61; 95% CI 22.84–38.16 vs. 19.26-38.12; *N* = 16 vs. 13) with a *P* of 0.75 (passed Test for normal distribution), in the “painDETECT questionnaire (PDQ)” (mean 28.10 vs. 26.15; median 28 vs. 20; range 2–49 vs. 9–63; SD 14.99 vs. 15.93; 95% CI 21.27–34.92 vs. 18.70–33.60; *N* = 21 vs. 20) with a *P* of 0.69 (passed Test for normal distribution), in the “Brief Pain Inventory (BPI)” (mean 34.50 vs. 35.64; median 35.5 vs. 28; range 10–54 vs. 2–78; SD 15.71 vs. 27.09; 95% CI 21.36–47.64 vs. 17.44–53.83; *N* = 8 vs. 11) with a *P* of 0.92 (passed Test for normal distribution), in the “visual analog scale (VAS) pain” (mean 35.43 vs. 31.88; median 34.0 vs. 26.5; range 0–100 vs. 0–98; SD 29.78 vs. 28.35; 95% CI 22.56–48.31 vs. 19.90–43.85; *N* = 23 vs. 24) with a *P* of 0.54 (not normal distributed), in the “National Eye Institute Visual Function Questionnaire (NEI-VFQ)” (mean 107.6 vs. 113.5; median 109.0 vs. 113.0; range 58–124 vs. 104–129; SD 12.88 vs. 7.20; 95% CI 102.0–113.1 vs. 110.5–116.5; *N* = 23 vs. 25) with a *P* of 0.10 (not normal distributed) or in the composite “National Eye Institute Visual Function Questionnaire (NEI-VFQ) and Neuro-Ophthalmic Supplement (NOS)” (mean 47.00 vs. 45.04; median 49 vs. 46; range 25–54 vs. 29–56; SD 6.82 vs. 6.03; 95% CI 44.05–49.95 vs. 42.55–47.53; *N* = 23 vs. 25) with a *P* of 0.30 (normal distributed) as shown in Supplementary Figure 7C.

We also compared disability status Scales and could not identify a difference. Anti-AQP4-IgG positive and negative patients demonstrated no significant difference in the “Multiple Sclerosis Functional Composite (MSFC)” with a *P* of 0.68 (mean 7.96 vs. 8.21; median nine for both; range 4–9 for both; SD 2.07 vs. 1.87; 95% CI 7.08–8.83 vs. 7.31–9.11; *N* = 24 vs. 19) as shown in Supplementary Figure 7D.

There was no significant difference for Anti-AQP4-IgG positive and negative patients in the “The Short Form (36) Health Survey (SF-36)” with a *P* (passed Test for normal distribution) of 0.56 (mean 97.78 vs. 99.04; median 99 vs. 101; range 80–112 vs. 81–108; SD 7.99 vs. 7.01; 95% CI 94.33–101.2 vs. 96.15–101.9; *N* = 23 vs. 25), in the “visual analog scale (VAS). general” with a *P* (normal distributed) of 0.35 (mean 39.39 vs. 46.38; median 35 vs. 57.5; range 0–100 vs. 0–82; SD 27.06 vs. 23.91; 95% CI 27.69–51.09 vs. 36.28–56.47; *N* = 23 vs. 24), in the “visual analog scale (VAS) cognition” with a *P* (passed Test for normal distribution) of 0.65 (mean 31.61 vs. 31.70; median 25 vs. 26; range 0–98 vs. 0–69; SD 30.11 vs. 24.40; 95% CI 18.59–44.63 vs. 21.15–42.25; *N* = 23 for both), in the “fatigue severity scale (FSS)” with a *P* (data was normal distributed) of 0.89 (mean 34.65 vs. 35.36; median 30 vs. 33; range 9–63 vs. 10–63; SD 18.70 vs. 15.19; 95% CI 26.57–42.74 vs. 29.09–41.63; *N* = 23 vs. 25), in the “Fatigue Scale for Motor and Cognitive Functions (FSMC)” with a *P* (passed Test for normal distribution) of 0.96 (mean 36.90 vs. 36.58; median 29 vs. 38.5; range 4–76 vs. 1–69; SD 21.24 vs. 19.75; 95% CI 27.23–46.57 vs. 28.25–44.92; *N* = 21 vs. 24), in the “Beck Depression Inventory (BDI)” with a *P* (normal distributed data) of 0.30 (mean 9.32 vs. 11.82; median 7.5 vs. 10; range 1–27 for both; SD 7.42 vs. 8.36; 95% CI 6.03–12.61 vs. 8.11–15.52; *N* = 22 for both) or in the “visual analog scale (VAS) fatigue" with a *P* (results fitted to a normal distribution) of 0.22 (mean 36.61 vs. 46.67; median 23 vs. 54.5; range 0–100 vs. 0–76; SD 29.15 vs. 26.16; 95% CI 24.00–49.22 vs. 35.62–57.71; *N* = 23 vs. 24) as shown in Supplementary Figure 7E.

Even if a reduction of the Anti-AQP4-IgG titers was visible, there was no statistically significant difference (*P* = 0.85; no normal distribution) in the titer of Anti-AQP4-IgG between rituximab treated patients and untreated patients (mean 0.09 vs. 0.19; median 0 for both; range 0–0.74 vs. 0–2.26; SD 0.17 vs. 0.51; 95% CI 0.02–0.16 vs.−0.04–0.42; *N* = 25 vs. 21) as shown in Supplementary Figure 7F.

However, we identified a close to significant (*P*=0.09) increased rate of malignomas in the anti-AQP4-IgG negative as compared to the positive group (mean 0 vs. 0.10 malignomas; median was 0 in both cases; range 0 vs. 0–1; SD 0 vs. 0.31; 95% CI 0–0 vs.−0.014–0.221; *N* = 34 vs. 29; not shown). Further analysis showed that this increased rate in the anti-AQP4-IgG negative group was not present in anti-MOG-IgG positive patients, but only in anti-MOG-IgG negative patients (mean 0 vs. 0.20 tumors; median was 0 in both; range 0 vs. 0–1; SD 0 vs. 0.42; 95% CI 0-0 vs.−0.102–0.502; *N* = 17 vs. 10). However, this was even less significant (*P* = 0.13; not shown).

### Positive Anti-aquaporin 4 (AQP4)-IgG Status Is Associated With Other Autoimmune Diseases in Addition to NMOSD

**Figure 4:**
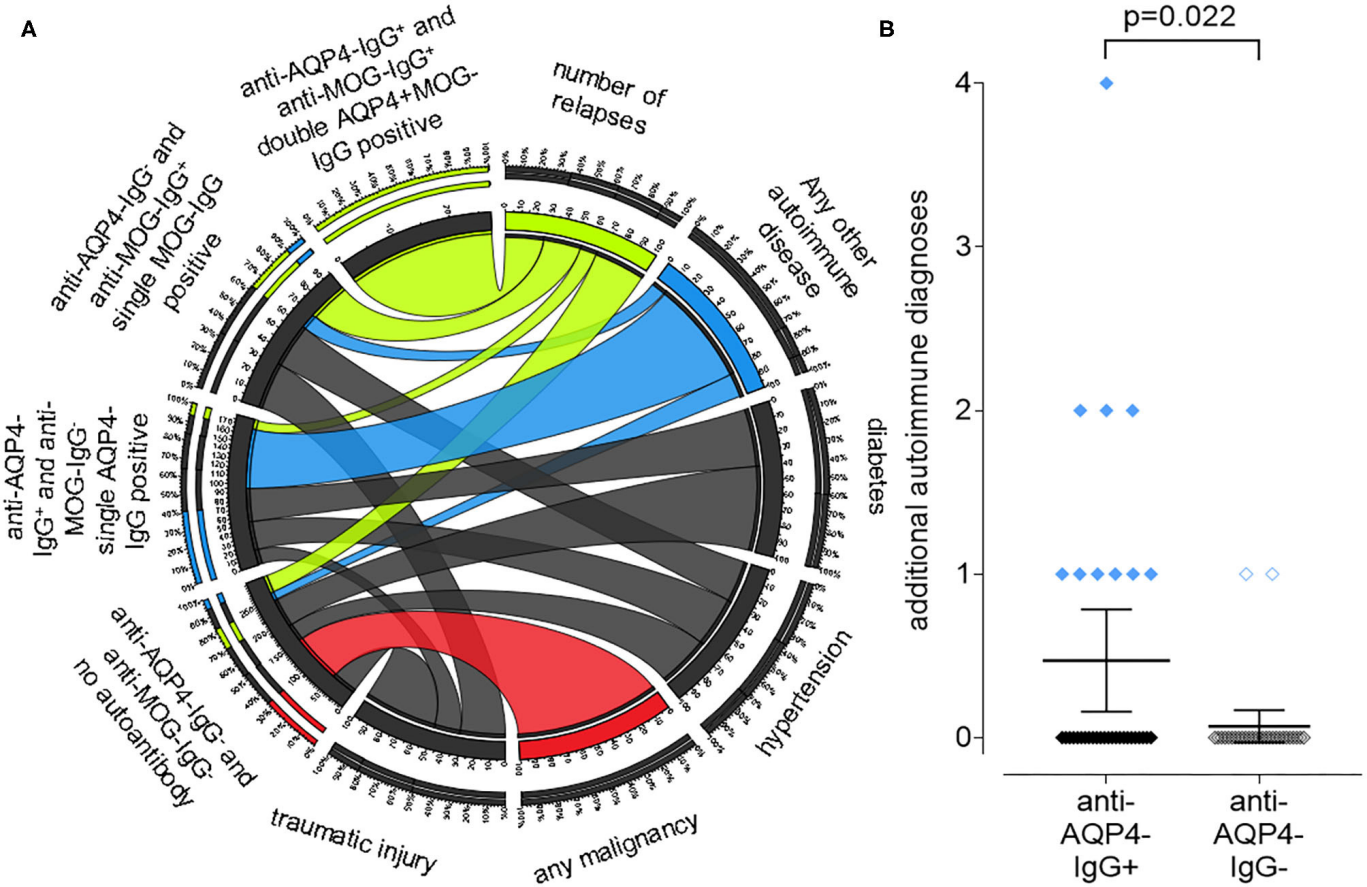
A positive anti-aquaporin 4 (AQP4)-IgG status is associated with the presence of other autoimmune diseases in addition to NMOSD, while a negative status is associated with malignancies. A chord plot, based on the 59 patients for which we had both, anti-AQP4- and anti-MOG-IgG test results, is shown **(A)**. The patients were sorted according to these results into four groups, shown on the left side of the chord plot: anti-AQP4-IgG^+^ and anti-MOG-IgG^+^: double AQP4+MOG-IgG positive anti-AQP4-IgG^−^ and anti-MOG-IgG^+^: single MOG-IgG positive anti-AQP4-IgG^+^ and anti-MOG-IgG^−^: single AQP4-IgG positive anti-AQP4-IgG^−^ and anti-MOG-IgG^−^: no aAb. On the right side, key findings such as: number of relapses; any other autoimmune disease (thyroiditis; SLE; myasthenia; Sjogren's and other); diabetes; hypertension; any malignancy and traumatic injury are show. The thickness of the connecting chords between the four patient subgroups and the key findings represents the number of patients with each of those conditions. The chords connecting the patients with “any malignancy” have been colored red, however there is only one chord present, connecting “any malignancy” with the “anti-AQP4-IgG^−^ and anti-MOG-IgG^−^: no aAb” group. Therefore, all patients with malignancy do not have any of the aAbs. In contrast, the chords connecting patients with “any other autoimmune disease” have been colored blue. The thickest chord, representing about 80% of the autoimmune group, is connected to the “single AQP4-IgG positive” patients. Vice versa, in this “single AQP4-IgG positive” group, the connection to the autoimmune group represents about 40% of all its connections. Therefore, 80% of all patients with a second autoimmune condition were single AQP4-IgG positive and in this group, it was the largest contributing factor and showed a significant difference related to the AQP4-IgG status **(B)**.

We identified a high number of additional diagnoses in addition to NMOSD in our patient collective (Figure 4A). While double aAb, anti-AQP4- and anti-MOG-IgG-negative patients had a higher rate of malignancies, anti-AQP4-IgG^+^ patients had a significantly (*P* = 0.022; Mann–Whitney test as not normal distributed) higher rate of additional autoimmune diagnoses as compared to anti-AQP4-IgG-negative patients (mean 0.47 in AQP4-IgG^+^ vs. 0.07 in AQP4-IgG^−^; median was 0 in both; range 0–4 vs. 0–1; SD 0.90 vs. 0.26; 95% CI 0.16–0.78 vs.−0.03–0.17; *N* = 34 vs. 29; Figure 4B). Ten of the 34 anti-AQP4-IgG^+^ patients (29%) had at least one additional autoimmune disorder as compared to only two of the 29 anti-AQP4-IgG^−^ patients (7%). Of those ten anti-AQP4-IgG^+^ patients, three had two and one had even four additional autoimmune diseases.

The two leading additional autoimmune disease were autoimmune thyroiditis in 4 and systemic lupus erythematosus (SLE) in five of the anti-AQP4-IgG^+^ patients (Supplementary Table 2). In contrast, none of the anti-AQP4-IgG^−^ patients had SLE and only one patient had autoimmune thyroiditis.

## Discussion

In this study, anti-AQP4-IgG positive or negative subgroups are not clearly associated with clinical parameters. The observation that patients without anti-AQP4-IgG are not clinically distinguishable from those patients with detected anti-AQP4-IgG argues challenges the hypothesis that anti-AQP4-IgG plays a major disease-driving role. However, we cannot exclude that the autoantibody assays were made at the wrong time point over the course of the disease. We used the data at baseline, the first visit of the patient as it might change after relapse therapy e.g., with rituximab.

This indiscernibility might be explained by either of the following: (1) with anti-AQP4-IgG we detect a polyreactive aAb and we know only one, maybe a minor, low affinity target of this aAb; (2) Anti-AQP4-IgG is a secondary, disease sustaining aAb (like in rheumatic fever), but not initiating it; patients can still benefit clinically from targeting it; (3) Anti-AQP4-IgG is an indicator for a yet unknown disease subgroup; or (4) Anti-AQP4-IgG is an epiphenomenon without salient clinical relevance for the course of NMOSD.

Emerging research suggests that autoimmune diseases, even when mediated by T cells, will frequently accompany the emergence of aAb to the same, often intracellular autoantigens (aAgs) (37, 38). aAbs, whose serum levels correlate with disease severity, have the potential to be used not only as biomarkers but also to identify relevant T cell targets (39–41). Nevertheless, the diversity and polyreactivity of aAbs in individuals with an autoimmune condition poses a significant barrier to determining which aAgs are pathogenic.

According to the Bradford Hill criteria, a biological gradient should be present, to prove the causal role of a presumed cause and an observed effect (disease) (42). This missing biological gradient, the missing increase of the anti-AQP4-IgG with increasing disease activity, is hard to explain. In other autoimmune disease such as Lupus nephritis, there is a clear correlation of severity of the clinical disease with anti-dsDNA-autoantibodies (43, 44).

“Bystander” B cell responses have been observed in several autoimmune diseases, Hashimoto's thyroiditis being a good example. In Hashimoto's, high amounts of anti-thyroid aAb are produced and correlate well with the clinical course (45). These aAb can even fix complement and facilitate the destruction of thyroid cells (46, 47). However, they are a consequence of the disease process, not a cause (48, 49). Another example is polymyositis, where anti-myoglobin aAbs correlate well with clinical activity, but do not participate in the induction of tissue destruction (50). Whether aAbs play a pathogenic role in MS, a comparable CNS disease, remains unclear; but even when they lack a causative role in disease, aAbs nevertheless inform us about the identity of the aAgs that are available and recognized by the autoimmune T cell population. Many aAb are IgG type natural occurring antibodies (NAbs), which are present from birth and not induced later (51, 52). NAbs do not appear to depend on CD4 T cell help, noteworthy because anti-CD4 is ineffective in MS (53, 54). Identifying the targets of NAbs is technically challenging. NAbs purified with immunoadsorbent columns with one aAg are often cross-reactive to other aAgs (51). Polyreactivity is a big problem, as we need to identify the set of best binding aAgs for each monoclonal Ab, select the disease-relevant targets and define possible pathogenic T cell epitopes of those aAgs (55). Nevertheless, we have extensive experience in aAb characterization and have developed protocols that overcome many of these challenges (56, 57).

The initiation of disease, how the blood-brain barrier (BBB) breaks down, is left unexplained. AQP4, the six transmembrane helix water channel, is not expressed on endothelial cells, therefore translocation of anti-AQP4-IgG through the BBB, due to a leaky BBB and/or local production of it is required (58). Those aAbs are thought to be of high pathophysiologic if not etiologic relevance even as a myriad of other aAgs have now been found in several major neurological diseases (37). As NMOSD is thought to be caused by an autoimmune reaction and as patients with NMOSD experience almost exclusively symptoms due to CNS involvement, an expression of the target of this autoimmune reaction limited selectively to the CNS would fortify the pathological hypothesis. Many new isoforms of AQP4 just have been identified (Supplementary Figure 8) with broad expression of the main transcript outside the CNS. Expression data of AQP4: ENSG00000171885.13 from HGNC (Acc: HGNC: 637) shows eight splice variants (Supplementary Figure 8), which represent different protein isoforms, the major one is also highly expressed in the lungs, thyroid, gastrointestinal (GI) system and other organs. Proteomics data reveals an AQP4 protein presence in lung and stomach which is comparable to its presence in astrocytes. However, in patients with anti-AQP4-IgG, pulmonary and gastric symptoms have not been described. Yet there are studies investigating binding of patients' anti-AQP4-IgG against two of the major AQP4 forms expressed on astrocytes (59), although others have proposed that anti-AQP4-IgG may not be the main direct cause of the astrocytopathy in NMOSD, but demonstrated that anti-AQP4-IgG down-regulates CXC motif ligand 12 (CXCL12) and impairs remyelination by oligodendrocyte progenitor cells (60). Indeed, the high degree of heterogeneity of the patients' IgGs to targeting different isoforms of AQP4 raises the question how well other isoforms are targeted, especially in non-CNS organs.

However, there are four CNS-specific transcripts, of which transcript six might be functional and exclusively use exon two, making it an ideal target for a CNS-selective aAb leading to a localized autoimmune reaction in NMOSD. In further studies, we should also test those isoforms, splice variants as well as other genotypes of AQP4 as target of the aAb. We cannot exclude that anti-AQP4-IgG plays a prominent role at an earlier or later stage, especially as the expression is heavily modified by inflammation (especially IFNγ) (61).

AQP4 represents only one of many so far described IgG aAgs in NMOSD such as anti-nuclear and anti-Ro aAbs (1, 62). Thus, it is possible that several autoreactive IgG against several targets collectively contribute to disease. The recent positive trial data demonstrating efficacy of anti-CD19 in NMOSD indirectly supports this notion (23, 63), although it should be noted that B cells serve additional functions beyond merely manufacturing IgG (64). Interestingly, we found no difference in anti-AQP4 status after treatment with rituximab, to which most patients responded with a clinical improvement. Similar as what has been described in other autoimmune diseases, a reduction in anti-AQP4 status would have been conceivable (65). This might be a further hint that the role of anti-AQP4-IgG as disease driving aAb has been overestimated.

The finding that a positive anti-AQP4-IgG status is significantly associated with the presence of other autoimmune diseases in addition to NMOSD might indicate that anti-AQP4 is indeed a “bystander” B cell response as explained above. Therefore, in some patients detectable anti-AQP4-IgG titers may signify the co-occurrence of NMOSD and additional autoimmune disease. In our patient cohort, five patients suffered from additional SLE, four from autoimmune thyroiditis and two from Sjogren's syndrome. This pattern fits in with the long-known association of NMOSD with these autoimmune syndromes, which first led to the discovery of NMO-IgG and turned out to be anti-AQP4-IgG (1). Interestingly, pathological changes in the IgG half-life have been described in all of these diseases, so that certain aAbs can be present at elevated titers (66–69). In addition, B cell survival and activation is enhanced in those autoimmune diseases (70). This might give a hint that anti-AQP4-IgG might be present in other NMOSD patients, but below the detection limits of the test as additional immune pathologies are needed to make it detectable with the current clinically used test procedures and available immunoassays. So far, our results challenge the assumption that anti-AQP4-IgG alone plays a disease-driving role in NMOSD as it is determined with the currently used immunoassays. We cannot finally rule out that Anti-AQP4-IgG might be the main pathologic factor in a subset of patients, but our analyses rather indicate that it is represent as an epiphenomenon or may be one of many immune mechanisms that collectively contribute to the pathogenesis of this disease.

In further studies, we would suggest evaluating the tissue specific expression of the target, it's splice variants, as well as a possible polyreactivity of Anti-AQP4-IgG. We would also suggest to use *ex vivo* translated AQP4 or more selective certain peptide epitopes to improve the capability of the immunoassay.

## Capsule Summary

We performed phylogenetic clustering, PCA and causal interference analyses to test for a role of anti-AQP4-IgG in 63 comparable patients suffering from NMOSD. Our results challenge the current concept that anti-AQP4-IgG plays a sole or predominant disease-driving role in many patients with this disease.

## Data Availability Statement

The original contributions presented in the study are included in the article/Supplementary Material, further inquiries can be directed to the corresponding author/s.

## Ethics Statement

All data collected from patients and investigated in this study was used after written informed consent as approved by the ethics committee of the Charité - Universitätsmedizin Berlin (proposal EA1/041/14) according to the Declaration of Helsinki (59th WMA General Assembly, Seoul, October 2008). The patients/participants provided their written informed consent to participate in this study.

## Author Contributions

OS did the main research and wrote the manuscript. EL and BR helped correcting the manuscript. AD, SA, FP, and NS helped collecting the patients' materials. FP and NS helped correcting the manuscript. All authors contributed to the article and approved the submitted version.

## Conflict of Interest

BR is presently an employee at Novartis Institutes for BioMedical Research. Novartis did not fund the study. AD received a speaker honorarium from Roche. FP served on the scientific advisory boards of Novartis and MedImmune; received speaker honoraria and travel funding from Bayer, Novartis, Biogen, Teva, Sanofi-Aventis/Genzyme, Merck Serono, Alexion, Chugai, MedImmune, and Shire; serves as academic editor of PLoS ONE and associate editor of Neurology: Neuroimmunology & Neuroinflammation; consulted for Sanofi-Genzyme, Biogen, MedImmune, Shire, and Alexion; and received research support from Bayer, Novartis, Biogen, Teva, Sanofi-Aventis/Genzyme, Alexion, Merck Serono, German Research Council, Werth Stiftung of the City of Cologne, German Ministry of Education and Research, Arthur Arnstein Stiftung Berlin, EU FP7 Framework Program, Guthy Jackson Charitable Foundation, and NMSS. NS received travel funding from Sanofi-Aventis/Genzyme and speaker honoraria from Bayer AG. The remaining authors declare that the research was conducted in the absence of any commercial or financial relationships that could be construed as a potential conflict of interest.

## Abbreviations

ARR: annual relapse rate
AD: Alzheimer's disease
aAgs: autoantigens
AU: arbitrary unit
APC: antigen-presenting cell
AQP4: aquaporine-4
ARR: annualized relapse rate
aAbs: autoantibodies
NMOSD: Neuromyelitis optica spectrum disorder
BBB: blood-brain barrier
C5: complement factor 5
CNS: central nervous system
CXCL12: CXC motif ligand 12
dbGaP: database of Genotypes and Phenotypes
EDSS: Expanded Disability Status Scale
GI: gastrointestinal
GTEx: Genotype-Tissue Expression
HGNC: HUGO gene nomenclature committee
HUGO: Human Genome Organization
IgG: immunoglobulin G
MOG: myelin oligodendrocyte glycoprotein
MRI: Magnetic resonance imaging
MSFC: Multiple Sclerosis Functional Composite
Nabs: natural occurring antibodies
NGS: next generation sequencing
SF-36 score: 36-item containing short form health survey
SVD: Singular Value Decomposition
ON: optic neuritis
OCT: Optical coherence tomography
PCA: Principal component analysis
PC: Principal components
SC: spinal cord.

## References

[1] Wingerchuk DM Lennon VA Lucchinetti CF Pittock SJ Weinshenker BG. The spectrum of neuromyelitis optica. Lancet Neurol. (2007) 6:805–15. 10.1016/S1474-4422(07)70216-8 17706564

[2] Asgari N Lillevang ST H.Skejoe PB Kyvik KO. Epidemiology of neuromyelitis optica spectrum disorder in Denmark. (1998-2008, 2007-2014). Brain Behav. (2019) 9:e01338. 10.1002/brb3.1338 31187587 PMC6625475

[3] Mori M Kuwabara S Paul F. Worldwide prevalence of neuromyelitis optica spectrum disorders. J Neurol Neurosurg Psychiatry. (2018) 89:555–6. 10.1136/jnnp-2017-317566 29436488

[4] Hor JY Asgari N Nakashima I Broadley SA Leite MI Kissani N. Epidemiology of neuromyelitis optica spectrum disorder and its prevalence and incidence worldwide. Front Neurol. (2020) 11:501. 10.3389/fneur.2020.00501 32670177 PMC7332882

[5] Jarius S Wildemann B. AQP4 antibodies in neuromyelitis optica: diagnostic and pathogenetic relevance. Nat Rev Neurol. (2010) 6:383–92. 10.1038/nrneurol.2010.72 20639914

[6] Stellmann JP Krumbholz M Friede T Gahlen A Borisow N Fischer K. Immunotherapies in neuromyelitis optica spectrum disorder: efficacy and predictors of response. J Neurol Neurosurg Psychiatry. (2017) 88:639–47. 10.1136/jnnp-2017-315603 28572277 PMC5537514

[7] Jarius S Paul F Weinshenker BG Levy M Kim HJ Wildemann B. Neuromyelitis optica. Nat Rev Dis Primers. (2020) 6:85. 10.1038/s41572-020-0214-9 33093467

[8] Ciccarelli O Cohen JA Reingold SC Weinshenker BG International Conference on Spinal Cord Involvement and Imaging in Multiple Sclerosis and Neuromyelitis Optica Spectrum Disorders. Spinal cord involvement in multiple sclerosis and neuromyelitis optica spectrum disorders. Lancet Neurol. (2019) 18:185–97. 10.1016/S1474-4422(18)30460-5 30663608

[9] Zekeridou A Lennon VA Aquaporin-4 autoimmunity. Neurol Neuroimmunol Neuroinflamm. (2015) 2:e110. 10.1212/NXI.0000000000000110 26185772 PMC4442096

[10] Jarius S Paul F Aktas O Asgari N Dale RC de Seze J. MOG encephalomyelitis: international recommendations on diagnosis and antibody testing. J Neuroinflammation. (2018) 15:134. 10.1186/s12974-018-1144-2 29724224 PMC5932838

[11] Pittock SJ Lucchinetti CF. Neuromyelitis optica and the evolving spectrum of autoimmune aquaporin-4 channelopathies: a decade later. Ann N Y Acad Sci. (2016) 1366:20–39. 10.1111/nyas.12794 26096370 PMC4675706

[12] O'Connell K Hamilton-Shield A Woodhall M Messina S Mariano R Waters P. Prevalence and incidence of neuromyelitis optica spectrum disorder, aquaporin-4 antibody-positive NMOSD and MOG antibody-positive disease in Oxfordshire, UK. J Neurol Neurosurg Psychiatry. (2020) 91:1126–8. 10.1136/jnnp-2020-323158 32576617

[13] Bukhari W Prain KM Waters P Woodhall M O'Gorman CM Clarke L. Incidence and prevalence of NMOSD in Australia and New Zealand. J Neurol Neurosurg Psychiatry. (2017) 88:632–8. 10.1136/jnnp-2016-314839 28550069

[14] Dalakas MC Alexopoulos H Spaeth PJ. Complement in neurological disorders and emerging complement-targeted therapeutics. Nat Rev Neurol. (2020) 16:601–17. 10.1038/s41582-020-0400-0 33005040 PMC7528717

[15] Kinoshita M Nakatsuji Y Kimura T Moriya M Takata K Okuno T. Neuromyelitis optica: passive transfer to rats by human immunoglobulin. Biochem Biophys Res Commun. (2009) 386:623–7. 10.1016/j.bbrc.2009.06.085 19545538

[16] Wu Y Zhong L Geng J. Neuromyelitis optica spectrum disorder: pathogenesis, treatment, and experimental models. Mult Scler Relat Disord. (2019) 27:412–8. 10.1016/j.msard.2018.12.002 30530071

[17] Vincent T Saikali P Cayrol R Roth AD Bar-Or A Prat A. Functional consequences of neuromyelitis optica-IgG astrocyte interactions on blood-brain barrier permeability and granulocyte recruitment. J Immunol. (2008) 181:5730–7. 10.4049/jimmunol.181.8.5730 18832732

[18] Takeshita Y Obermeier B Cotleur AC Spampinato SF Shimizu F Yamamoto E. Effects of neuromyelitis optica-IgG at the blood-brain barrier *in vitro*. Neurol Neuroimmunol Neuroinflamm. (2017) 4:e311. 10.1212/NXI.0000000000000311 28018943 PMC5173350

[19] Hinson SR McKeon A Fryer JP Apiwattanakul M Lennon VA Pittock SJ. Prediction of neuromyelitis optica attack severity by quantitation of complement-mediated injury to aquaporin-4-expressing cells. Arch Neurol. (2009) 66:1164–7. 10.1001/archneurol.2009.188 19752309

[20] Pittock SJ Berthele A Fujihara K Kim HJ Levy M Palace J. Eculizumab in aquaporin-4-positive neuromyelitis optica spectrum disorder. N Engl J Med. (2019) 381: 614–25. 10.1056/NEJMoa1900866 31050279

[21] Pittock SJ Lennon VA McKeon A Mandrekar J Weinshenker BG Lucchinetti CF. Eculizumab in AQP4-IgG-positive relapsing neuromyelitis optica spectrum disorders: an open-label pilot study. Lancet Neurol. (2013) 12:554–62. 10.1016/S1474-4422(13)70076-0 23623397

[22] Nishiyama S Misu T Nuriya M Takano R Takahashi T Nakashima I. Complement-dependent and -independent aquaporin 4-antibody-mediated cytotoxicity in human astrocytes: Pathogenetic implications in neuromyelitis optica. Biochem Biophys Rep. (2016) 7:45–51. 10.1016/j.bbrep.2016.05.012 29114578 PMC5627508

[23] Duchow A Chien C Paul F Bellmann-Strobl J. Emerging drugs for the treatment of neuromyelitis optica. Expert Opin Emerg Drugs. (2020) 25:285–97. 10.1080/14728214.2020.1803828 32731771

[24] Graf J Mares J Barnett M Aktas O Albrecht P Zamvil SS. Targeting B cells to modify MS, NMOSD, and MOGAD: Part 2. Neurol Neuroimmunol Neuroinflamm. (2021) 8:e919. 10.1212/NXI.0000000000000919 33411674 PMC8063618

[25] Graf J Mares J Barnett M Aktas O Albrecht P Zamvil SS. Targeting B cells to modify MS, NMOSD, and MOGAD: part 1. Neurol Neuroimmunol Neuroinflamm. (2021) 8. 10.1212/NXI.0000000000000918 33411674 PMC8063618

[26] Jitprapaikulsan J Fryer JP Majed M Smith CY Jenkins SM Cabre P. Clinical utility of AQP4-IgG titers and measures of complement-mediated cell killing in NMOSD. Neurol Neuroimmunol Neuroinflamm. (2020) 7:e727. 10.1212/NXI.0000000000000727 PMC7286655 35413004

[27] Reindl M Are aquaporin antibody titers useful outcome measures for neuromyelitis optica spectrum disorders? Neurol Neuroimmunol Neuroinflamm. (2020) 7:e759. 10.1212/NXI.0000000000000759 33306483 PMC7286647

[28] Borisow N Mori M Kuwabara S Scheel M Paul F. Diagnosis and treatment of NMO spectrum disorder and MOG-encephalomyelitis. Front Neurol. (2018) 9:888. 10.3389/fneur.2018.00888 30405519 PMC6206299

[29] Narayan R Simpson A Fritsche K Salama S Pardo S Mealy M. MOG antibody disease: a review of MOG antibody seropositive neuromyelitis optica spectrum disorder. Mult Scler Relat Disord. (2018) 25:66–72. 10.1016/j.msard.2018.07.025 30048919

[30] Reindl M Schanda K Woodhall M Tea F Ramanathan S Sagen J. International multicenter examination of MOG antibody assays. Neurol Neuroimmunol Neuroinflamm. (2020) 7:e674. 10.1212/NXI.0000000000000674 32024795 PMC7051197

[31] Schmidt FA Chien C Kuchling J Bellmann-Strobl J Ruprecht K Siebert N. Differences in advanced magnetic resonance imaging in MOG-IgG and AQP4-IgG seropositive neuromyelitis optica spectrum disorders: a comparative study. Front Neurol. (2020) 11:499910. 10.3389/fneur.2020.499910 33101166 PMC7554609

[32] Wingerchuk DM Banwell B Bennett JL Cabre P Carroll W Chitnis T. M.O.D. International Panel for, International consensus diagnostic criteria for neuromyelitis optica spectrum disorders. Neurology. (2015) 85:177–89. 10.1212/WNL.0000000000001729 26092914 PMC4515040

[33] Metsalu T Vilo J. ClustVis: a web tool for visualizing clustering of multivariate data using principal component analysis and heatmap. Nucleic Acids Res. (2015) 43:W566–70. 10.1093/nar/gkv468 25969447 PMC4489295

[34] Lakin E Church MK Maurer M Schmetzer O. On the lipophilic nature of autoreactive ige in chronic spontaneous urticaria. Theranostics. (2019) 9:829–36. 10.7150/thno.29902 30809311 PMC6376472

[35] Krzywinski M Schein J Birol I Connors J Gascoyne R Horsman D. Circos: an information aesthetic for comparative genomics. Genome Res. (2009) 19:1639–45. 10.1101/gr.092759.109 19541911 PMC2752132

[36] Jarius S Ruprecht K Kleiter I Borisow N Asgari N Pitarokoili K. in cooperation with the Neuromyelitis Optica Study, MOG-IgG in NMO and related disorders: a multicenter study of 50 patients. Part 1: Frequency, syndrome specificity, influence of disease activity, long-term course, association with AQP4-IgG, and origin. J Neuroinflammation. (2016) 13:279. 10.1186/s12974-016-0717-1 27788675 PMC5084340

[37] Lancaster E Dalmau J. Neuronal autoantigens–pathogenesis, associated disorders and antibody testing. Nat Rev Neurol. (2012) 8:380–90. 10.1038/nrneurol.2012.99 22710628 PMC3718498

[38] Boronat A Sepulveda M Llufriu S Sabater L Blanco Y Gabilondo I. Analysis of antibodies to surface epitopes of contactin-2 in multiple sclerosis. J Neuroimmunol. (2012) 244:103–6. 10.1016/j.jneuroim.2011.12.023 22245283 PMC4800093

[39] Giovannoni G Ebers G. Multiple sclerosis: the environment and causation. Curr Opin Neurol. (2007) 20:261–8. 10.1097/WCO.0b013e32815610c2 17495618

[40] Swaen G van Amelsvoort L. A weight of evidence approach to causal inference. J Clin Epidemiol. (2009) 62:270–7. 10.1016/j.jclinepi.2008.06.013 18834711

[41] Schmetzer O. (2007). Identifikation und funktionelle Charakterisierung der neuen CD3 splice Variante CD3k in T-Helferzellen nach Stimulation mit heteroklitischen Peptiden. [dissertation/master's thesis]. Humboldt-University Berlin.

[42] Hill AB. The environment and disease: association or causation? Proc R Soc Med. (1965) 58:295–300. 10.1177/003591576505800503 14283879 PMC1898525

[43] Rekvig OP. The anti-DNA antibody: origin and impact, dogmas and controversies. Nat Rev Rheumatol. (2015) 11:530–40. 10.1038/nrrheum.2015.69 26034836

[44] Rekvig OP. Systemic lupus erythematosus: definitions, contexts, conflicts, enigmas. Front Immunol. (2018) 9:387. 10.3389/fimmu.2018.00387 29545801 PMC5839091

[45] McLachlan SM Rapoport B. Autoimmune response to the thyroid in humans: thyroid peroxidase–the common autoantigenic denominator. Int Rev Immunol. (2000) 19:587–618. 10.3109/08830180009088514 11129117

[46] Trotter WR Belyavin G Waddams A. Precipitating and complement-fixing antibodies in Hashimoto's disease. Proc R Soc Med. (1957) 50:961–2. 13494495

[47] Ludwig RJ Vanhoorelbeke K Leypoldt F Kaya Z Bieber K McLachlan SM. Mechanisms of autoantibody-induced pathology. Front Immunol. (2017) 8:603. 10.3389/fimmu.2017.00603 28620373 PMC5449453

[48] Karanikas G Schuetz M Wahl K Paul M Kontur S Pietschmann P. Relation of anti-TPO autoantibody titre and T-lymphocyte cytokine production patterns in Hashimoto's thyroiditis. Clin Endocrinol (Oxf). (2005) 63:191–6. 10.1111/j.1365-2265.2005.02324.x 16060913

[49] Chistiakov DA. Immunogenetics of Hashimoto's thyroiditis. J Autoimmune Dis. (2005) 2:1. 10.1186/1740-2557-2-1 15762980 PMC555850

[50] Nishikai M Homma M. Circulating autoantibody against human myoglobin in polymyositis. JAMA. (1977) 237:1842–4. 10.1001/jama.237.17.1842 576689

[51] Guilbert B Dighiero G Avrameas S. Naturally occurring antibodies against nine common antigens in human sera. Detection I, isolation and characterization. J Immunol. (1982) 128:2779–87. 6176652

[52] Madi A Bransburg-Zabary S Kenett DY Ben-Jacob E Cohen IR. The natural autoantibody repertoire in newborns and adults: a current overview. Adv Exp Med Biol. (2012) 750:198–212. 10.1007/978-1-4614-3461-0_15 22903676

[53] van Oosten BW Lai M Hodgkinson S Barkhof F Miller DH Moseley IF. Treatment of multiple sclerosis with the monoclonal anti-CD4 antibody cM-T412: results of a randomized, double-blind, placebo-controlled, MR-monitored phase II trial. Neurology. (1997) 49:351–7. 10.1212/WNL.49.2.351 9270561

[54] Jameson BA McDonnell JM Marini JC Korngold R. A rationally designed CD4 analogue inhibits experimental allergic encephalomyelitis. Nature. (1994) 368:744–6. 10.1038/368744a0 8152486

[55] Sewell AK. Why must T cells be cross-reactive? Nat Rev Immunol. (2012) 12:669–77. 10.1038/nri3279 22918468 PMC7097784

[56] Schmetzer O Lakin E Topal FA Preusse P Freier D Church MK. IL-24 is a common and specific autoantigen of IgE in patients with chronic spontaneous urticaria. J Allergy Clin Immunol. (2018) 142:876–82. 10.1016/j.jaci.2017.10.035 29208545

[57] Schmetzer O Moldenhauer G Riesenberg R Pires JR Schlag P Pezzutto A. Quality of recombinant protein determines the amount of autoreactivity detected against the tumor-associated epithelial cell adhesion molecule antigen: low frequency of antibodies against the natural protein. J Immunol. (2005) 174:942–52. 10.4049/jimmunol.174.2.942 15634917

[58] Shimizu F Schaller KL Owens GP Cotleur AC Kellner D Takeshita Y. Glucose-regulated protein 78 autoantibody associates with blood-brain barrier disruption in neuromyelitis optica. Sic Transl Med. (2017) 9:eaai9111. 10.1126/scitranslmed.aai9111 28679661 PMC5585784

[59] Crane JM Lam C Rossi A Gupta T Bennett JL Verkman AS. Binding affinity and specificity of neuromyelitis optica autoantibodies to aquaporin-4 M1/M23 isoforms and orthogonal arrays. J Biol Chem. (2011) 286:16516–24. 10.1074/jbc.M111.227298 21454592 PMC3091256

[60] Kang H Cao S Chen T Jiang Z Liu Z Li Z. The poor recovery of neuromyelitis optica spectrum disorder is associated with a lower level of CXCL12 in the human brain. J Neuroimmunol. (2015) 289:56–61. 10.1016/j.jneuroim.2015.10.005 26616871

[61] Satoh J Tabunoki H Yamamura T Arima K Konno H. Human astrocytes express aquaporin-1 and aquaporin-4 *in vitro* and *in vivo*. Neuropathology. (2007) 27:245–56. 10.1111/j.1440-1789.2007.00774.x 17645239

[62] Sinmaz N Amatoury M Merheb V Ramanathan S Dale RC Brilot F. Autoantibodies in movement and psychiatric disorders: updated concepts in detection methods, pathogenicity, and CNS entry. Ann N Y Acad Sci. (2015) 1351:22–38. 10.1111/nyas.12764 26083906

[63] Cree BAC Bennett JL Kim HJ Weinshenker BG Pittock SJ Wingerchuk DM. investigators, Inebilizumab for the treatment of neuromyelitis optica spectrum disorder (N-MOmentum): a double-blind, randomised placebo-controlled phase 2/3 trial. Lancet. (2019) 394:1352–63. 10.1016/S0140-6736(19)31817-3 31495497

[64] Bennett JL O'Connor KC Bar-Or A Zamvil SS Hemmer B Tedder TF. B lymphocytes in neuromyelitis optica. Neurol Neuroimmunol Neuroinflamm. (2015) 2:e104. 10.1212/NXI.0000000000000104 25977932 PMC4426682

[65] Ferraro AJ Drayson MT Savage CO MacLennan IC. Levels of autoantibodies, unlike antibodies to all extrinsic antigen groups, fall following B cell depletion with Rituximab. Eur J Immunol. (2008) 38:292–8. 10.1002/eji.200737557 18085668

[66] Cuadrado MJ Calatayud I Urquizu-Padilla M Wijetilleka S Kiani-Alikhan S Karim MY. Immunoglobulin abnormalities are frequent in patients with lupus nephritis. BMC Rheumatol. (2019) 3:30. 10.1186/s41927-019-0079-2 31453435 PMC6702722

[67] Nocturne G Mariette X. B cells in the pathogenesis of primary Sjogren syndrome. Nat Rev Rheumatol. (2018) 14:133–45. 10.1038/nrrheum.2018.1 29416129

[68] Skanse B Nilsson SB. Hypergammaglobulinemia and thyroid antibodies. Acta Med Scand. (1962) 172:505–12. 10.1111/j.0954-6820.1962.tb07185.x 13977863

[69] Dayan CM Daniels GH. Chronic autoimmune thyroiditis. N Engl J Med. (1996) 335:99–107. 10.1056/NEJM199607113350206 8649497

[70] Morais SA Vilas-Boas A Isenberg DA. B-cell survival factors in autoimmune rheumatic disorders. Ther Adv Musculoskelet Dis. (2015) 7:122–51. 10.1177/1759720X15586782 26288664 PMC4530383

