# Supplementary material for: Anti-aquaporin 4 IgG Is Not Associated With Any Clinical Disease Characteristics in Neuromyelitis Optica Spectrum Disorder
Source: Front Neurol. 2021 Mar 12;12:635419. doi: 10.3389/fneur.2021.635419 (PMC7994757; doi:10.3389/fneur.2021.635419)
Supplement: Supplementary file 1 [file Data_Sheet_1.PDF]

SUPPLEMENTAL MATERIAL

Figure S1

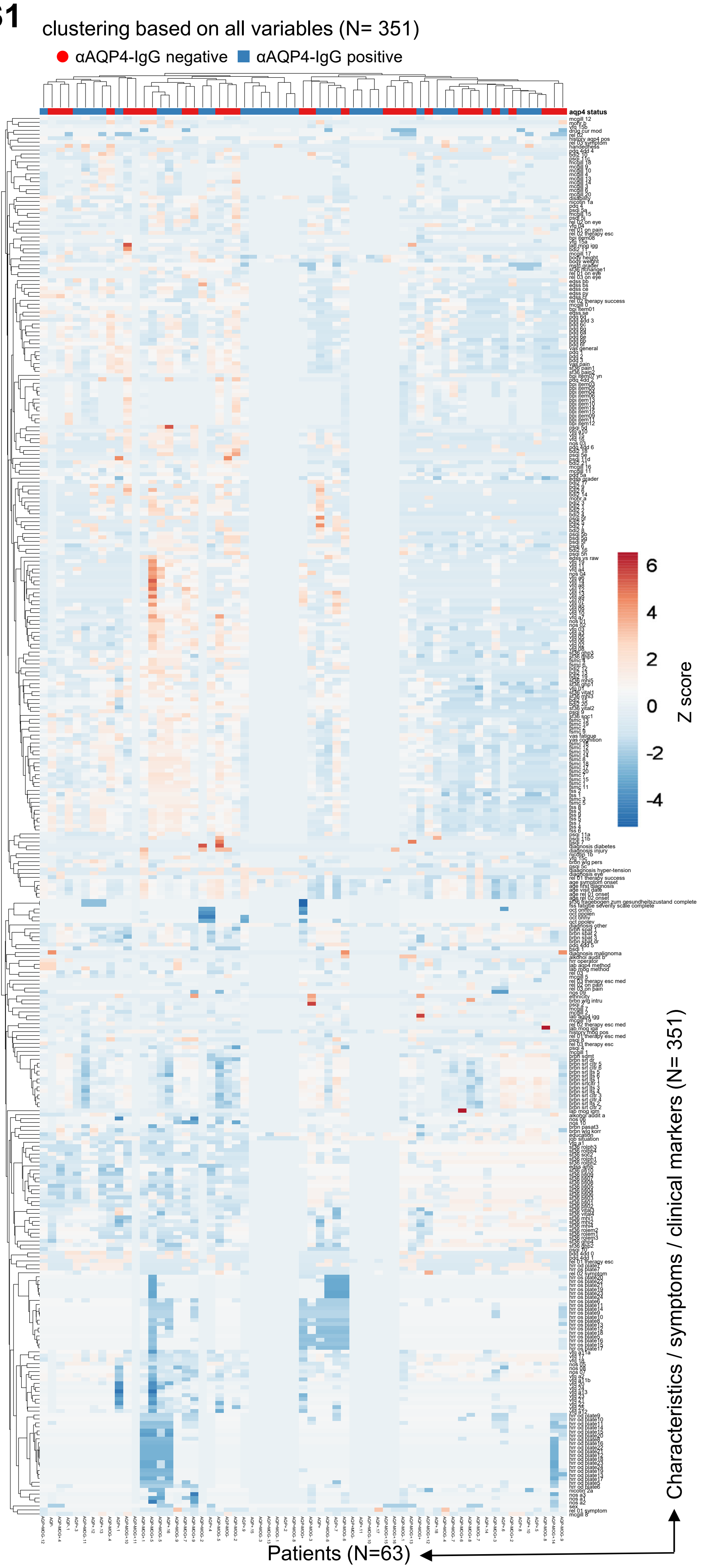
clustering based on all variables (N= 351)
αAQP4-IgG negative
αAQP4-IgG positive
aqp4 status
6
4
2
0
-2
-4
Z score
Patients (N=63)
Characteristics / symptoms / clinical markers (N= 351)

Figure S1. The anti-aquaporin 4 (AQP4)-IgG status does not define a subgroup that would be detectable in heatmap-based clustering analyses.

The same data sets as in Figure 1B and 2B were used to perform calculations based on phylogenetic clustering analyses using a heatmap, but all variables were named on the Y-axis.

Patients with Neuromyelitis optica spectrum disorder (NMOSD; N=63; X-axis) were clustered in columns using correlation distance and average linkage based on up to 351 symptoms and clinical markers (in rows, presented on the Y-axis). Unit variance scaling is applied to the symptoms and the clinical markers. On top of the rows, anti-AQP4 status is shown.

As before, no difference based on anti-AQP4-IgG status were identifiable.

Figure S2

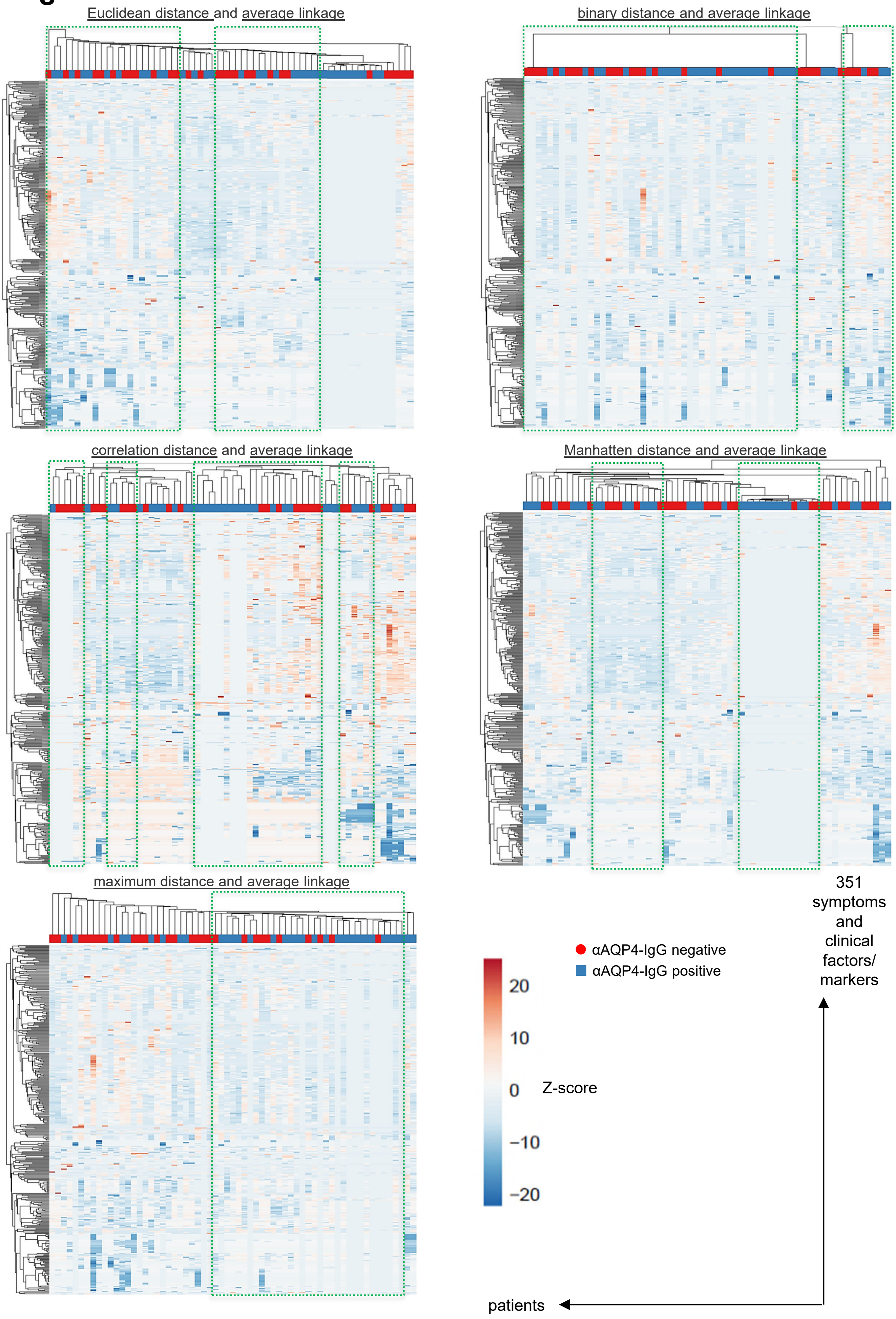
Euclidean distance and average linkage
binary distance and average linkage
correlation distance and average linkage
Manhatten distance and average linkage
maximum distance and average linkage
αAQP4-IgG negative
αAQP4-IgG positive
20
10
0
Z-score
−10
−20
351 symptoms and clinical factors/ markers
patients

Figure S2. The anti-aquaporin 4 (AQP4)-IgG status does not define a subgroup that would be detectable in heatmap-based clustering analyses independent of clustering method.

The same data sets as in Figure 1B, 2B and S1 were used to perform calculations based on phylogenetic clustering analyses using a heatmap.

Patients with Neuromyelitis optica spectrum disorder (NMOSD; N=63; X-axis) were clustered in columns using average linkage based on up to 351 symptoms and clinical markers (in rows, presented on the Y-axis), but five different distance settings (Euclidean, binary, correlation, Manhattan and maximum distance) were tested. Unit variance scaling is again applied to the symptoms and the clinical markers. On top of the rows, anti-AQP4 status is shown.

As before, no difference based on anti-AQP4-IgG status were identifiable.

Figure S3

Clustering distance	Clustering method	# of main clinical clusters
correlation distance	Ward (unsquared distances) linkage	3
	Ward linkage	7
	McQuitty linkage	56
	complete linkage	22
	single linkage	58
binary distance	average linkage	4
Manhattan distance		27
maximum distance		29
Euclidean distance		27
correlation distance		52

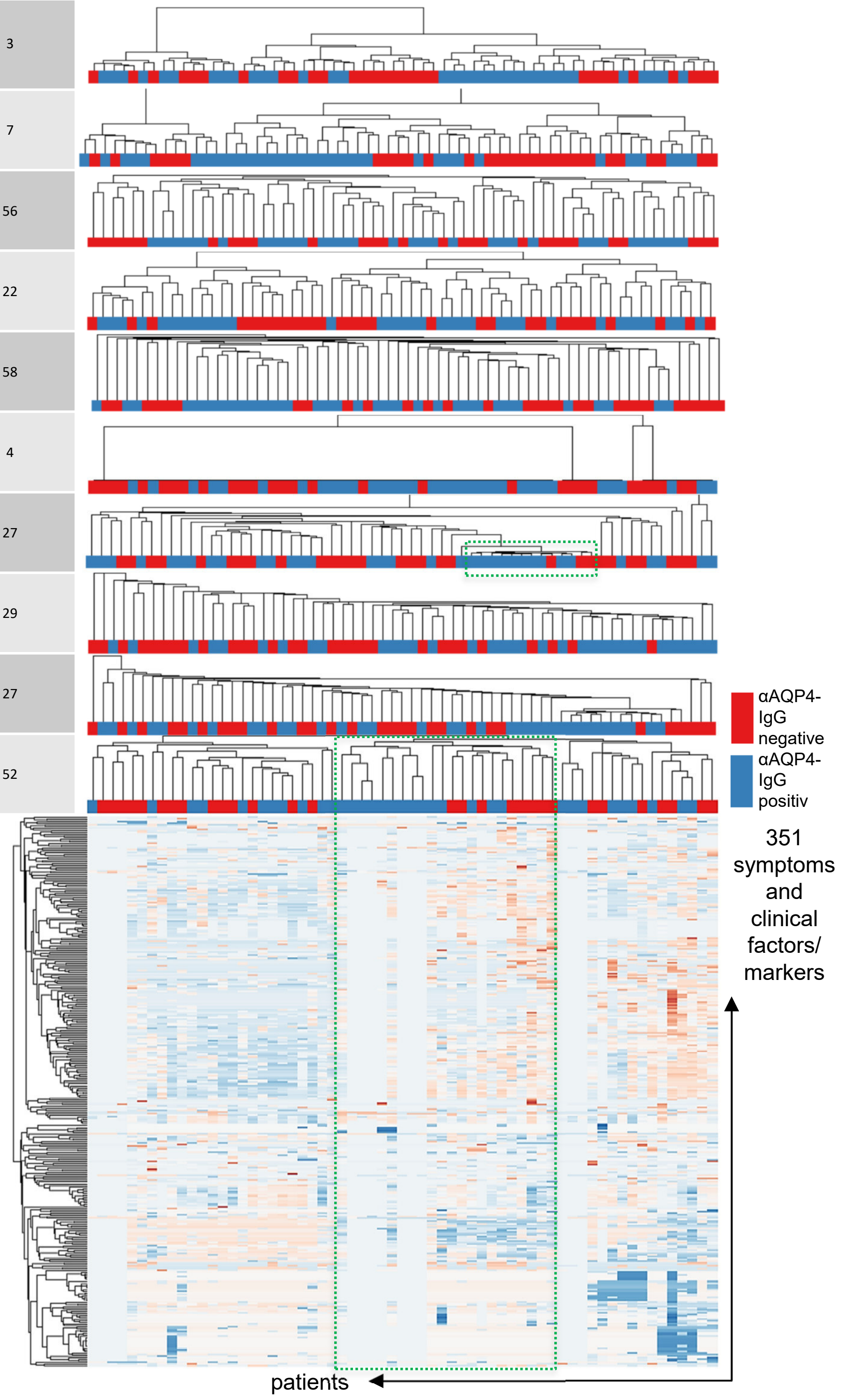

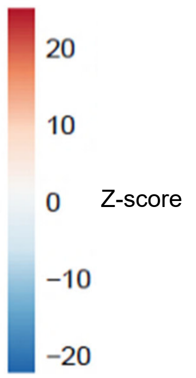

Figure S3. The anti-aquaporin 4 (AQP4)-IgG status does not define a subgroup that would be detectable in heatmap-based clustering analyses independent of clustering method.

We used the same data as in Fig S2, but in addition to different distance settings, we now also tested different linkage settings and show the respective cladograms on top of a heatmap, generated with correlation distance and average linkage settings. We again used our full set of 351 symptoms and clinical markers (in rows, presented on the Y-axis) and clustered our patients with Neuromyelitis optica spectrum disorder (NMOSD; N=63; X-axis).

Below each cladogram, anti-AQP4 status is shown. As before, no difference based on anti-AQP4-IgG status were identifiable.

Figure S4

Clustering distance	Clustering method	# of main clinical clusters
correlation distance	Ward (unsquared distances) linkage	3
	Ward linkage	6
	McQuitty linkage	57
	complete linkage	21
	single linkage	61
binary distance	average linkage	2
Canberra distance		not possible
Manhattan distance		40
maximum distance		19
Euclidean distance		52
correlation distance		40

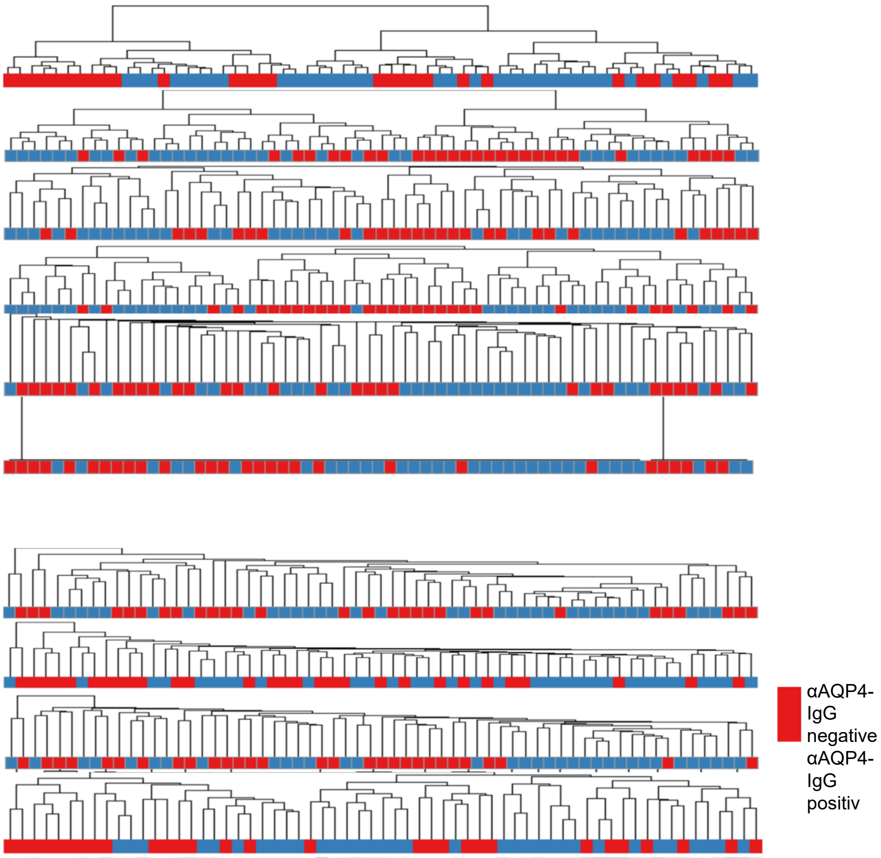

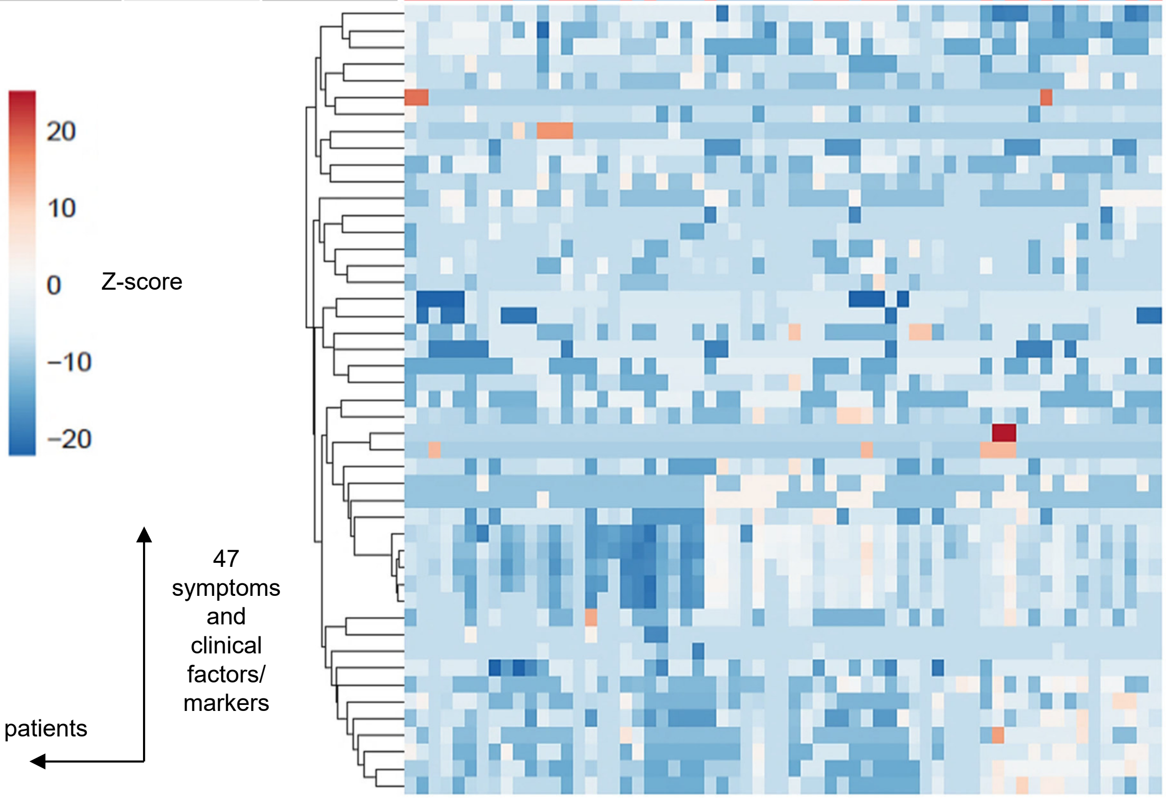

Figure S4. The anti-aquaporin 4 (AQP4)-IgG status does not define a subgroup that would be detectable in heatmap-based clustering analyses using a reduced number of variables and independent of clustering method.

We used a reduced data set of 47 key variables, which was generated by removing the more extensive clinical score details that deeply investigated single symptom areas (such as eye, psyche, detailed characteristics of pain etc.). These remaining 47 variables contained the most important measurements about disease severity, relapse rate, disability, EDSS score etc.

As in Fig S3 we tested again different distance and different linkage settings, the respective cladograms are shown on top of a heatmap, generated with correlation distance and average linkage settings based on the 47 variables (in rows, presented on the Y-axis).

Below each cladogram, anti-AQP4 status is shown. As before, no clear clustering based on anti-AQP4-IgG status were identifiable.

Figure S5

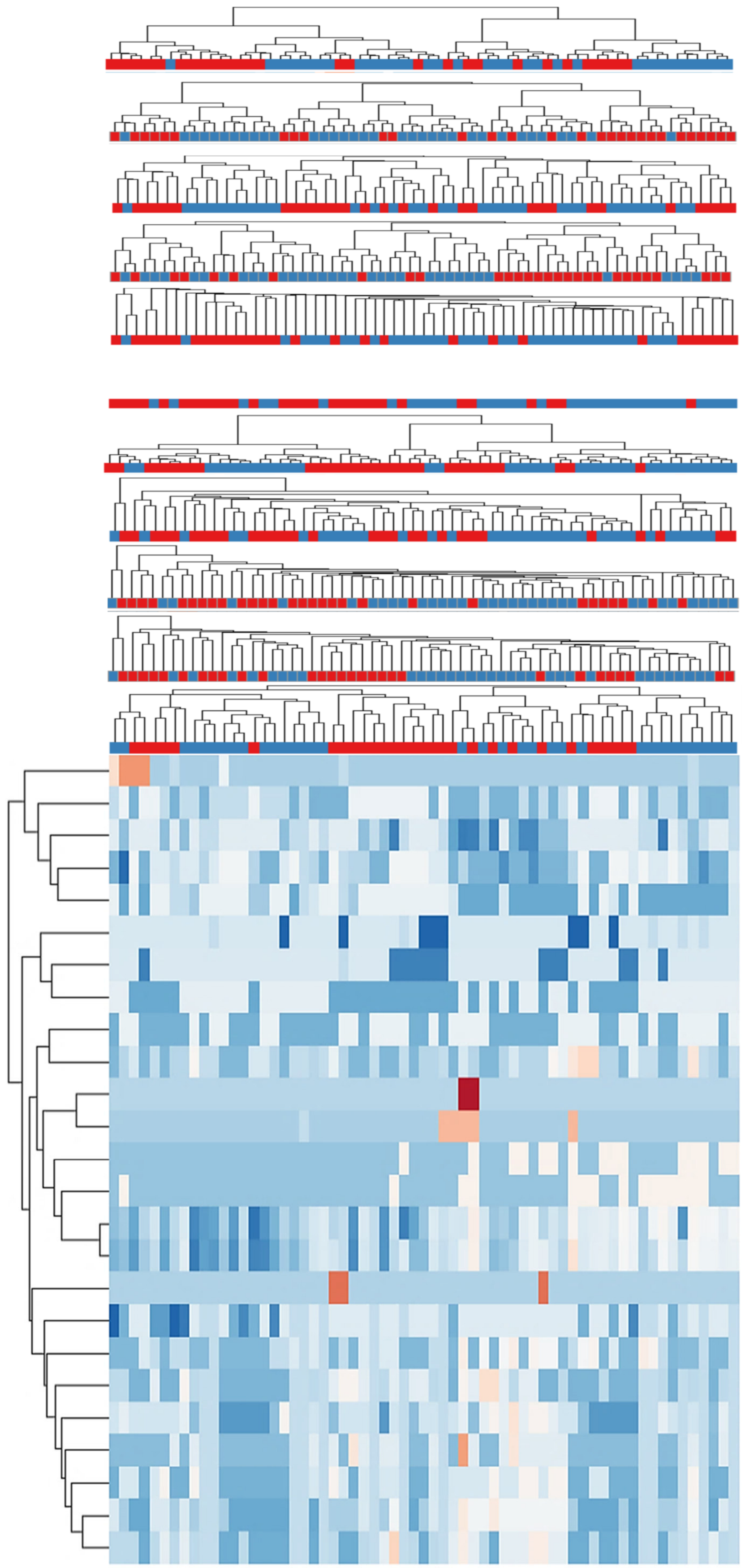

Clustering distance	Clustering method	# of main clinical clusters
correlation distance	Ward (unsquared distances) linkage	3
	Ward linkage	6
	McQuitty linkage	57
	complete linkage	21
	single linkage	61
binary distance	average linkage	2
Canberra distance		
Manhattan distance		40
maximum distance		19
Euclidean distance		52
correlation distance		40

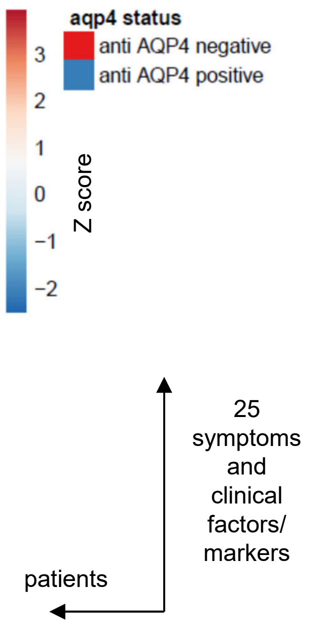

Figure S5. The anti-aquaporin 4 (AQP4)-IgG status does not define a subgroup that would be detectable in heatmap-based clustering analyses using a reduced number of variables and independent of clustering method.

We used a further reduced data set of 25 key variables as in Fig. S4 by removing variables dealing with the relapses of the patients. These remaining 25 variables therefore contained the most important measurements about disease severity, disability, EDSS score etc. at the first clinical visit, the baseline status of the patients.

As in Fig S4 we tested again different distance and different linkage settings, the respective cladograms are again shown on top of a heatmap, generated with correlation distance and average linkage settings based on the 25 variables (in rows, presented on the Y-axis).

Below each cladogram, anti-AQP4 status is shown. As before, no clear clustering based on anti-AQP4-IgG status were identifiable.

Figure S6

Clustering distance	Clustering method	# of main clinical clusters
correlation distance	Ward (unsquared distances) linkage	3
correlation distance	Ward linkage	6
correlation distance	McQuitty linkage	57
correlation distance	complete linkage	21
correlation distance	single linkage	61
binary distance	average linkage	n.d.
Canberra distance	average linkage	2
Manhattan distance	average linkage	40
maximum distance	average linkage	19
Euclidean distance	average linkage	52
correlation distance	average linkage	40

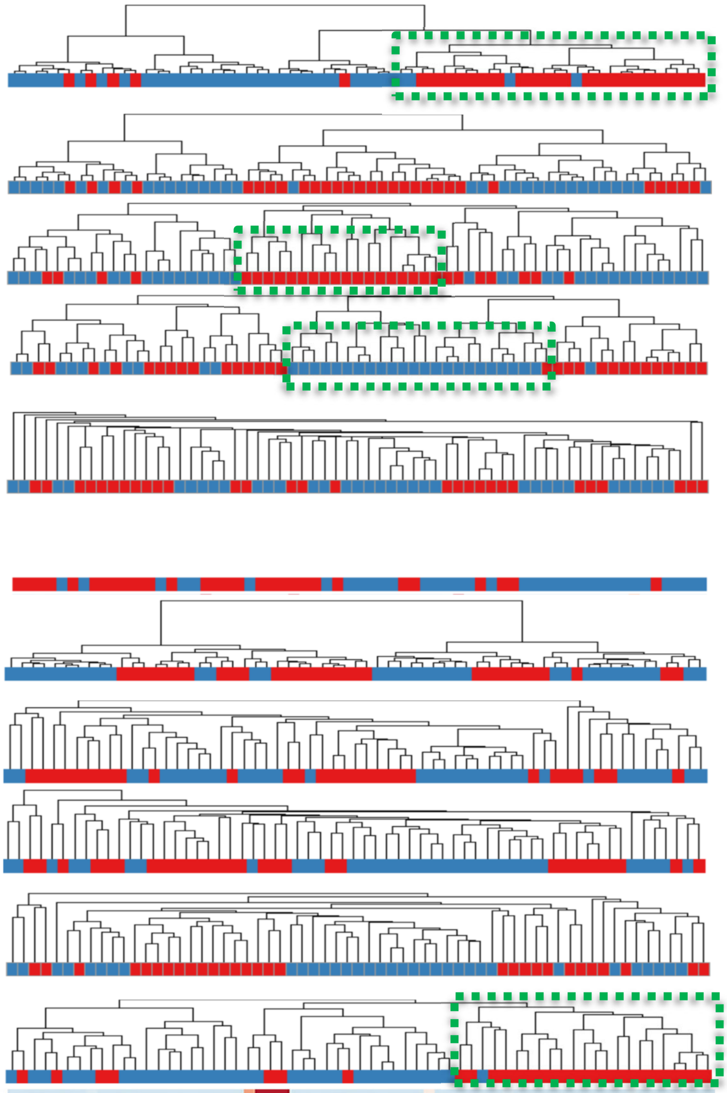

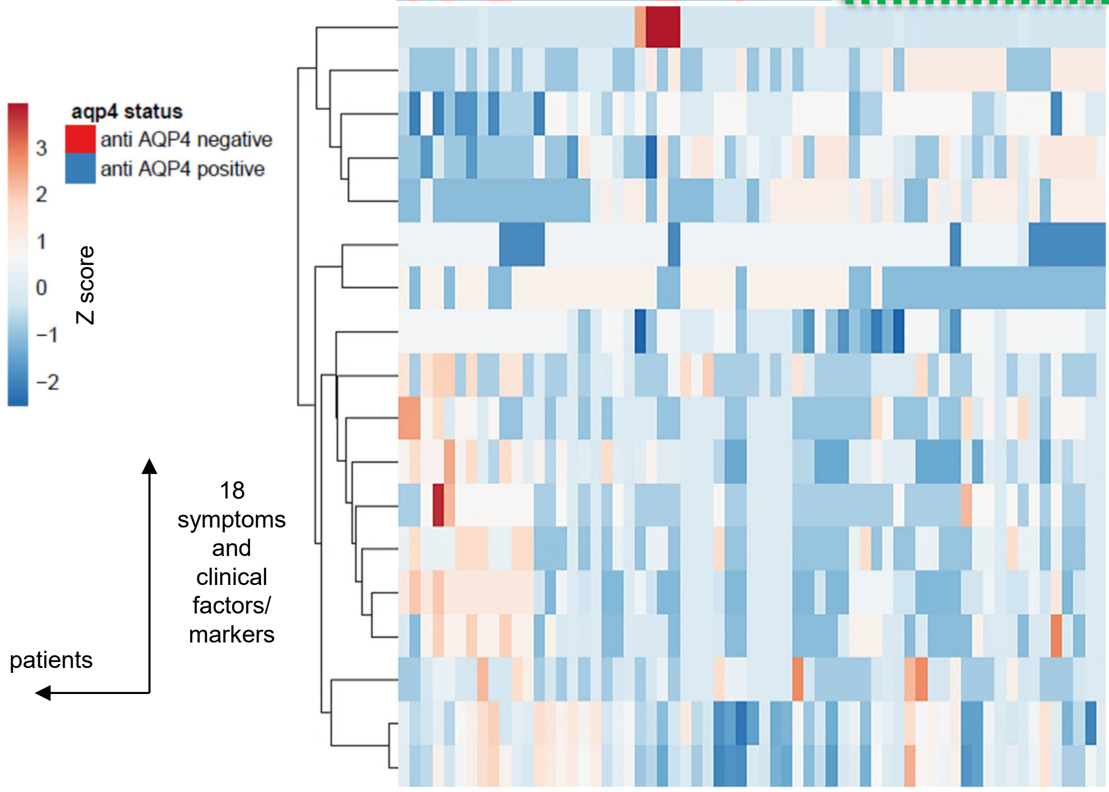

Figure S6. The anti-aquaporin 4 (AQP4)-IgG status does not define a subgroup that would be detectable in heatmap-based clustering analyses using a minimal number of variables and independent of clustering method.

We used a further reduced data set of 18 key variables as in Fig. S5 by further removing variables dealing with additional other diagnosis of the patients. These remaining 18 variables therefore contained only some measurements about disease severity, disability, EDSS score etc. at the first clinical visit only (the baseline status) of the patients and consist mainly of biographic information.

As in Fig S5 we tested again different distance and different linkage settings, the respective cladograms are again shown on top of a heatmap, generated with correlation distance and average linkage settings based on the 18 variables (in rows, presented on the Y-axis).

Below each cladogram, anti-AQP4 status is shown. As before, no clear clustering based on anti-AQP4-IgG status were identifiable. However, a certain degree of subgroups correlating with anti-AQP4 status are formed now with certain settings. Such as in the lowest cladogram where anti-AQP4 negative patients form a subgroup on the right side of the heatmap, the positives on the left side.

However, this clustering is mainly based on biographic variables and the previous history of anti-MOG-IgG.

Those patients with a higher education level, a better job situation such as full-time employment and with previously singular positive reports of anti-MOG-IgG are clustered on the right, the anti-AQP4 negative side. Therefore, these results seem to be an artificial finding due to a selection bias which got impact if the number of variables, especially disease relevant markers, is reduced too far.

The more severely ill patients (here N=12) with high EDSS subscores are clustered on the very left and have still mixed anti-AQP4 status.

Figure S7

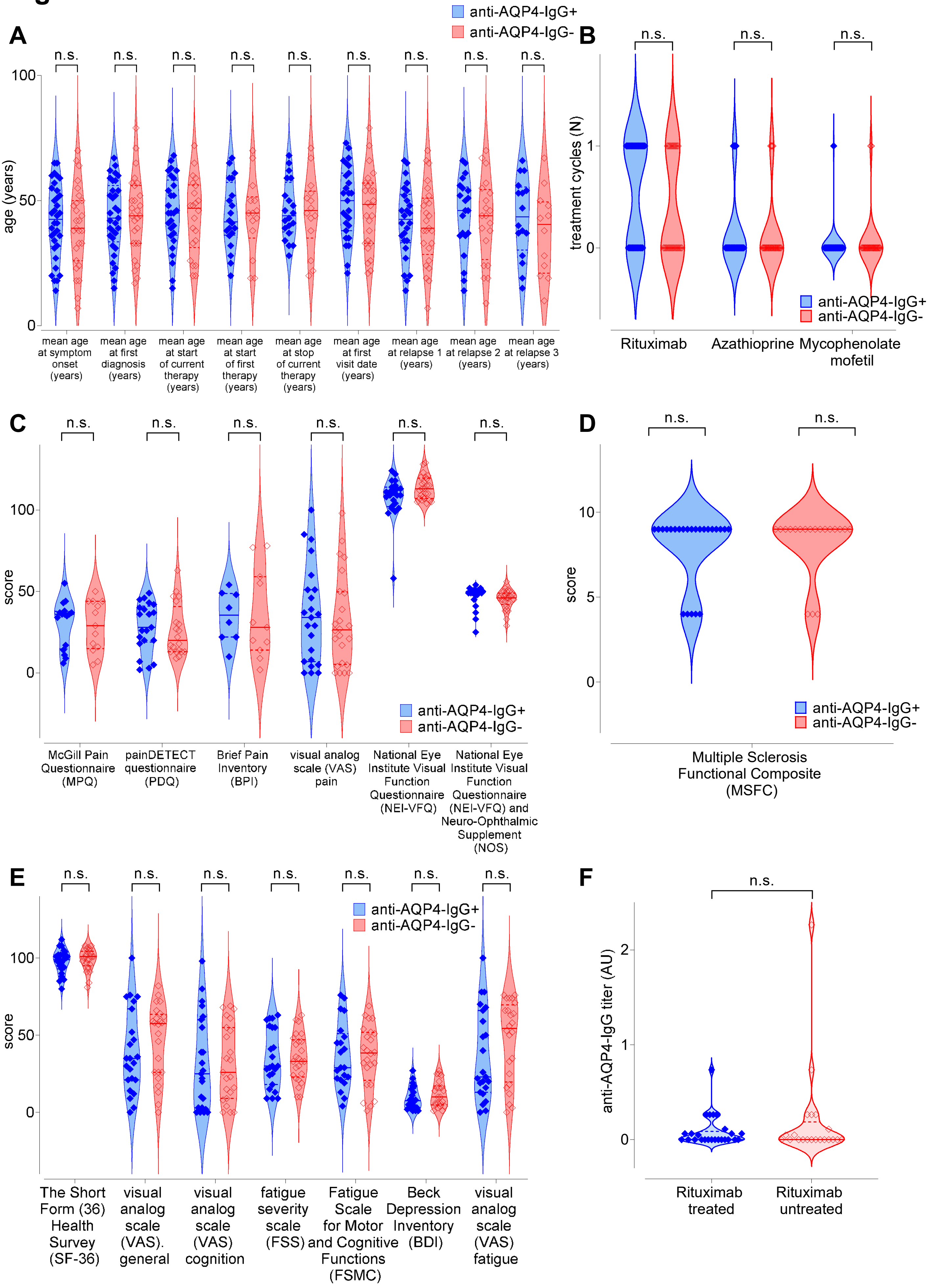

A
anti-AQP4-IgG+
anti-AQP4-IgG-
n.s.
age (years)
100
50
0
mean age at symptom onset (years)
mean age at first diagnosis (years)
mean age at start of current therapy (years)
mean age at start of first therapy (years)
mean age at stop of current therapy (years)
mean age at first visit date (years)
mean age at relapse 1 (years)
mean age at relapse 2 (years)
mean age at relapse 3 (years)
B
treatment cycles (N)
1
0
Rituximab
Azathioprine
Mycophenolate mofetil
C
score
100
50
0
McGill Pain Questionnaire (MPQ)
painDETECT questionnaire (PDQ)
Brief Pain Inventory (BPI)
visual analog scale (VAS) pain
National Eye Institute Visual Function Questionnaire (NEI-VFQ)
National Eye Institute Visual Function Questionnaire (NEI-VFQ) and Neuro-Ophthalmic Supplement (NOS)
D
score
10
5
0
Multiple Sclerosis Functional Composite (MSFC)
E
score
100
50
0
The Short Form (36) Health Survey (SF-36)
visual analog scale (VAS). general
visual analog scale (VAS) cognition
fatigue severity scale (FSS)
Fatigue Scale for Motor and Cognitive Functions (FSMC)
Beck Depression Inventory (BDI)
visual analog scale (VAS) fatigue
F
anti-AQP4-IgG titer (AU)
2
1
0
Rituximab treated
Rituximab untreated

Figure S7. A positive anti-aquaporin 4 (AQP4)-IgG status is not associated with disease history, age at relapses, treatment plans, nor with pain or with visual function, functional, fatigue and psychometric score results. Rituximab treatment does not alter anti-AQP4-IgG titer.

Anti-aquaporin 4 (AQP4)-IgG status did not correlate with age of symptom onset, diagnosis, treatment trial start and stop ages nor did it correlate with the age at the first three relapses (A). Anti-aquaporin 4 (AQP4)-IgG status also did not correlate with the usage of immunomodulatory therapy (B).

There were no detectable differences related to the anti-aquaporin 4 (AQP4)-IgG status in scores assesing pain and visual function (C), nor in the major functional score MSFC testing disability (D) or in general health and psychometric scores (E).

The Anti-aquaporin 4 (AQP4)-IgG titer (determined with the second and third visit) of the patients did also not change after patients have been treated with the B cell targeting drug rituximab during their initial, first major episode of disease activity (F). The second and third visit was approx. one and two years after the baseline visit and patients had mostly received a full treatment regime with rituximab for six months or longer. The majority of patients had a clear clinical benefit from the treatment, which also did not correlate with anti-aquaporin 4 (AQP4)-IgG status (not shown).

Figure S8

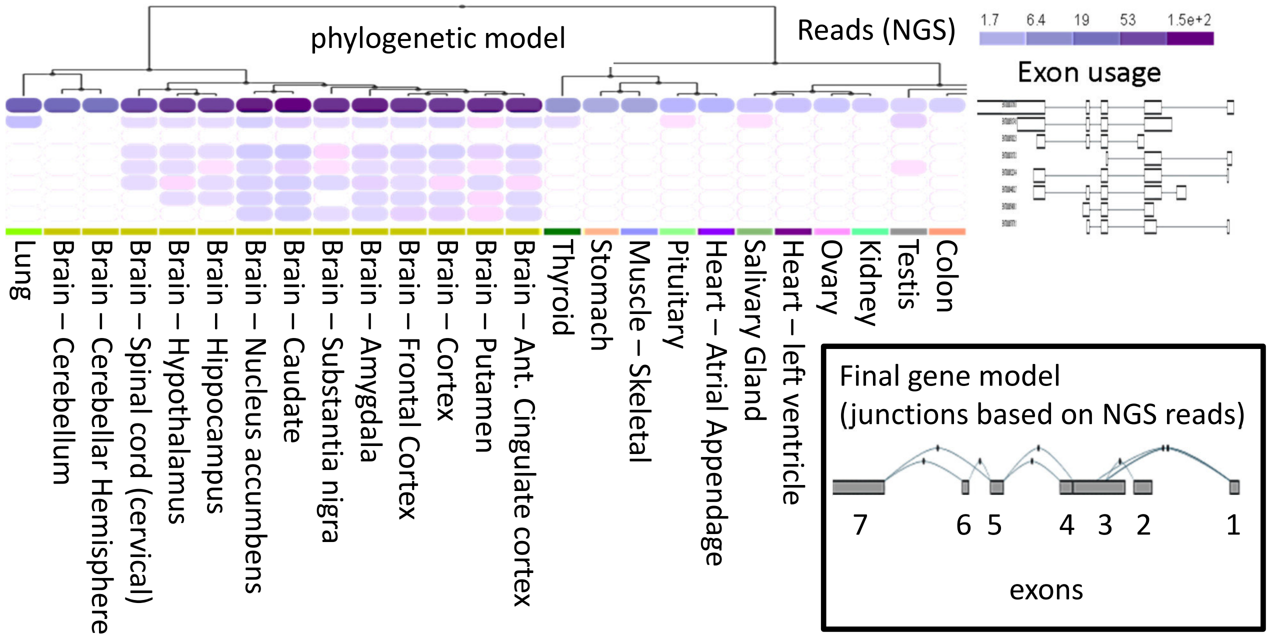

Figure S8. Expression data for all AQP4 isoforms shows a broad expression of the main transcript outside the CNS.

There are eight known transcripts of AQP4 which represent different isoforms on the protein level. Here the expression strength of the all eight manuscripts is shown, the first one is highest expressed also in lung (higher as in the CNS on protein level due to longer half-life, not shown), the whole CNS including SC as well as in the thyroid, GI system and other organs. There are CNS specific transcripts 4-8 which have a different exon usage and should lead to different encoding, likely junk proteins (only transcripts 5 and 6 could be functional). Several antisense RNAs are expressed outside of the main gene (not shown). The final model of exon usage is shown in the insert on the lower right. Exon expression from GTEx Analysis Release V8 (dbGaP Accession phs000424.v8.p2) is shown based on expression data of AQP4: ENSG00000171885.13 from HGNC. (https://www.gtexportal.org/home/gene/AQP4; Accession: HGNC: 637).

Abbreviations: GTEx: Genotype-Tissue Expression; dbGaP: database of Genotypes and Phenotypes.

Abbreviation: NGS: next generation sequencing

Table S1. A list of all 1232 variables assessed for this study with explanation is shown.

A maximum of 1232 variables was collected for each patient and each of their visits. However, only 351 of these variables could finally be used in our study.

We used any variable which resulted in a numeric, non-descriptive value or those which could be turned into a numeric result and for which we had a reasonable number of tested subjects. One limitation was that, due to time and other reasons, sometimes only a fraction of the tests could be performed on each patient. More extensive tests could only be carried out for a few patients and could therefore not be included in our analysis.

Table S1

variable code	Explanation
record_id	basal ID information-NMO patient ID (NMO-XXXX)
redcap_event_name	basal ID information-Event name
redcap_survey_identifier	basal ID information-Survey Identifier
id_master	basal ID information-Master ID
kknms_id	basal ID information-KKNMS ID
croctino_id	basal ID information-CROCTINO ID
noir_id	basal ID information-CIRCLES ID
studien_ids_complete	basal ID information-Complete?
birth_date	general patient characteristic-base data-Date of birth
sex	general patient characteristic-base data-gender
body_height	general patient characteristic-base data-height
body_weight	general patient characteristic-base data-body weight
handedness	general patient characteristic-base data-Writing hand
education	general patient characteristic-base data-Highest degree
job_situation	general patient characteristic-base data-Gainful employment
disability	general patient characteristic-base data-Degree of disability
ethnicity	general patient characteristic-base data-Ethnicity
ethnicity_other	general patient characteristic-base data-Ethnicity (other)
mother_tongue	general patient characteristic-base data-native language
stammdaten_complete	general patient characteristic-base data-Complete?
incl_age	general patient characteristic-inclusion criteria-Age between 18 and 70 years
incl_nmosd	general patient characteristic-inclusion criteria-Presence of a disease from the Neuromyelitis Optica spectrum (see below)
excl_diseases	general patient characteristic-inclusion criteria-Are there any clinically relevant comorbidities that the investigator believes would prevent participation in the study? (e.g. arterial hypertension that is not controlled by medication, severe diabetes mellitus, alcohol or drug abuse, chronic infectious diseases such as HIV or hepatitis)
excl_mri	general patient characteristic-inclusion criteria-Are there any contraindications to having an MRI? (e.g. pacemaker, cochlear implant, metal in or on the body, contrast medium intolerance)
excl_consent_able	general patient characteristic-inclusion criteria-Is the patient restricted by medical / psychiatric or other causes from understanding the patient information, giving informed consent, following the rules of the protocol and completing the study?
excl_pregnancy	general patient characteristic-inclusion criteria-There is a pregnancy
einschlusskriterien_und_ausschlusskriterien_complete	general patient characteristic-inclusion criteria-Complete?
symptom_onset	patient history-diagnostic criteria-Date of first manifestation
first_diagnosis	patient history-diagnostic criteria-Date of initial diagnosis
diagnosis_inclusion	patient history-diagnostic criteria-Inclusion diagnosis
diagnosis_inclusion_other	patient history-diagnostic criteria-If other, please state
history_aqp4	patient history-diagnostic criteria-Have AQP4 antibodies ever been tested?
history_aqp4_pos	patient history-diagnostic criteria-Was the AQP4 antibody test ever positive?
history_mog	patient history-diagnostic criteria-Have MOG antibodies ever been tested?
history_mog_pos	patient history-diagnostic criteria-Has the MOG antibody test ever been positive?
csf	patient history-diagnostic criteria-Has a liquor aspiration ever been performed at the Charité?
csf_date	patient history-diagnostic criteria-Date of the CSF puncture
csf_consent	patient history-diagnostic criteria-Has the consent for the scientific use of CSF been given?
diagnose_complete	patient history-diagnostic criteria-Complete?
criteria_nmo2015	patient history-diagnostic criteria-Is there an NMOSD with AQP4-IgG according to Wingerchuk 2015?
criteria_nmo2015_aqp_negdd	patient history-diagnostic criteria-Is there an NMOSD according to Wingerchuk 2015 without AQP4-IgG or with an unknown AQP4-IgG status?
criteria_mog	patient history-diagnostic criteria-Is there a MOG-Ab positive optic spinal neuropathy?
diagnosekriterien_complete	patient history-diagnostic criteria-Complete?
diagnosis_thyroiditis	patient history-other diseases-Autoimmune thyroiditis
diagnosis_sle	patient history-other diseases-SLE
diagnosis_myasthenia	patient history-other diseases-Myasthenia gravis
diagnosis_sjoegren	patient history-other diseases-Sjogren's Syndrome
diagnosis_paraneoplastic	patient history-other diseases-Malignancy with paraneoplastic symptoms
diagnosis_immune_other	patient history-other diseases-Others (please specify)
diagnosis_immune_other_details	patient history-other diseases-If other, then what?
diagnosis_eye	patient history-other diseases-Are there any known eye conditions that could affect the neurovisual exam?
diagnosis_eye_od_details	patient history-other diseases-If so, which ones on the right eye (OD)
diagnosis_eye_os_details	patient history-other diseases-If so, which ones on the left eye (OS)
diagnosis_diabetes	patient history-other diseases-Diabetes mellitus
diaagnosis_hypertension	patient history-other diseases-Arterial hypertension
diagnosis_malignoma	patient history-other diseases-Malignancy (without paraneoplastic symptoms)
diagnosis_injury	patient history-other diseases-Spinal or head injuries
diagnosis_other	patient history-other diseases-Are there any other diagnosed neurological, psychiatric, internal or orthopedic diseases?
diagnosis_other_details	patient history-other diseases-If yes, which?
vorerkrankungen_complete	patient history-other diseases-Complete?
drug_cur_mod	therapeutic drug history-Is the patient currently taking an immunomodulatory or immunosuppressive drug?
drug_cur_mod_name	therapeutic drug history-drug
drug_cur_mod_start	therapeutic drug history-Start therapy
drug_cur_mod_dosis	therapeutic drug history-dosage
drug_cur_mod_freq	therapeutic drug history-frequency
drug_cur_mod_app	therapeutic drug history-Application route
drug_cur_psycho	therapeutic drug history-Is the patient currently receiving psychotherapy?
drug_cur_neuro	therapeutic drug history-Is the patient currently taking other medications with neurological / psychiatric indications on a regular basis?
drug_cur_neuro_comments	therapeutic drug history-If yes, which?
drug_cur_other	therapeutic drug history-Is the patient currently taking any other medication on a regular basis?
drug_cur_other_comment	therapeutic drug history-If yes, which?
aktuelle_medikation_und_therapie_complete	therapeutic drug history-Complete?
drug_hist_mod	therapeutic drug history-Did the patient receive any further immunomodulatory therapies before the current therapy?
drug_hist_mod_name	therapeutic drug history-drug
drug_hist_mod_start	therapeutic drug history-Beginning
drug_hist_mod_stop	therapeutic drug history-The End
drug_hist_mod_freq	therapeutic drug history-frequency
drug_hist_mod_app	therapeutic drug history-application
drug_hist_mod_comment	therapeutic drug history-Remarks
drug_hist_mod_more	therapeutic drug history-Has the patient received any other immunomodulatory or immunosuppressive therapies beforehand?
drug_hist_mod_more_comments	therapeutic drug history-If yes, which
vorherige_immunmodulatorische_oder_suppressive_med_complete	therapeutic drug history-Complete?
visit_date	clinical examination-Date of the visit
visit_comments	clinical examination-Remarks
visit_complete	clinical examination-Complete?
anamn_rr_systolic	clinical examination- Systolic blood pressure
anamn_rr_diastolic	clinical examination-Diastolic blood pressure
anamn_hf	clinical examination- Heart rate
anamnese_und_vitalparameter_complete	clinical examination-vital signs-Complete?
rel_01_symptom	documentation of relapse 1-symptom

Table S1

variable code	Explanation
rel_01_description	documentation of relapse 1-Symptom description
rel_01_on_eye	documentation of relapse 1-Optic neuritis - eye
rel_01_on_pain	documentation of relapse 1-Optic neuritis - pain
rel_01_onset	documentation of relapse 1-Onset of symptoms
rel_01_therapy	documentation of relapse 1-therapy
rel_01_therapy_esc	documentation of relapse 1-Therapy escalation
rel_01_therapy_esc_med	documentation of relapse 1-Therapy escalation with
rel_01_therapy_success	documentation of relapse 1-Therapy success
rel_01_comments	documentation of relapse 1-Remarks
rel_02	documentation of relapse 2-Has there been another surge?
rel_02_symptom	documentation of relapse 2-symptom
rel_02_description	documentation of relapse 2-Symptom description
rel_02_on_eye	documentation of relapse 2-Optic neuritis - eye
rel_02_on_pain	documentation of relapse 2-Optic neuritis - pain
rel_02_onset	documentation of relapse 2-Onset of symptoms
rel_02_therapy	documentation of relapse 2-therapy
rel_02_therapy_esc	documentation of relapse 2-Therapy escalation
rel_02_therapy_esc_med	documentation of relapse 2-Therapy escalation with
rel_02_therapy_success	documentation of relapse 2-Therapy success
rel_02_comments	documentation of relapse 2-Remarks
rel_03	documentation of relapse 3-Has there been another surge?
rel_03_symptom	documentation of relapse 3-symptom
rel_03_description	documentation of relapse 3-Symptom description
rel_03_on_eye	documentation of relapse 3-Optic neuritis - eye
rel_03_on_pain	documentation of relapse 3-Optic neuritis - pain
rel_03_onset	documentation of relapse 3-Onset of symptoms
rel_03_therapy	documentation of relapse 3-therapy
rel_03_therapy_esc	documentation of relapse 3-Therapy escalation
rel_03_therapy_esc_med	documentation of relapse 3-Therapy escalation with
rel_03_therapy_success	documentation of relapse 3-Therapy success
rel_03_comments	documentation of relapse 3-Remarks
rel_04	documentation of relapse 4-Has there been another surge?
rel_04_symptom	documentation of relapse 4-symptom
rel_04_description	documentation of relapse 4-Symptom description
rel_04_on_eye	documentation of relapse 4-Optic neuritis - eye
rel_04_on_pain	documentation of relapse 4-Optic neuritis - pain
rel_04_onset	documentation of relapse 4-Onset of symptoms
rel_04_therapy	documentation of relapse 4-therapy
rel_04_therapy_esc	documentation of relapse 4-Therapy escalation
rel_04_therapy_esc_med	documentation of relapse 4-Therapy escalation with
rel_04_therapy_success	documentation of relapse 4-Therapy success
rel_04_comments	documentation of relapse 4-Remarks
rel_05	documentation of relapse 5-Has there been another surge?
rel_05_symptom	documentation of relapse 5-symptom
rel_05_description	documentation of relapse 5-Symptom description
rel_05_on_eye	documentation of relapse 5-Optic neuritis - eye
rel_05_on_pain	documentation of relapse 5-Optic neuritis - pain
rel_05_onset	documentation of relapse 5-Onset of symptoms
rel_05_therapy	documentation of relapse 5-therapy
rel_05_therapy_esc	documentation of relapse 5-Therapy escalation
rel_05_therapy_esc_med	documentation of relapse 5-Therapy escalation with
rel_05_therapy_success	documentation of relapse 5-Therapy success
rel_05_comments	documentation of relapse 5-Remarks
rel_06	documentation of relapse 6-Has there been another surge?
rel_06_symptom	documentation of relapse 6-symptom
rel_06_description	documentation of relapse 6-Symptom description
rel_06_on_eye	documentation of relapse 6-Optic neuritis - eye
rel_06_on_pain	documentation of relapse 6-Optic neuritis - pain
rel_06_onset	documentation of relapse 6-Onset of symptoms
rel_06_therapy	documentation of relapse 6-therapy
rel_06_therapy_esc	documentation of relapse 6-Therapy escalation
rel_06_therapy_esc_med	documentation of relapse 6-Therapy escalation with
rel_06_therapy_success	documentation of relapse 6-Therapy success
rel_06_comments	documentation of relapse 6-Remarks
rel_07	documentation of relapse 7-Has there been another surge?
rel_07_symptom	documentation of relapse 7-symptom
rel_07_description	documentation of relapse 7-Symptom description
rel_07_on_eye	documentation of relapse 7-Optic neuritis - eye
rel_07_on_pain	documentation of relapse 7-Optic neuritis - pain
rel_07_onset	documentation of relapse 7-Onset of symptoms
rel_07_therapy	documentation of relapse 7-therapy
rel_07_therapy_esc	documentation of relapse 7-Therapy escalation
rel_07_therapy_esc_med	documentation of relapse 7-Therapy escalation with
rel_07_therapy_success	documentation of relapse 7-Therapy success
rel_07_comments	documentation of relapse 7-Remarks
rel_08	documentation of relapse 8-Has there been another surge?
rel_08_symptom	documentation of relapse 8-symptom
rel_08_description	documentation of relapse 8-Symptom description
rel_08_on_eye	documentation of relapse 8-Optic neuritis - eye
rel_08_on_pain	documentation of relapse 8-Optic neuritis - pain
rel_08_onset	documentation of relapse 8-Onset of symptoms
rel_08_therapy	documentation of relapse 8-therapy
rel_08_therapy_esc	documentation of relapse 8-Therapy escalation
rel_08_therapy_esc_med	documentation of relapse 8-Therapy escalation with
rel_08_therapy_success	documentation of relapse 8-Therapy success
rel_08_comments	documentation of relapse 8-Remarks
rel_09	documentation of relapse 9-Has there been another surge?
rel_09_symptom	documentation of relapse 9-symptom
rel_09_description	documentation of relapse 9-Symptom description
rel_09_on_eye	documentation of relapse 9-Optic neuritis - eye
rel_09_on_pain	documentation of relapse 9-Optic neuritis - pain
rel_09_onset	documentation of relapse 9-Onset of symptoms
rel_09_therapy	documentation of relapse 9-therapy
rel_09_therapy_esc	documentation of relapse 9-Therapy escalation
rel_09_therapy_esc_med	documentation of relapse 9-Therapy escalation with
rel_09_therapy_success	documentation of relapse 9-Therapy success
rel_09_comments	documentation of relapse 9-Remarks
rel_10	documentation of relapse 10-Has there been another surge?
rel_10_symptom	documentation of relapse 10-symptom
rel_10_description	documentation of relapse 10-Symptom description
rel_10_on_eye	documentation of relapse 10-Optic neuritis - eye
rel_10_on_pain	documentation of relapse 10-Optic neuritis - pain

Table S1

variable code	Explanation
rel_10_onset	documentation of relapse 10-Onset of symptoms
rel_10_therapy	documentation of relapse 10-therapy
rel_10_therapy_esc	documentation of relapse 10-Therapy escalation
rel_10_therapy_esc_med	documentation of relapse 10-Therapy escalation with
rel_10_therapy_success	documentation of relapse 10-Therapy success
rel_10_comments	documentation of relapse 10-Remarks
rel_11	documentation of relapse 11-Has there been another surge?
rel_11_symptom	documentation of relapse 11-symptom
rel_11_description	documentation of relapse 11-Symptom description
rel_11_on_eye	documentation of relapse 11-Optic neuritis - eye
rel_11_on_pain	documentation of relapse 11-Optic neuritis - pain
rel_11_onset	documentation of relapse 11-Onset of symptoms
rel_11_therapy	documentation of relapse 11-therapy
rel_11_therapy_esc	documentation of relapse 11-Therapy escalation
rel_11_therapy_esc_med	documentation of relapse 11-Therapy escalation with
rel_11_therapy_success	documentation of relapse 11-Therapy success
rel_11_comments	documentation of relapse 11-Remarks
rel_12	documentation of relapse 12-Has there been another surge?
rel_12_symptom	documentation of relapse 12-symptom
rel_12_description	documentation of relapse 12-Symptom description
rel_12_on_eye	documentation of relapse 12-Optic neuritis - eye
rel_12_on_pain	documentation of relapse 12-Optic neuritis - pain
rel_12_onset	documentation of relapse 12-Onset of symptoms
rel_12_therapy	documentation of relapse 12-therapy
rel_12_therapy_esc	documentation of relapse 12-Therapy escalation
rel_12_therapy_esc_med	documentation of relapse 12-Therapy escalation with
rel_12_therapy_success	documentation of relapse 12-Therapy success
rel_12_comments	documentation of relapse 12-Remarks
rel_13	documentation of relapse 13-Has there been another surge?
rel_13_symptom	documentation of relapse 13-symptom
rel_13_description	documentation of relapse 13-Symptom description
rel_13_on_eye	documentation of relapse 13-Optic neuritis - eye
rel_13_on_pain	documentation of relapse 13-Optic neuritis - pain
rel_13_onset	documentation of relapse 13-Onset of symptoms
rel_13_therapy	documentation of relapse 13-therapy
rel_13_therapy_esc	documentation of relapse 13-Therapy escalation
rel_13_therapy_esc_med	documentation of relapse 13-Therapy escalation with
rel_13_therapy_success	documentation of relapse 13-Therapy success
rel_13_comments	documentation of relapse 13-Remarks
rel_14	documentation of relapse 14-Has there been another surge?
rel_14_symptom	documentation of relapse 14-symptom
rel_14_description	documentation of relapse 14-Symptom description
rel_14_on_eye	documentation of relapse 14-Optic neuritis - eye
rel_14_on_pain	documentation of relapse 14-Optic neuritis - pain
rel_14_onset	documentation of relapse 14-Onset of symptoms
rel_14_therapy	documentation of relapse 14-therapy
rel_14_therapy_esc	documentation of relapse 14-Therapy escalation
rel_14_therapy_esc_med	documentation of relapse 14-Therapy escalation with
rel_14_therapy_success	documentation of relapse 14-Therapy success
rel_14_comments	documentation of relapse 14-Remarks
rel_15	documentation of relapse 15-Has there been another surge?
rel_15_symptom	documentation of relapse 15-symptom
rel_15_description	documentation of relapse 15-Symptom description
rel_15_on_eye	documentation of relapse 15-Optic neuritis - eye
rel_15_on_pain	documentation of relapse 15-Optic neuritis - pain
rel_15_onset	documentation of relapse 15-Onset of symptoms
rel_15_therapy	documentation of relapse 15-therapy
rel_15_therapy_esc	documentation of relapse 15-Therapy escalation
rel_15_therapy_esc_med	documentation of relapse 15-Therapy escalation with
rel_15_therapy_success	documentation of relapse 15-Therapy success
rel_15_comments	documentation of relapse 15-Remarks
rel_16	documentation of relapse 16-Has there been another surge?
rel_16_symptom	documentation of relapse 16-symptom
rel_16_description	documentation of relapse 16-Symptom description
rel_16_on_eye	documentation of relapse 16-Optic neuritis - eye
rel_16_on_pain	documentation of relapse 16-Optic neuritis - pain
rel_16_onset	documentation of relapse 16-Onset of symptoms
rel_16_therapy	documentation of relapse 16-therapy
rel_16_therapy_esc	documentation of relapse 16-Therapy escalation
rel_16_therapy_esc_med	documentation of relapse 16-Therapy escalation with
rel_16_therapy_success	documentation of relapse 16-Therapy success
rel_16_comments	documentation of relapse 16-Remarks
rel_17	documentation of relapse 17-Has there been another surge?
rel_17_symptom	documentation of relapse 17-symptom
rel_17_description	documentation of relapse 17-Symptom description
rel_17_on_eye	documentation of relapse 17-Optic neuritis - eye
rel_17_on_pain	documentation of relapse 17-Optic neuritis - pain
rel_17_onset	documentation of relapse 17-Onset of symptoms
rel_17_therapy	documentation of relapse 17-therapy
rel_17_therapy_esc	documentation of relapse 17-Therapy escalation
rel_17_therapy_esc_med	documentation of relapse 17-Therapy escalation with
rel_17_therapy_success	documentation of relapse 17-Therapy success
rel_17_comments	documentation of relapse 17-Remarks
rel_18	documentation of relapse 18-Has there been another surge?
rel_18_symptom	documentation of relapse 18-symptom
rel_18_description	documentation of relapse 18-Symptom description
rel_18_on_eye	documentation of relapse 18-Optic neuritis - eye
rel_18_on_pain	documentation of relapse 18-Optic neuritis - pain
rel_18_onset	documentation of relapse 18-Onset of symptoms
rel_18_therapy	documentation of relapse 18-therapy
rel_18_therapy_esc	documentation of relapse 18-Therapy escalation
rel_18_therapy_esc_med	documentation of relapse 18-Therapy escalation with
rel_18_therapy_success	documentation of relapse 18-Therapy success
rel_18_comments	documentation of relapse 18-Remarks
rel_19	documentation of relapse 19-Has there been another surge?
rel_19_symptom	documentation of relapse 19-symptom
rel_19_description	documentation of relapse 19-Symptom description
rel_19_on_eye	documentation of relapse 19-Optic neuritis - eye
rel_19_on_pain	documentation of relapse 19-Optic neuritis - pain
rel_19_onset	documentation of relapse 19-Onset of symptoms
rel_19_therapy	documentation of relapse 19-therapy
rel_19_therapy_esc	documentation of relapse 19-Therapy escalation

Table S1

variable code	Explanation
rel_19_therapy_esc_med	documentation of relapse 19-Therapy escalation with
rel_19_therapy_success	documentation of relapse 19-Therapy success
rel_19_comments	documentation of relapse 19-Remarks
rel_20	documentation of relapse 20-Has there been another surge?
rel_20_symptom	documentation of relapse 20-symptom
rel_20_description	documentation of relapse 20-Symptom description
rel_20_on_eye	documentation of relapse 20-Optic neuritis - eye
rel_20_on_pain	documentation of relapse 20-Optic neuritis - pain
rel_20_onset	documentation of relapse 20-Onset of symptoms
rel_20_therapy	documentation of relapse 20-therapy
rel_20_therapy_esc	documentation of relapse 20-Therapy escalation
rel_20_therapy_esc_med	documentation of relapse 20-Therapy escalation with
rel_20_therapy_success	documentation of relapse 20-Therapy success
rel_20_comments	documentation of relapse 20-Remarks
schubdokumentation_complete	documentation of relapses-Complete?
blood_taken	Blood work-Research blood was drawn
blood_date	Blood work-date
blood_time	Blood work-time
blood_heparin	Blood work-Number of Tubes - Heparin (BLUE)
blood_serum	Blood work-Number of tubes - serum (WHITE)
blood_edta	Blood work-Number of Tubes - EDTA (RED)
blood_paxgene	Blood work-Number of tubes - PAXGENE
blood_aqp4_ordered	Blood work-Was the AQP4-Ab determination commissioned from Stöcker Laboratory (IgG / IgM / IgA))
blood_mog_ordered	Blood work-Has the Stöcker laboratory been commissioned to determine MOG-Ab (IgG / IgM / IgA)?
forschungsblut_complete	Blood work-Complete?
lab_scan	autoantibody status-determination 1 -carried out?
lab_aqp4_name	autoantibody status-determination 1 -Laboratory findings scan
lab_aqp4_method	autoantibody status-determination 1 -laboratory
lab_aqp4_igg	autoantibody status-determination 1 -Test method
lab_aqp4_igm	autoantibody status-determination 1 -AQP-4 Ab IgG titer
lab_aqp4_iga	autoantibody status-determination 1 -AQP-4 Ab IgM titer
lab_mog_name	autoantibody status-determination 1 -AQP-4 Ab IgA titer
lab_mog_method	autoantibody status-determination 1 -laboratory
lab_mog_igg	autoantibody status-determination 1 -Test method
lab_mog_igm	autoantibody status-determination 1 -MOG Ab IgG titer
lab_mog_iga	autoantibody status-determination 1 -MOG Ab IgM titer
lab_additional_test	autoantibody status-determination 1 -MOG Ab IgA titer
lab_date_of_second_test	autoantibody status-determination 1 -Has there been another investigation?
lab_aqp4_name_2	autoantibody status-determination 2-Date of investigation
lab_aqp4_method_2	autoantibody status-determination 2-laboratory
lab_aqp4_igg_2	autoantibody status-determination 2-Test method
lab_aqp4_igm_2	autoantibody status-determination 2-AQP-4 Ab IgG titer
lab_aqp4_iga_2	autoantibody status-determination 2-AQP-4 Ab IgM titer
lab_mog_name_2	autoantibody status-determination 2-AQP-4 Ab IgA titer
lab_mog_method_2	autoantibody status-determination 2-laboratory
lab_mog_igg_2	autoantibody status-determination 2-Test method
lab_mog_igm_2	autoantibody status-determination 2-MOG Ab IgG titer
lab_mog_iga_2	autoantibody status-determination 2-MOG Ab IgM titer
lab_additional_test_2	autoantibody status-determination 2-MOG Ab IgA titer
lab_date_of_second_test_2	autoantibody status-determination 2-Has there been another investigation?
lab_aqp4_name_3	autoantibody status-determination 3-Date of investigation
lab_aqp4_method_3	autoantibody status-determination 3-laboratory
lab_aqp4_igg_3	autoantibody status-determination 3-Test method
lab_aqp4_igm_3	autoantibody status-determination 3-AQP-4 Ab IgG titer
lab_aqp4_iga_3	autoantibody status-determination 3-AQP-4 Ab IgM titer
lab_mog_name_3	autoantibody status-determination 3-AQP-4 Ab IgA titer
lab_mog_method_3	autoantibody status-determination 3-laboratory
lab_mog_igg_3	autoantibody status-determination 3-Test method
lab_mog_igm_3	autoantibody status-determination 3-MOG Ab IgG titer
lab_mog_iga_3	autoantibody status-determination 3-MOG Ab IgM titer
lab_additional_test_3	autoantibody status-determination 3-MOG Ab IgA titer
lab_date_of_second_test_3	autoantibody status-determination 3-Has there been another investigation?
lab_aqp4_name_4	autoantibody status-determination 4-Date of investigation
lab_aqp4_method_4	autoantibody status-determination 4-laboratory
lab_aqp4_igg_4	autoantibody status-determination 4-Test method
lab_aqp4_igm_4	autoantibody status-determination 4-AQP-4 Ab IgG titer
lab_aqp4_iga_4	autoantibody status-determination 4-AQP-4 Ab IgM titer
lab_mog_name_4	autoantibody status-determination 4-AQP-4 Ab IgA titer
lab_mog_method_4	autoantibody status-determination 4-laboratory
lab_mog_igg_4	autoantibody status-determination 4-Test method
lab_mog_igm_4	autoantibody status-determination 4-MOG Ab IgG titer
lab_mog_iga_4	autoantibody status-determination 4-MOG Ab IgM titer
lab_additional_test_4	autoantibody status-determination 4-MOG Ab IgA titer
lab_date_of_second_test_4	autoantibody status-determination 4-Has there been another investigation?
lab_aqp4_name_5	autoantibody status-determination 5-Date of investigation
lab_aqp4_method_5	autoantibody status-determination 5-laboratory
lab_aqp4_igg_5	autoantibody status-determination 5-Test method
lab_aqp4_igm_5	autoantibody status-determination 5-AQP-4 Ab IgG titer
lab_aqp4_iga_5	autoantibody status-determination 5-AQP-4 Ab IgM titer
lab_mog_name_5	autoantibody status-determination 5-AQP-4 Ab IgA titer
lab_mog_method_5	autoantibody status-determination 5-laboratory
lab_mog_igg_5	autoantibody status-determination 5-Test method
lab_mog_igm_5	autoantibody status-determination 5-MOG Ab IgG titer
lab_mog_iga_5	autoantibody status-determination 5-MOG Ab IgM titer
lab_additional_test_5	autoantibody status-determination 5-MOG Ab IgA titer
lab_date_of_second_test_5	autoantibody status-determination 5-Has there been another investigation?
lab_aqp4_name_6	autoantibody status-determination 6-Date of investigation
lab_aqp4_method_6	autoantibody status-determination 6-laboratory
lab_aqp4_igg_6	autoantibody status-determination 6-Test method
lab_aqp4_igm_6	autoantibody status-determination 6-AQP-4 Ab IgG titer
lab_aqp4_iga_6	autoantibody status-determination 6-AQP-4 Ab IgM titer
lab_mog_name_6	autoantibody status-determination 6-AQP-4 Ab IgA titer
lab_mog_method_6	autoantibody status-determination 6-laboratory
lab_mog_igg_6	autoantibody status-determination 6-Test method
lab_mog_igm_6	autoantibody status-determination 6-MOG Ab IgG titer
lab_mog_iga_6	autoantibody status-determination 6-MOG Ab IgM titer
lab_additional_test_6	autoantibody status-determination 6-MOG Ab IgA titer
lab_additional_tests_text	autoantibody status-additional determinations-Has there been another investigation?
antikrperstatus_und_labor_complete	autoantibody status-additional determinations-Information on further investigations
edss_date	EDSS (expanded disability status scale)-EDSS date
edss_grader	EDSS (expanded disability status scale)-EDSS grade
edss_comments	EDSS (expanded disability status scale)-Remarks

Table S1

variable code	Explanation
edss	EDSS (expanded disability status scale)-EDSS
edss_vs_raw	EDSS (expanded disability status scale)-FS Score - Visual System
edss_bs	EDSS (expanded disability status scale)-FS Score - brain stem
edss_py	EDSS (expanded disability status scale)-FS Score - Pyramidal System / Motor Skills
edss_cl	EDSS (expanded disability status scale)-FS Score - Cerebellum
edss_se	EDSS (expanded disability status scale)-FS Score - Sensory System
edss_bb	EDSS (expanded disability status scale)-FS - bowel and bladder function
edss_ce	EDSS (expanded disability status scale)-FS Score - Cerebral Functions
edss_amb	EDSS (expanded disability status scale)-FS - ambulation / walking ability
edss_expanded_disability_status_scale_complete	EDSS (expanded disability status scale)-Complete?
msfc_date	MSFC (Multiple Sclerosis Functional Composite)-date
msfc_grader	MSFC (Multiple Sclerosis Functional Composite)-MSFC grade
msfc_comments	MSFC (Multiple Sclerosis Functional Composite)-Remarks
t25fw_orthesis	MSFC (Multiple Sclerosis Functional Composite)-Timed 25-Foot Walk-Has the patient worn a foot or leg orthosis?
t25fw_support	MSFC (Multiple Sclerosis Functional Composite)-Timed 25-Foot Walk-Did the patient use an aid to walk?
t25fw_trial1	MSFC (Multiple Sclerosis Functional Composite)-Timed 25-Foot Walk-1st route
t25fw_trial2	MSFC (Multiple Sclerosis Functional Composite)-Timed 25-Foot Walk-2nd route
t25fw_usable	MSFC (Multiple Sclerosis Functional Composite)-Timed 25-Foot Walk-Timed 25-Foot Walk usable?
t25fw_usable_comments	MSFC (Multiple Sclerosis Functional Composite)-Timed 25-Foot Walk-Why?
t25fw_attempts	MSFC (Multiple Sclerosis Functional Composite)-Timed 25-Foot Walk-Did the patient take more than two attempts to successfully complete two tests?
t25fw_attempts_comments	MSFC (Multiple Sclerosis Functional Composite)-Timed 25-Foot Walk-Why?
hpt_handedness	MSFC (Multiple Sclerosis Functional Composite)-9-Hole Peg Test-Dominant hand
hpt_dom_trial1	MSFC (Multiple Sclerosis Functional Composite)-9-Hole Peg Test-Dominant hand - 1st round
hpt_dom_trial2	MSFC (Multiple Sclerosis Functional Composite)-9-Hole Peg Test-Dominant hand - 2nd round
hpt_ndom_trial1	MSFC (Multiple Sclerosis Functional Composite)-9-Hole Peg Test-Non-dominant hand - 1st round
hpt_ndom_trial2	MSFC (Multiple Sclerosis Functional Composite)-9-Hole Peg Test-Non-dominant hand - 2nd round
hpt_usable	MSFC (Multiple Sclerosis Functional Composite)-9-Hole Peg Test-9-Hole Peg Test usable?
hpt_usable_comments	MSFC (Multiple Sclerosis Functional Composite)-9-Hole Peg Test-Why?
hpt_attempts	MSFC (Multiple Sclerosis Functional Composite)-9-Hole Peg Test-Did the patient take more than two attempts to successfully complete two tests?
hpt_attempts_comments	MSFC (Multiple Sclerosis Functional Composite)-9-Hole Peg Test-Why?
msfct9_multiple_sclerosis_functional_composite_complete	MSFC (Multiple Sclerosis Functional Composite)-Complete?
mri_date	Medical imaging-MRI-date
mri_time	Medical imaging-MRI-time
mri_operator	Medical imaging-MRI-operator
mri_comments	Medical imaging-MRI-Remarks
mri_mprage	Medical imaging-MRI-MPRAGE
mri_t2space	Medical imaging-MRI-T2-3D-SPACE
mri_flair	Medical imaging-MRI-T2-3D FLAIR
mri_pdt2	Medical imaging-MRI-PD-T2
mri_dti	Medical imaging-MRI-DTI
mri_rsfmri	Medical imaging-MRI-rs-fMRI
mri_swi	Medical imaging-MRI-SWI
mri_perf	Medical imaging-MRI-PERF
mri_postgad_vibet1	Medical imaging-MRI-T1 post KM MPRAGE / VIBE
mri_spine	Medical imaging-MRI-Spine
mrs_frontal_cortex	Medical imaging-MRI-Frontal Cortex Spectroscopy
mrs_cerebellum	Medical imaging-MRI-Cerebellum spectroscopy
mri_xnat	Medical imaging-MRI-Record sent to XNAT
mri_safety	Medical imaging-MRI-Data set sent for safety assessment
mri_magnetresonanztomographie_complete	Medical imaging-MRI-Complete?
uhf_mri	Medical imaging-7 Tesla MRI-7T MRI performed?
uhf_mri_date	Medical imaging-7 Tesla MRI-date
uhf_mri_time	Medical imaging-7 Tesla MRI-time
uhf_mri_operator	Medical imaging-7 Tesla MRI-operator
uhf_mri_comments	Medical imaging-7 Tesla MRI-Remarks
uhf_mri_mprage	Medical imaging-7 Tesla MRI-7T-MPRAGE
uhf_mri_t2smapping	Medical imaging-7 Tesla MRI-T2 * mapping
uhf_mri_hrt2s	Medical imaging-7 Tesla MRI-7T high resolution T2 *
uhf_mri_mprage_flip	Medical imaging-7 Tesla MRI-7T MPR flip
uhf_mrs_t2flairspace	Medical imaging-7 Tesla MRI-7T-T2-FLAIR-SPACE
uhf_mri_swiqsm	Medical imaging-7 Tesla MRI-7T-SWI-QSM
uhf_mri_t2swi	Medical imaging-7 Tesla MRI-7T-T2-SWI
uhf_mri_xnat	Medical imaging-7 Tesla MRI-Record sent to XNAT
tmri_hochfeldmagnetresonanztomographie_complete	Medical imaging-7 Tesla MRI-Complete?
sienax_operator	Medical imaging-MRI based SIENAX-Brain tissue volume, normalised for head size-operator
sienax_fsl_version	Medical imaging-MRI based SIENAX-Brain tissue volume, normalised for head size-FSL version
sienax_mask_wb	Medical imaging-MRI based SIENAX-Brain tissue volume, normalised for head size-'Whole-brain segmentation' mask
sienax_mask_pc	Medical imaging-MRI based SIENAX-Brain tissue volume, normalised for head size-'Peripheral cortex masked segmentation'
sienax_mask_vent	Medical imaging-MRI based SIENAX-Brain tissue volume, normalised for head size-Ventricle masked segmentation
sienax_qc	Medical imaging-MRI based SIENAX-Brain tissue volume, normalised for head size-Result quality
sienax_pgrey	Medical imaging-MRI based SIENAX-Brain tissue volume, normalised for head size-PGREY
sienax_vcsf	Medical imaging-MRI based SIENAX-Brain tissue volume, normalised for head size-VCSF
sienax_gmv	Medical imaging-MRI based SIENAX-Brain tissue volume, normalised for head size-GMV
sienax_wmv	Medical imaging-MRI based SIENAX-Brain tissue volume, normalised for head size-WMV
sienax_bv	Medical imaging-MRI based SIENAX-Brain tissue volume, normalised for head size-Brain Volume
sienax_npgrey	Medical imaging-MRI based SIENAX-Brain tissue volume, normalised for head size-Normalized PGREY
sienax_nvcsf	Medical imaging-MRI based SIENAX-Brain tissue volume, normalised for head size-Normalized VCSF
sienax_ngmv	Medical imaging-MRI based SIENAX-Brain tissue volume, normalised for head size-Normalized GMV
sienax_nwmv	Medical imaging-MRI based SIENAX-Brain tissue volume, normalised for head size-Normalized WMV
sienax_nbv	Medical imaging-MRI based SIENAX-Brain tissue volume, normalised for head size-Normalized Brain Volume
sienax_hirnatrophie_complete	Medical imaging-MRI based SIENAX-Brain tissue volume, normalised for head size-Complete?
oct_datum	Medical imaging-Optical coherence tomography-date
oct_time	Medical imaging-Optical coherence tomography-time
oct_operator	Medical imaging-Optical coherence tomography-operator
oct_comments	Medical imaging-Optical coherence tomography-Remarks
oct_od_radius	Medical imaging-Optical coherence tomography-OD - Radius used
oct_os_radius	Medical imaging-Optical coherence tomography-OS - used radius
oct_onhrc	Medical imaging-Optical coherence tomography-ONH-RC APS
oct_ringscan_manual	Medical imaging-Optical coherence tomography-Manually centered ring scan
oct_onhv	Medical imaging-Optical coherence tomography-ONH-V
oct_ppolen	Medical imaging-Optical coherence tomography-PPole.N APS
oct_mac2	Medical imaging-Optical coherence tomography-Mac2
oct_ppolev	Medical imaging-Optical coherence tomography-PPole.V APS
oct_optische_kohrenztomographie_complete	Medical imaging-Optical coherence tomography-Complete?
vep_date	Neurophysiology-Visual evoked potential-date
vep_time	Neurophysiology-Visual evoked potential-time

Table S1

variable code	Explanation
vep_operator	Neurophysiology-Visual evoked potential-operator
vep_comments	Neurophysiology-Visual evoked potential-Remarks
vep_device	Neurophysiology-Visual evoked potential-device
vep_electrodes	Neurophysiology-Visual evoked potential-Electrodes
vep_protocol	Neurophysiology-Visual evoked potential-protocol
impedance_value	Neurophysiology-Visual evoked potential-Impedance (reference electrode)
impedance_value_active	Neurophysiology-Visual evoked potential-Impedance (active electrode)
vep_od_p100latency	Neurophysiology-Visual evoked potential-OD - p100 latency
vep_od_p100amplitude	Neurophysiology-Visual evoked potential-OD - p100 amplitude
vep_os_p100latency	Neurophysiology-Visual evoked potential-OS - p100 latency
vep_os_p100amplitude	Neurophysiology-Visual evoked potential-OS - p100 amplitude
vep_visuell_evozierte_potentiale_complete	Neurophysiology-Visual evoked potential-Complete?
mfvep_date	Neurophysiology-Multifocal Visual Evoked Potential-date
mfvep_tested	Neurophysiology-Multifocal Visual Evoked Potential-area tested
mfvep_time	Neurophysiology-Multifocal Visual Evoked Potential-time
mfvep_operator	Neurophysiology-Multifocal Visual Evoked Potential-operator
mfvep_operator_other	Neurophysiology-Multifocal Visual Evoked Potential-other operator
mfvep_test_id	Neurophysiology-Multifocal Visual Evoked Potential-Test ID
mfvep_sagital_distance	Neurophysiology-Multifocal Visual Evoked Potential-Sagital distance
mfvep_comments	Neurophysiology-Multifocal Visual Evoked Potential-Remarks
mfvep_impedance_value_ch1	Neurophysiology-Multifocal Visual Evoked Potential-Impedance Ch1
mfvep_impedance_value_ch2	Neurophysiology-Multifocal Visual Evoked Potential-Impedance Ch2
mfvep_both_eyes	Neurophysiology-Multifocal Visual Evoked Potential-Was the mfVEP performed for both eyes?
mfvep_eye	Neurophysiology-Multifocal Visual Evoked Potential-Which eye was examined?
mfvep_nr_measurements_od	Neurophysiology-Multifocal Visual Evoked Potential-How many measurement runs were OD carried out?
mfvep_nr_measurements_os	Neurophysiology-Multifocal Visual Evoked Potential-How many measurement runs were OS carried out?
mfvep_signal_quality_od	Neurophysiology-Multifocal Visual Evoked Potential-Is the signal quality OD at least in the yellow area?
mfvep_signal_quality_os	Neurophysiology-Multifocal Visual Evoked Potential-Is the OS signal quality at least in the yellow area?
mfvep_control	Neurophysiology-Multifocal Visual Evoked Potential-Have the measured values been checked?
mfvep_control_person	Neurophysiology-Multifocal Visual Evoked Potential-Who checked the measured values?
mfvep_od_latency_ch0	Neurophysiology-Multifocal Visual Evoked Potential-OD - latency -Ch0
mfvep_od_latency_ch1	Neurophysiology-Multifocal Visual Evoked Potential-OD - latency -Ch1
mfvep_od_latency_ch2	Neurophysiology-Multifocal Visual Evoked Potential-OD - latency -Ch2
mfvep_od_latency_ch3	Neurophysiology-Multifocal Visual Evoked Potential-OD - latency -Ch3
mfvep_od_latency_ch4	Neurophysiology-Multifocal Visual Evoked Potential-OD - latency -Ch4
mfvep_od_latency_ch5	Neurophysiology-Multifocal Visual Evoked Potential-OD - latency -Ch5
mfvep_od_latency_ch6	Neurophysiology-Multifocal Visual Evoked Potential-OD - latency -Ch6
mfvep_od_latency_ch7	Neurophysiology-Multifocal Visual Evoked Potential-OD - latency -Ch7
mfvep_od_latency_ch8	Neurophysiology-Multifocal Visual Evoked Potential-OD - latency -Ch8
mfvep_od_latency_ch9	Neurophysiology-Multifocal Visual Evoked Potential-OD - latency -Ch9
mfvep_od_latency_ch10	Neurophysiology-Multifocal Visual Evoked Potential-OD - latency -Ch10
mfvep_od_latency_ch11	Neurophysiology-Multifocal Visual Evoked Potential-OD - latency -Ch11
mfvep_od_latency_ch12	Neurophysiology-Multifocal Visual Evoked Potential-OD - latency -Ch12
mfvep_od_latency_ch13	Neurophysiology-Multifocal Visual Evoked Potential-OD - latency -Ch13
mfvep_od_latency_ch14	Neurophysiology-Multifocal Visual Evoked Potential-OD - latency -Ch14
mfvep_od_latency_ch15	Neurophysiology-Multifocal Visual Evoked Potential-OD - latency -Ch15
mfvep_od_latency_ch16	Neurophysiology-Multifocal Visual Evoked Potential-OD - latency -Ch16
mfvep_od_latency_ch17	Neurophysiology-Multifocal Visual Evoked Potential-OD - latency -Ch17
mfvep_od_latency_ch18	Neurophysiology-Multifocal Visual Evoked Potential-OD - latency -Ch18
mfvep_od_latency_ch19	Neurophysiology-Multifocal Visual Evoked Potential-OD - latency -Ch19
mfvep_od_latency_ch20	Neurophysiology-Multifocal Visual Evoked Potential-OD - latency -Ch20
mfvep_od_latency_ch21	Neurophysiology-Multifocal Visual Evoked Potential-OD - latency -Ch21
mfvep_od_latency_ch22	Neurophysiology-Multifocal Visual Evoked Potential-OD - latency -Ch22
mfvep_od_latency_ch23	Neurophysiology-Multifocal Visual Evoked Potential-OD - latency -Ch23
mfvep_od_latency_ch24	Neurophysiology-Multifocal Visual Evoked Potential-OD - latency -Ch24
mfvep_od_latency_ch25	Neurophysiology-Multifocal Visual Evoked Potential-OD - latency -Ch25
mfvep_od_latency_ch26	Neurophysiology-Multifocal Visual Evoked Potential-OD - latency -Ch26
mfvep_od_latency_ch27	Neurophysiology-Multifocal Visual Evoked Potential-OD - latency -Ch27
mfvep_od_latency_ch28	Neurophysiology-Multifocal Visual Evoked Potential-OD - latency -Ch28
mfvep_od_latency_ch29	Neurophysiology-Multifocal Visual Evoked Potential-OD - latency -Ch29
mfvep_od_latency_ch30	Neurophysiology-Multifocal Visual Evoked Potential-OD - latency -Ch30
mfvep_od_latency_ch31	Neurophysiology-Multifocal Visual Evoked Potential-OD - latency -Ch31
mfvep_od_latency_ch32	Neurophysiology-Multifocal Visual Evoked Potential-OD - latency -Ch32
mfvep_od_latency_ch33	Neurophysiology-Multifocal Visual Evoked Potential-OD - latency -Ch33
mfvep_od_latency_ch34	Neurophysiology-Multifocal Visual Evoked Potential-OD - latency -Ch34
mfvep_od_latency_ch35	Neurophysiology-Multifocal Visual Evoked Potential-OD - latency -Ch35
mfvep_od_latency_ch36	Neurophysiology-Multifocal Visual Evoked Potential-OD - latency -Ch36
mfvep_od_latency_ch37	Neurophysiology-Multifocal Visual Evoked Potential-OD - latency -Ch37
mfvep_od_latency_ch38	Neurophysiology-Multifocal Visual Evoked Potential-OD - latency -Ch38
mfvep_od_latency_ch39	Neurophysiology-Multifocal Visual Evoked Potential-OD - latency -Ch39
mfvep_od_latency_ch40	Neurophysiology-Multifocal Visual Evoked Potential-OD - latency -Ch40
mfvep_od_latency_ch41	Neurophysiology-Multifocal Visual Evoked Potential-OD - latency -Ch41
mfvep_od_latency_ch42	Neurophysiology-Multifocal Visual Evoked Potential-OD - latency -Ch42
mfvep_od_latency_ch43	Neurophysiology-Multifocal Visual Evoked Potential-OD - latency -Ch43
mfvep_od_latency_ch44	Neurophysiology-Multifocal Visual Evoked Potential-OD - latency -Ch44
mfvep_od_latency_ch45	Neurophysiology-Multifocal Visual Evoked Potential-OD - latency -Ch45
mfvep_od_latency_ch46	Neurophysiology-Multifocal Visual Evoked Potential-OD - latency -Ch46
mfvep_od_latency_ch47	Neurophysiology-Multifocal Visual Evoked Potential-OD - latency -Ch47
mfvep_od_latency_ch48	Neurophysiology-Multifocal Visual Evoked Potential-OD - latency -Ch48
mfvep_od_latency_ch49	Neurophysiology-Multifocal Visual Evoked Potential-OD - latency -Ch49
mfvep_od_latency_ch50	Neurophysiology-Multifocal Visual Evoked Potential-OD - latency -Ch50
mfvep_od_latency_ch51	Neurophysiology-Multifocal Visual Evoked Potential-OD - latency -Ch51
mfvep_od_latency_ch52	Neurophysiology-Multifocal Visual Evoked Potential-OD - latency -Ch52
mfvep_od_latency_ch53	Neurophysiology-Multifocal Visual Evoked Potential-OD - latency -Ch53
mfvep_od_latency_ch54	Neurophysiology-Multifocal Visual Evoked Potential-OD - latency -Ch54
mfvep_od_latency_ch55	Neurophysiology-Multifocal Visual Evoked Potential-OD - latency -Ch55
mfvep_os_latency_ch0	Neurophysiology-Multifocal Visual Evoked Potential-OS - Latency - Ch0
mfvep_os_latency_ch1	Neurophysiology-Multifocal Visual Evoked Potential-OS - Latency - Ch1
mfvep_os_latency_ch2	Neurophysiology-Multifocal Visual Evoked Potential-OS - Latency - Ch2
mfvep_os_latency_ch3	Neurophysiology-Multifocal Visual Evoked Potential-OS - Latency - Ch3
mfvep_os_latency_ch4	Neurophysiology-Multifocal Visual Evoked Potential-OS - Latency - Ch4
mfvep_os_latency_ch5	Neurophysiology-Multifocal Visual Evoked Potential-OS - Latency - Ch5
mfvep_os_latency_ch6	Neurophysiology-Multifocal Visual Evoked Potential-OS - Latency - Ch6
mfvep_os_latency_ch7	Neurophysiology-Multifocal Visual Evoked Potential-OS - Latency - Ch7
mfvep_os_latency_ch8	Neurophysiology-Multifocal Visual Evoked Potential-OS - Latency - Ch8
mfvep_os_latency_ch9	Neurophysiology-Multifocal Visual Evoked Potential-OS - Latency - Ch9
mfvep_os_latency_ch10	Neurophysiology-Multifocal Visual Evoked Potential-OS - Latency - Ch10
mfvep_os_latency_ch11	Neurophysiology-Multifocal Visual Evoked Potential-OS - Latency - Ch11
mfvep_os_latency_ch12	Neurophysiology-Multifocal Visual Evoked Potential-OS - Latency - Ch12
mfvep_os_latency_ch13	Neurophysiology-Multifocal Visual Evoked Potential-OS - Latency - Ch13
mfvep_os_latency_ch14	Neurophysiology-Multifocal Visual Evoked Potential-OS - Latency - Ch14
mfvep_os_latency_ch15	Neurophysiology-Multifocal Visual Evoked Potential-OS - Latency - Ch15

Table S1

variable code	Explanation
mfvep_os_latency_ch16	Neurophysiology-Multifocal Visual Evoked Potential-OS - Latency - Ch16
mfvep_os_latency_ch17	Neurophysiology-Multifocal Visual Evoked Potential-OS - Latency - Ch17
mfvep_os_latency_ch18	Neurophysiology-Multifocal Visual Evoked Potential-OS - Latency - Ch18
mfvep_os_latency_ch19	Neurophysiology-Multifocal Visual Evoked Potential-OS - Latency - Ch19
mfvep_os_latency_ch20	Neurophysiology-Multifocal Visual Evoked Potential-OS - Latency - Ch20
mfvep_os_latency_ch21	Neurophysiology-Multifocal Visual Evoked Potential-OS - Latency - Ch21
mfvep_os_latency_ch22	Neurophysiology-Multifocal Visual Evoked Potential-OS - Latency - Ch22
mfvep_os_latency_ch23	Neurophysiology-Multifocal Visual Evoked Potential-OS - Latency - Ch23
mfvep_os_latency_ch24	Neurophysiology-Multifocal Visual Evoked Potential-OS - Latency - Ch24
mfvep_os_latency_ch25	Neurophysiology-Multifocal Visual Evoked Potential-OS - Latency - Ch25
mfvep_os_latency_ch26	Neurophysiology-Multifocal Visual Evoked Potential-OS - Latency - Ch26
mfvep_os_latency_ch27	Neurophysiology-Multifocal Visual Evoked Potential-OS - Latency - Ch27
mfvep_os_latency_ch28	Neurophysiology-Multifocal Visual Evoked Potential-OS - Latency - Ch28
mfvep_os_latency_ch29	Neurophysiology-Multifocal Visual Evoked Potential-OS - Latency - Ch29
mfvep_os_latency_ch30	Neurophysiology-Multifocal Visual Evoked Potential-OS - Latency - Ch30
mfvep_os_latency_ch31	Neurophysiology-Multifocal Visual Evoked Potential-OS - Latency - Ch31
mfvep_os_latency_ch32	Neurophysiology-Multifocal Visual Evoked Potential-OS - Latency - Ch32
mfvep_os_latency_ch33	Neurophysiology-Multifocal Visual Evoked Potential-OS - Latency - Ch33
mfvep_os_latency_ch34	Neurophysiology-Multifocal Visual Evoked Potential-OS - Latency - Ch34
mfvep_os_latency_ch35	Neurophysiology-Multifocal Visual Evoked Potential-OS - Latency - Ch35
mfvep_os_latency_ch36	Neurophysiology-Multifocal Visual Evoked Potential-OS - Latency - Ch36
mfvep_os_latency_ch37	Neurophysiology-Multifocal Visual Evoked Potential-OS - Latency - Ch37
mfvep_os_latency_ch38	Neurophysiology-Multifocal Visual Evoked Potential-OS - Latency - Ch38
mfvep_os_latency_ch39	Neurophysiology-Multifocal Visual Evoked Potential-OS - Latency - Ch39
mfvep_os_latency_ch40	Neurophysiology-Multifocal Visual Evoked Potential-OS - Latency - Ch40
mfvep_os_latency_ch41	Neurophysiology-Multifocal Visual Evoked Potential-OS - Latency - Ch41
mfvep_os_latency_ch42	Neurophysiology-Multifocal Visual Evoked Potential-OS - Latency - Ch42
mfvep_os_latency_ch43	Neurophysiology-Multifocal Visual Evoked Potential-OS - Latency - Ch43
mfvep_os_latency_ch44	Neurophysiology-Multifocal Visual Evoked Potential-OS - Latency - Ch44
mfvep_os_latency_ch45	Neurophysiology-Multifocal Visual Evoked Potential-OS - Latency - Ch45
mfvep_os_latency_ch46	Neurophysiology-Multifocal Visual Evoked Potential-OS - Latency - Ch46
mfvep_os_latency_ch47	Neurophysiology-Multifocal Visual Evoked Potential-OS - Latency - Ch47
mfvep_os_latency_ch48	Neurophysiology-Multifocal Visual Evoked Potential-OS - Latency - Ch48
mfvep_os_latency_ch49	Neurophysiology-Multifocal Visual Evoked Potential-OS - Latency - Ch49
mfvep_os_latency_ch50	Neurophysiology-Multifocal Visual Evoked Potential-OS - Latency - Ch50
mfvep_os_latency_ch51	Neurophysiology-Multifocal Visual Evoked Potential-OS - Latency - Ch51
mfvep_os_latency_ch52	Neurophysiology-Multifocal Visual Evoked Potential-OS - Latency - Ch52
mfvep_os_latency_ch53	Neurophysiology-Multifocal Visual Evoked Potential-OS - Latency - Ch53
mfvep_os_latency_ch54	Neurophysiology-Multifocal Visual Evoked Potential-OS - Latency - Ch54
mfvep_os_latency_ch55	Neurophysiology-Multifocal Visual Evoked Potential-OS - Latency - Ch55
mfvep_od_amplitude_ch0	Neurophysiology-Multifocal Visual Evoked Potential-OD - amplitude - Ch0
mfvep_od_amplitude_ch1	Neurophysiology-Multifocal Visual Evoked Potential-OD - amplitude - Ch1
mfvep_od_amplitude_ch2	Neurophysiology-Multifocal Visual Evoked Potential-OD - amplitude - Ch2
mfvep_od_amplitude_ch3	Neurophysiology-Multifocal Visual Evoked Potential-OD - amplitude - Ch3
mfvep_od_amplitude_ch4	Neurophysiology-Multifocal Visual Evoked Potential-OD - amplitude - Ch4
mfvep_od_amplitude_ch5	Neurophysiology-Multifocal Visual Evoked Potential-OD - amplitude - Ch5
mfvep_od_amplitude_ch6	Neurophysiology-Multifocal Visual Evoked Potential-OD - amplitude - Ch6
mfvep_od_amplitude_ch7	Neurophysiology-Multifocal Visual Evoked Potential-OD - amplitude - Ch7
mfvep_od_amplitude_ch8	Neurophysiology-Multifocal Visual Evoked Potential-OD - amplitude - Ch8
mfvep_od_amplitude_ch9	Neurophysiology-Multifocal Visual Evoked Potential-OD - amplitude - Ch9
mfvep_od_amplitude_ch10	Neurophysiology-Multifocal Visual Evoked Potential-OD - amplitude - Ch10
mfvep_od_amplitude_ch11	Neurophysiology-Multifocal Visual Evoked Potential-OD - amplitude - Ch11
mfvep_od_amplitude_ch12	Neurophysiology-Multifocal Visual Evoked Potential-OD - amplitude - Ch12
mfvep_od_amplitude_ch13	Neurophysiology-Multifocal Visual Evoked Potential-OD - amplitude - Ch13
mfvep_od_amplitude_ch14	Neurophysiology-Multifocal Visual Evoked Potential-OD - amplitude - Ch14
mfvep_od_amplitude_ch15	Neurophysiology-Multifocal Visual Evoked Potential-OD - amplitude - Ch15
mfvep_od_amplitude_ch16	Neurophysiology-Multifocal Visual Evoked Potential-OD - amplitude - Ch16
mfvep_od_amplitude_ch17	Neurophysiology-Multifocal Visual Evoked Potential-OD - amplitude - Ch17
mfvep_od_amplitude_ch18	Neurophysiology-Multifocal Visual Evoked Potential-OD - amplitude - Ch18
mfvep_od_amplitude_ch19	Neurophysiology-Multifocal Visual Evoked Potential-OD - amplitude - Ch19
mfvep_od_amplitude_ch20	Neurophysiology-Multifocal Visual Evoked Potential-OD - amplitude - Ch20
mfvep_od_amplitude_ch21	Neurophysiology-Multifocal Visual Evoked Potential-OD - amplitude - Ch21
mfvep_od_amplitude_ch22	Neurophysiology-Multifocal Visual Evoked Potential-OD - amplitude - Ch22
mfvep_od_amplitude_ch23	Neurophysiology-Multifocal Visual Evoked Potential-OD - amplitude - Ch23
mfvep_od_amplitude_ch24	Neurophysiology-Multifocal Visual Evoked Potential-OD - amplitude - Ch24
mfvep_od_amplitude_ch25	Neurophysiology-Multifocal Visual Evoked Potential-OD - amplitude - Ch25
mfvep_od_amplitude_ch26	Neurophysiology-Multifocal Visual Evoked Potential-OD - amplitude - Ch26
mfvep_od_amplitude_ch27	Neurophysiology-Multifocal Visual Evoked Potential-OD - amplitude - Ch27
mfvep_od_amplitude_ch28	Neurophysiology-Multifocal Visual Evoked Potential-OD - amplitude - Ch28
mfvep_od_amplitude_ch29	Neurophysiology-Multifocal Visual Evoked Potential-OD - amplitude - Ch29
mfvep_od_amplitude_ch30	Neurophysiology-Multifocal Visual Evoked Potential-OD - amplitude - Ch30
mfvep_od_amplitude_ch31	Neurophysiology-Multifocal Visual Evoked Potential-OD - amplitude - Ch31
mfvep_od_amplitude_ch32	Neurophysiology-Multifocal Visual Evoked Potential-OD - amplitude - Ch32
mfvep_od_amplitude_ch33	Neurophysiology-Multifocal Visual Evoked Potential-OD - amplitude - Ch33
mfvep_od_amplitude_ch34	Neurophysiology-Multifocal Visual Evoked Potential-OD - amplitude - Ch34
mfvep_od_amplitude_ch35	Neurophysiology-Multifocal Visual Evoked Potential-OD - amplitude - Ch35
mfvep_od_amplitude_ch36	Neurophysiology-Multifocal Visual Evoked Potential-OD - amplitude - Ch36
mfvep_od_amplitude_ch37	Neurophysiology-Multifocal Visual Evoked Potential-OD - amplitude - Ch37
mfvep_od_amplitude_ch38	Neurophysiology-Multifocal Visual Evoked Potential-OD - amplitude - Ch38
mfvep_od_amplitude_ch39	Neurophysiology-Multifocal Visual Evoked Potential-OD - amplitude - Ch39
mfvep_od_amplitude_ch40	Neurophysiology-Multifocal Visual Evoked Potential-OD - amplitude - Ch40
mfvep_od_amplitude_ch41	Neurophysiology-Multifocal Visual Evoked Potential-OD - amplitude - Ch41
mfvep_od_amplitude_ch42	Neurophysiology-Multifocal Visual Evoked Potential-OD - amplitude - Ch42
mfvep_od_amplitude_ch43	Neurophysiology-Multifocal Visual Evoked Potential-OD - amplitude - Ch43
mfvep_od_amplitude_ch44	Neurophysiology-Multifocal Visual Evoked Potential-OD - amplitude - Ch44
mfvep_od_amplitude_ch45	Neurophysiology-Multifocal Visual Evoked Potential-OD - amplitude - Ch45
mfvep_od_amplitude_ch46	Neurophysiology-Multifocal Visual Evoked Potential-OD - amplitude - Ch46
mfvep_od_amplitude_ch47	Neurophysiology-Multifocal Visual Evoked Potential-OD - amplitude - Ch47
mfvep_od_amplitude_ch48	Neurophysiology-Multifocal Visual Evoked Potential-OD - amplitude - Ch48
mfvep_od_amplitude_ch49	Neurophysiology-Multifocal Visual Evoked Potential-OD - amplitude - Ch49
mfvep_od_amplitude_ch50	Neurophysiology-Multifocal Visual Evoked Potential-OD - amplitude - Ch50
mfvep_od_amplitude_ch51	Neurophysiology-Multifocal Visual Evoked Potential-OD - amplitude - Ch51
mfvep_od_amplitude_ch52	Neurophysiology-Multifocal Visual Evoked Potential-OD - amplitude - Ch52
mfvep_od_amplitude_ch53	Neurophysiology-Multifocal Visual Evoked Potential-OD - amplitude - Ch53
mfvep_od_amplitude_ch54	Neurophysiology-Multifocal Visual Evoked Potential-OD - amplitude - Ch54
mfvep_od_amplitude_ch55	Neurophysiology-Multifocal Visual Evoked Potential-OD - amplitude - Ch55
mfvep_os_amplitude_ch0	Neurophysiology-Multifocal Visual Evoked Potential-OS - amplitude - Ch0
mfvep_os_amplitude_ch1	Neurophysiology-Multifocal Visual Evoked Potential-OS - amplitude - Ch1
mfvep_os_amplitude_ch2	Neurophysiology-Multifocal Visual Evoked Potential-OS - amplitude - Ch2
mfvep_os_amplitude_ch3	Neurophysiology-Multifocal Visual Evoked Potential-OS - amplitude - Ch3
mfvep_os_amplitude_ch4	Neurophysiology-Multifocal Visual Evoked Potential-OS - amplitude - Ch4
mfvep_os_amplitude_ch5	Neurophysiology-Multifocal Visual Evoked Potential-OS - amplitude - Ch5

Table S1

variable code	Explanation
mfvep_os_amplitude_ch6	Neurophysiology-Multifocal Visual Evoked Potential-OS - amplitude - Ch6
mfvep_os_amplitude_ch7	Neurophysiology-Multifocal Visual Evoked Potential-OS - amplitude - Ch7
mfvep_os_amplitude_ch8	Neurophysiology-Multifocal Visual Evoked Potential-OS - amplitude - Ch8
mfvep_os_amplitude_ch9	Neurophysiology-Multifocal Visual Evoked Potential-OS - amplitude - Ch9
mfvep_os_amplitude_ch10	Neurophysiology-Multifocal Visual Evoked Potential-OS - amplitude - Ch10
mfvep_os_amplitude_ch11	Neurophysiology-Multifocal Visual Evoked Potential-OS - amplitude - Ch11
mfvep_os_amplitude_ch12	Neurophysiology-Multifocal Visual Evoked Potential-OS - amplitude - Ch12
mfvep_os_amplitude_ch13	Neurophysiology-Multifocal Visual Evoked Potential-OS - amplitude - Ch13
mfvep_os_amplitude_ch14	Neurophysiology-Multifocal Visual Evoked Potential-OS - amplitude - Ch14
mfvep_os_amplitude_ch15	Neurophysiology-Multifocal Visual Evoked Potential-OS - amplitude - Ch15
mfvep_os_amplitude_ch16	Neurophysiology-Multifocal Visual Evoked Potential-OS - amplitude - Ch16
mfvep_os_amplitude_ch17	Neurophysiology-Multifocal Visual Evoked Potential-OS - amplitude - Ch17
mfvep_os_amplitude_ch18	Neurophysiology-Multifocal Visual Evoked Potential-OS - amplitude - Ch18
mfvep_os_amplitude_ch19	Neurophysiology-Multifocal Visual Evoked Potential-OS - amplitude - Ch19
mfvep_os_amplitude_ch20	Neurophysiology-Multifocal Visual Evoked Potential-OS - amplitude - Ch20
mfvep_os_amplitude_ch21	Neurophysiology-Multifocal Visual Evoked Potential-OS - amplitude - Ch21
mfvep_os_amplitude_ch22	Neurophysiology-Multifocal Visual Evoked Potential-OS - amplitude - Ch22
mfvep_os_amplitude_ch23	Neurophysiology-Multifocal Visual Evoked Potential-OS - amplitude - Ch23
mfvep_os_amplitude_ch24	Neurophysiology-Multifocal Visual Evoked Potential-OS - amplitude - Ch24
mfvep_os_amplitude_ch25	Neurophysiology-Multifocal Visual Evoked Potential-OS - amplitude - Ch25
mfvep_os_amplitude_ch26	Neurophysiology-Multifocal Visual Evoked Potential-OS - amplitude - Ch26
mfvep_os_amplitude_ch27	Neurophysiology-Multifocal Visual Evoked Potential-OS - amplitude - Ch27
mfvep_os_amplitude_ch28	Neurophysiology-Multifocal Visual Evoked Potential-OS - amplitude - Ch28
mfvep_os_amplitude_ch29	Neurophysiology-Multifocal Visual Evoked Potential-OS - amplitude - Ch29
mfvep_os_amplitude_ch30	Neurophysiology-Multifocal Visual Evoked Potential-OS - amplitude - Ch30
mfvep_os_amplitude_ch31	Neurophysiology-Multifocal Visual Evoked Potential-OS - amplitude - Ch31
mfvep_os_amplitude_ch32	Neurophysiology-Multifocal Visual Evoked Potential-OS - amplitude - Ch32
mfvep_os_amplitude_ch33	Neurophysiology-Multifocal Visual Evoked Potential-OS - amplitude - Ch33
mfvep_os_amplitude_ch34	Neurophysiology-Multifocal Visual Evoked Potential-OS - amplitude - Ch34
mfvep_os_amplitude_ch35	Neurophysiology-Multifocal Visual Evoked Potential-OS - amplitude - Ch35
mfvep_os_amplitude_ch36	Neurophysiology-Multifocal Visual Evoked Potential-OS - amplitude - Ch36
mfvep_os_amplitude_ch37	Neurophysiology-Multifocal Visual Evoked Potential-OS - amplitude - Ch37
mfvep_os_amplitude_ch38	Neurophysiology-Multifocal Visual Evoked Potential-OS - amplitude - Ch38
mfvep_os_amplitude_ch39	Neurophysiology-Multifocal Visual Evoked Potential-OS - amplitude - Ch39
mfvep_os_amplitude_ch40	Neurophysiology-Multifocal Visual Evoked Potential-OS - amplitude - Ch40
mfvep_os_amplitude_ch41	Neurophysiology-Multifocal Visual Evoked Potential-OS - amplitude - Ch41
mfvep_os_amplitude_ch42	Neurophysiology-Multifocal Visual Evoked Potential-OS - amplitude - Ch42
mfvep_os_amplitude_ch43	Neurophysiology-Multifocal Visual Evoked Potential-OS - amplitude - Ch43
mfvep_os_amplitude_ch44	Neurophysiology-Multifocal Visual Evoked Potential-OS - amplitude - Ch44
mfvep_os_amplitude_ch45	Neurophysiology-Multifocal Visual Evoked Potential-OS - amplitude - Ch45
mfvep_os_amplitude_ch46	Neurophysiology-Multifocal Visual Evoked Potential-OS - amplitude - Ch46
mfvep_os_amplitude_ch47	Neurophysiology-Multifocal Visual Evoked Potential-OS - amplitude - Ch47
mfvep_os_amplitude_ch48	Neurophysiology-Multifocal Visual Evoked Potential-OS - amplitude - Ch48
mfvep_os_amplitude_ch49	Neurophysiology-Multifocal Visual Evoked Potential-OS - amplitude - Ch49
mfvep_os_amplitude_ch50	Neurophysiology-Multifocal Visual Evoked Potential-OS - amplitude - Ch50
mfvep_os_amplitude_ch51	Neurophysiology-Multifocal Visual Evoked Potential-OS - amplitude - Ch51
mfvep_os_amplitude_ch52	Neurophysiology-Multifocal Visual Evoked Potential-OS - amplitude - Ch52
mfvep_os_amplitude_ch53	Neurophysiology-Multifocal Visual Evoked Potential-OS - amplitude - Ch53
mfvep_os_amplitude_ch54	Neurophysiology-Multifocal Visual Evoked Potential-OS - amplitude - Ch54
mfvep_os_amplitude_ch55	Neurophysiology-Multifocal Visual Evoked Potential-OS - amplitude - Ch55
mfvep_mean_od_latency	Neurophysiology-Multifocal Visual Evoked Potential-OD - mean value - latency
mfvep_mean_od_amplitude	Neurophysiology-Multifocal Visual Evoked Potential-OD - mean value - amplitude
mfvep_mean_os_latency	Neurophysiology-Multifocal Visual Evoked Potential-OS Average Latency
mfvep_mean_os_amplitude	Neurophysiology-Multifocal Visual Evoked Potential-OS - mean value - amplitude
mfvep_complete	Neurophysiology-Multifocal Visual Evoked Potential-Complete?
va_date	Neurophysiology-visual acuity-date
va_time	Neurophysiology-visual acuity-time
va_operator	Neurophysiology-visual acuity-operator
va_comments	Neurophysiology-visual acuity-Remarks
va_correction	Neurophysiology-visual acuity-correction
va_od	Neurophysiology-visual acuity-OD - VA measurement
va_os	Neurophysiology-visual acuity-OS - VA measurement
va_ou	Neurophysiology-visual acuity-OU - VA measurement
etdrs_od_la	ETDRS (Early-Treatment Diabetic Retinopathy Study)-OD - ETDRS 100% Letter Acuity
etdrs_od_va	ETDRS (Early-Treatment Diabetic Retinopathy Study)-OD - ETDRS 100% decimal visual acuity
etdrs_os_la	ETDRS (Early-Treatment Diabetic Retinopathy Study)-OS - ETDRS 100% Letter Acuity
etdrs_os_va	ETDRS (Early-Treatment Diabetic Retinopathy Study)-OS - ETDRS 100% decimal visual acuity
etdrs_ou_la	ETDRS (Early-Treatment Diabetic Retinopathy Study)-OU - ETDRS 100% Letter Acuity
etdrs_ou_va	ETDRS (Early-Treatment Diabetic Retinopathy Study)-OU - ETDRS 100% decimal visual acuity
sloan25_od_la	ETDRS (Early-Treatment Diabetic Retinopathy Study)-OD - Sloan 2.5% Letter Acuity
sloan25_od_va	ETDRS (Early-Treatment Diabetic Retinopathy Study)-OD - Sloan 2.5% decimal visual acuity
sloan25_os_la	ETDRS (Early-Treatment Diabetic Retinopathy Study)-OS - Sloan 2.5% Letter Acuity
sloan25_os_va	ETDRS (Early-Treatment Diabetic Retinopathy Study)-OS - Sloan 2.5% decimal visual acuity
sloan25_ou_la	ETDRS (Early-Treatment Diabetic Retinopathy Study)-OU - Sloan 2.5% Letter Acuity
sloan25_ou_va	ETDRS (Early-Treatment Diabetic Retinopathy Study)-OU - Sloan 2.5% decimal visual acuity
va_hoch_und_niedrigkontrastsehtafeln_complete	Neurophysiology-visual acuity-low Contrast Test-Complete?
fact_date	Neurophysiology-visual acuity-Vision Contrast Test-Functional Acuity Contrast Test (FACT) -date
fact_operator	Neurophysiology-visual acuity-Vision Contrast Test-Functional Acuity Contrast Test (FACT) -operator
fact_od_a	Neurophysiology-visual acuity-Vision Contrast Test-Functional Acuity Contrast Test (FACT) -OD - A
fact_od_b	Neurophysiology-visual acuity-Vision Contrast Test-Functional Acuity Contrast Test (FACT) -OD - B
fact_od_c	Neurophysiology-visual acuity-Vision Contrast Test-Functional Acuity Contrast Test (FACT) -OD - C
fact_od_d	Neurophysiology-visual acuity-Vision Contrast Test-Functional Acuity Contrast Test (FACT) -OD - D
fact_od_e	Neurophysiology-visual acuity-Vision Contrast Test-Functional Acuity Contrast Test (FACT) -OD - E
fact_od_auc	Neurophysiology-visual acuity-Vision Contrast Test-Functional Acuity Contrast Test (FACT) -OD - AUC
fact_os_a	Neurophysiology-visual acuity-Vision Contrast Test-Functional Acuity Contrast Test (FACT) -OS - A
fact_os_b	Neurophysiology-visual acuity-Vision Contrast Test-Functional Acuity Contrast Test (FACT) -OS - B
fact_os_c	Neurophysiology-visual acuity-Vision Contrast Test-Functional Acuity Contrast Test (FACT) -OS - C
fact_os_d	Neurophysiology-visual acuity-Vision Contrast Test-Functional Acuity Contrast Test (FACT) -OS - D
fact_os_e	Neurophysiology-visual acuity-Vision Contrast Test-Functional Acuity Contrast Test (FACT) -OS - E
fact_os_auc	Neurophysiology-visual acuity-Vision Contrast Test-Functional Acuity Contrast Test (FACT) -OS - AUC
fact_ou_a	Neurophysiology-visual acuity-Vision Contrast Test-Functional Acuity Contrast Test (FACT) -OU - A
fact_ou_b	Neurophysiology-visual acuity-Vision Contrast Test-Functional Acuity Contrast Test (FACT) -OU - B
fact_ou_c	Neurophysiology-visual acuity-Vision Contrast Test-Functional Acuity Contrast Test (FACT) -OU - C
fact_ou_d	Neurophysiology-visual acuity-Vision Contrast Test-Functional Acuity Contrast Test (FACT) -OU - D
fact_ou_e	Neurophysiology-visual acuity-Vision Contrast Test-Functional Acuity Contrast Test (FACT) -OU - E
fact_ou_auc	Neurophysiology-visual acuity-Vision Contrast Test-Functional Acuity Contrast Test (FACT) -OU - AUC
fact_kontrastsehen_complete	Neurophysiology-visual acuity-Vision Contrast Test-Functional Acuity Contrast Test (FACT) -Complete?
rkt_date	Neurophysiology-visual acuity-Kerato-Refracto-Tonometry-date
rkt_time	Neurophysiology-visual acuity-Kerato-Refracto-Tonometry-Time
rkt_operator	Neurophysiology-visual acuity-Kerato-Refracto-Tonometry-operator
rkt_comments	Neurophysiology-visual acuity-Kerato-Refracto-Tonometry-Remarks
ref_od_sph	Neurophysiology-visual acuity-Kerato-Refracto-Tonometry-OD - Sph

Table S1

variable code	Explanation
ref_od_cyl	Neurophysiology-visual acuity-Kerato-Refracto-Tonometry-OD - Cyl
ref_od_axis	Neurophysiology-visual acuity-Kerato-Refracto-Tonometry-OD - Axis
ref_os_sph	Neurophysiology-visual acuity-Kerato-Refracto-Tonometry-OS - Sph
ref_os_cyl	Neurophysiology-visual acuity-Kerato-Refracto-Tonometry-OS - Cyl
ref_os_axis	Neurophysiology-visual acuity-Kerato-Refracto-Tonometry-OS - Axis
kerato_od_rave	Neurophysiology-visual acuity-Kerato-Refracto-Tonometry-OD - R average
kerato_os_rave	Neurophysiology-visual acuity-Kerato-Refracto-Tonometry-OS - R average
tono_od	Neurophysiology-visual acuity-Kerato-Refracto-Tonometry-OD - mean pressure
tono_os	Neurophysiology-visual acuity-Kerato-Refracto-Tonometry-OS - mean pressure
rkt_autorefraktokeratotonometrie_complete	Neurophysiology-visual acuity-Kerato-Refracto-Tonometry-Complete?
peri_date	Neurophysiology-visual acuity-Perimetry-date
peri_time	Neurophysiology-visual acuity-Perimetry-time
peri_operator	Neurophysiology-visual acuity-Perimetry-operator
peri_comments	Neurophysiology-visual acuity-Perimetry-Remarks
peri_device	Neurophysiology-visual acuity-Perimetry-device
peri_protocol	Neurophysiology-visual acuity-Perimetry-protocol
peri_protocol_other	Neurophysiology-visual acuity-Perimetry-Information on the protocol used
peri_od_md	Neurophysiology-visual acuity-Perimetry-MD right eye
peri_od_psd	Neurophysiology-visual acuity-Perimetry-PSD right eye
peri_os_md	Neurophysiology-visual acuity-Perimetry-MD left eye
peri_os_psd	Neurophysiology-visual acuity-Perimetry-PSD left eye
perimetrie_complete	Neurophysiology-visual acuity-Perimetry-Complete?
hrr_date	Neurophysiology-visual acuity-color vision-date
hrr_time	Neurophysiology-visual acuity-color vision-time
hrr_operator	Neurophysiology-visual acuity-color vision-operator
hrr_comments	Neurophysiology-visual acuity-color vision-Remarks
hrr_light	Neurophysiology-visual acuity-color vision-lighting
hrr_od_plate5	Neurophysiology-visual acuity-color vision-Plate 5
hrr_od_plate6	Neurophysiology-visual acuity-color vision-Plate 6
hrr_od_plate7	Neurophysiology-visual acuity-color vision-Plate 7
hrr_od_plate8	Neurophysiology-visual acuity-color vision-Plate 8
hrr_od_plate9	Neurophysiology-visual acuity-color vision-Plate 9
hrr_od_plate10	Neurophysiology-visual acuity-color vision-Plate 10
hrr_od_plate11	Neurophysiology-visual acuity-color vision-Plate 11
hrr_od_plate12	Neurophysiology-visual acuity-color vision-Plate 12
hrr_od_plate13	Neurophysiology-visual acuity-color vision-Plate 13
hrr_od_plate14	Neurophysiology-visual acuity-color vision-Plate 14
hrr_od_plate15	Neurophysiology-visual acuity-color vision-Plate 15
hrr_od_plate16	Neurophysiology-visual acuity-color vision-Plate 16
hrr_od_plate17	Neurophysiology-visual acuity-color vision-Plate 17
hrr_od_plate18	Neurophysiology-visual acuity-color vision-Plate 18
hrr_od_plate19	Neurophysiology-visual acuity-color vision-Plate 19
hrr_od_plate20	Neurophysiology-visual acuity-color vision-Plate 20
hrr_od_plate21	Neurophysiology-visual acuity-color vision-Plate 21
hrr_od_plate22	Neurophysiology-visual acuity-color vision-Plate 22
hrr_od_plate23	Neurophysiology-visual acuity-color vision-Plate 23
hrr_od_plate24	Neurophysiology-visual acuity-color vision-Plate 24
hrr_os_plate5	Neurophysiology-visual acuity-color vision-Plate 5
hrr_os_plate6	Neurophysiology-visual acuity-color vision-Plate 6
hrr_os_plate7	Neurophysiology-visual acuity-color vision-Plate 7
hrr_os_plate8	Neurophysiology-visual acuity-color vision-Plate 8
hrr_os_plate9	Neurophysiology-visual acuity-color vision-Plate 9
hrr_os_plate10	Neurophysiology-visual acuity-color vision-Plate 10
hrr_os_plate11	Neurophysiology-visual acuity-color vision-Plate 11
hrr_os_plate12	Neurophysiology-visual acuity-color vision-Plate 12
hrr_os_plate13	Neurophysiology-visual acuity-color vision-Plate 13
hrr_os_plate14	Neurophysiology-visual acuity-color vision-Plate 14
hrr_os_plate15	Neurophysiology-visual acuity-color vision-Plate 15
hrr_os_plate16	Neurophysiology-visual acuity-color vision-Plate 16
hrr_os_plate17	Neurophysiology-visual acuity-color vision-Plate 17
hrr_os_plate18	Neurophysiology-visual acuity-color vision-Plate 18
hrr_os_plate19	Neurophysiology-visual acuity-color vision-Plate 19
hrr_os_plate20	Neurophysiology-visual acuity-color vision-Plate 20
hrr_os_plate21	Neurophysiology-visual acuity-color vision-Plate 21
hrr_os_plate22	Neurophysiology-visual acuity-color vision-Plate 22
hrr_os_plate23	Neurophysiology-visual acuity-color vision-Plate 23
hrr_os_plate24	Neurophysiology-visual acuity-color vision-Plate 24
hrr_farbsehtafeln_complete	Neurophysiology-visual acuity-color vision-Complete?
brbn_date	Neuropsychology-Repeatable Battery of Neuropsychological Tests (BRBN)-date
brbn_time	Neuropsychology-Repeatable Battery of Neuropsychological Tests (BRBN)-time
brbn_operator	Neuropsychology-Repeatable Battery of Neuropsychological Tests (BRBN)-operator
brbn_comments	Neuropsychology-Repeatable Battery of Neuropsychological Tests (BRBN)-Remarks
brbn_srt_lts_1	Neuropsychology-Repeatable Battery of Neuropsychological Tests (BRBN)-Selective Reminding Test (SRT)-SRT-LTS 1
brbn_srt_lts_2	Neuropsychology-Repeatable Battery of Neuropsychological Tests (BRBN)-Selective Reminding Test (SRT)-SRT-LTS 2
brbn_srt_lts_3	Neuropsychology-Repeatable Battery of Neuropsychological Tests (BRBN)-Selective Reminding Test (SRT)-SRT-LTS 3
brbn_srt_lts_4	Neuropsychology-Repeatable Battery of Neuropsychological Tests (BRBN)-Selective Reminding Test (SRT)-SRT-LTS 4
brbn_srt_lts_5	Neuropsychology-Repeatable Battery of Neuropsychological Tests (BRBN)-Selective Reminding Test (SRT)-SRT-LTS 5
brbn_srt_lts_6	Neuropsychology-Repeatable Battery of Neuropsychological Tests (BRBN)-Selective Reminding Test (SRT)-SRT-LTS 6
brbn_srtcltr_1	Neuropsychology-Repeatable Battery of Neuropsychological Tests (BRBN)-Selective Reminding Test (SRT)-SRT-CLTR 1
brbn_srt_cltr_2	Neuropsychology-Repeatable Battery of Neuropsychological Tests (BRBN)-Selective Reminding Test (SRT)-SRT-CLTR 2
brbn_srt_cltr_3	Neuropsychology-Repeatable Battery of Neuropsychological Tests (BRBN)-Selective Reminding Test (SRT)-SRT-CLTR 3
brbn_srt_cltr_4	Neuropsychology-Repeatable Battery of Neuropsychological Tests (BRBN)-Selective Reminding Test (SRT)-SRT-CLTR 4
brbn_srt_cltr_5	Neuropsychology-Repeatable Battery of Neuropsychological Tests (BRBN)-Selective Reminding Test (SRT)-SRT-CLTR 5
brbn_srt_cltr_6	Neuropsychology-Repeatable Battery of Neuropsychological Tests (BRBN)-Selective Reminding Test (SRT)-SRT-CLTR 6
brbn_srt_dr	Neuropsychology-Repeatable Battery of Neuropsychological Tests (BRBN)-Selective Reminding Test (SRT)-SRT-DR
brbn_spat_1	Neuropsychology-Repeatable Battery of Neuropsychological Tests (BRBN)-Spatial Recall Test (SPAT)-SPAT 1
brbn_spat_2	Neuropsychology-Repeatable Battery of Neuropsychological Tests (BRBN)-Spatial Recall Test (SPAT)-SPAT 2

Table S1

variable code	Explanation
brbn_spat_3	Neuropsychology-Repeatable Battery of Neuropsychological Tests (BRBN)-Spatial Recall Test (SPAT)-SPAT 3
brbn_spat_dr	Neuropsychology-Repeatable Battery of Neuropsychological Tests (BRBN)-Spatial Recall Test (SPAT)-SPAT-DR
brbn_sdmt	Neuropsychology-Repeatable Battery of Neuropsychological Tests (BRBN)-Symbol-Digit-Modalitiy-Test (SDMT)-SDMT
brbn_pasat3	Neuropsychology-Repeatable Battery of Neuropsychological Tests (BRBN)-Paced Auditory Serial Addition Test (PASAT)-PASAT-3
brbn_pasat2	Neuropsychology-Repeatable Battery of Neuropsychological Tests (BRBN)-Paced Auditory Serial Addition Test (PASAT)-PASAT-2
brbn_wlg_korr	Neuropsychology-Repeatable Battery of Neuropsychological Tests (BRBN)-Word List Generation (WLG)-WLG corr.
brbn_wlg_pers	Neuropsychology-Repeatable Battery of Neuropsychological Tests (BRBN)-Word List Generation (WLG)-WLG pers.
brbn_wlg_intru	Neuropsychology-Repeatable Battery of Neuropsychological Tests (BRBN)-Word List Generation (WLG)-WLG intru.
brbn_neuropsychologische_testbatterie_complete	Neuropsychology-Repeatable Battery of Neuropsychological Tests (BRBN)-Complete?
nicotin_fragebogen_aktivrauchen_timestamp	substance abuse-smoking-active-Survey timestamp
nicotin_1a	substance abuse-smoking-active-1a. Have you smoked regularly for more than 6 months in your life?
nicotin_1b	substance abuse-smoking-active-1b. When did you start smoking regularly? Aged:
nicotin_2a	substance abuse-smoking-active-2a. Do you currently smoke - even if only occasionally?
nicotin_2b_cigarettes	substance abuse-smoking-active-Cigarettes
nicotin_2b_cigars	substance abuse-smoking-active-Cigars, cigarillos, cheroots
nicotin_2b_pipes	substance abuse-smoking-active-Pipes
nicotin_2b_electric	substance abuse-smoking-active-Electric cigarettes
nicotin_2c_cigarettes	substance abuse-smoking-active-Cigarettes
nicotin_2c_cigars	substance abuse-smoking-active-Cigars, cigarillos, cheroots
nicotin_2c_pipes	substance abuse-smoking-active-Pipes
nicotin_2c_electric	substance abuse-smoking-active-Electric cigarettes
nicotin_2d	substance abuse-smoking-active-Aged
nicotin_3_cigarettes	substance abuse-smoking-active-Cigarettes
nicotin_3_cigars	substance abuse-smoking-active-Cigars, cigarillos, cheroots
nicotin_3_pipes	substance abuse-smoking-active-Pipes
nicotin_3_electric	substance abuse-smoking-active-Electric cigarettes
nicotin_fragebogen_aktivrauchen_complete	substance abuse-smoking-active-Complete?
audits_fragebogen_zum_alkoholkonsum_timestamp	substance abuse-alcohol-Survey timestamp
audit_a	substance abuse-alcohol-a) How often do you drink alcohol?
audit_b	substance abuse-alcohol-b) If you drink alcohol in one day, how many alcoholic beverages do you typically drink?
audits_fragebogen_zum_alkoholkonsum_complete	substance abuse-alcohol-Complete?
vas_visuelle_analogskalen_timestamp	Visual analogue scale-Survey timestamp
vas_general	Visual analogue scale-general
vas_fatigue	Visual analogue scale-fatigue
vas_pain	Visual analogue scale-pain
vas_cognition	Visual analogue scale-cognition
vas_visuelle_analogskalen_complete	Visual analogue scale-Complete?
sf36_fragebogen_zum_gesundheitszustand_timestamp	Short Form (36) Health Survey-Survey timestamp
sf36_ghp1	Short Form (36) Health Survey-1. How would you describe your general health?
sf36_hchange1	Short Form (36) Health Survey-2. Compared to last year, how would you describe your current state of health?
sf36_pfi01	Short Form (36) Health Survey-3a. DEMANDING ACTIVITIES, e.g. running fast, lifting heavy objects, engaging in strenuous sports
sf36_pfi02	Short Form (36) Health Survey-3b. MODERATE ACTIVITIES, e.g. Moving a table, vacuuming, bowling, playing golf
sf36_pfi03	Short Form (36) Health Survey-3c. Lifting and carrying shopping bags
sf36_pfi04	Short Form (36) Health Survey-3d. Climb SEVERAL stair landings
sf36_pfi05	Short Form (36) Health Survey-3e. Climb ONE landing
sf36_pfi06	Short Form (36) Health Survey-3f. bend, kneel, stoop
sf36_pfi07	Short Form (36) Health Survey-3g. Walk MORE THAN 1 kilometer on foot
sf36_pfi08	Short Form (36) Health Survey-3h. WALK SEVERAL intersections far
sf36_pfi09	Short Form (36) Health Survey-3i. Go ONE crossroad on foot
sf36_pfi10	Short Form (36) Health Survey-3y. bathe or get dressed
sf36_rolph1	Short Form (36) Health Survey-4a. I couldn't work as long as usual
sf36_rolph2	Short Form (36) Health Survey-4b. I did less than I wanted
sf36_rolph3	Short Form (36) Health Survey-4c. I could only do certain things
sf36_rolph4	Short Form (36) Health Survey-4d. I had difficulty executing
sf36_rolem1	Short Form (36) Health Survey-5a. I couldn't work as long as usual
sf36_rolem2	Short Form (36) Health Survey-5b. I did less than I wanted
sf36_rolem3	Short Form (36) Health Survey-5c. I couldn't work as carefully as usual
sf36_soc1	Short Form (36) Health Survey-6. To what extent have your physical health or emotional problems impaired your normal contact with family members, friends, neighbors or acquaintances in the PAST 4 WEEKS?
sf36_pain1	Short Form (36) Health Survey-7. How bad was your pain in the PAST 4 WEEKS?
sf36_pain2	Short Form (36) Health Survey-8. To what extent has the pain prevented you from doing your daily activities at home and at work in the PAST 4 WEEKS?
sf36_vital1	Short Form (36) Health Survey-9a. ... full swing?
sf36_mhi1	Short Form (36) Health Survey-9b. ... Very Nervous?
sf36_mhi2	Short Form (36) Health Survey-9c. ... so depressed that nothing could cheer you up?
sf36_mhi3	Short Form (36) Health Survey-9d. ... calm and relaxed?
sf36_vital2	Short Form (36) Health Survey-9e. ... full of energy?
sf36_mhi4	Short Form (36) Health Survey-9f. ... discouraged and sad?
sf36_vital3	Short Form (36) Health Survey-9g. ... exhausted?
sf36_mhi5	Short Form (36) Health Survey-9h. ... happy?
sf36_vital4	Short Form (36) Health Survey-9i. ... tired?
sf36_soc2	Short Form (36) Health Survey-10. How often have your physical health or emotional problems impaired your contacts with other people (visits to friends, relatives, etc.) in the PAST 4 WEEKS?
sf36_ghp2	Short Form (36) Health Survey-11a. I seem to get sick a little easier than others.
sf36_ghp3	Short Form (36) Health Survey-11b. I am just as healthy as everyone else I know.
sf36_ghp4	Short Form (36) Health Survey-11c. I expect my health to decline.
sf36_ghp5	Short Form (36) Health Survey-11d. I am in excellent health.
sf36_fragebogen_zum_gesundheitszustand_complete	Short Form (36) Health Survey-Complete?
fss_fatigue_severity_scale_timestamp	Psychometrics-Fatigue Severity Scale (FSS)-Survey timestamp
fss_1	Psychometrics-Fatigue Severity Scale (FSS)-1. My motivation is lower when I am fatigued.
fss_2	Psychometrics-Fatigue Severity Scale (FSS)-2. Exercise brings on my fatigue.
fss_3	Psychometrics-Fatigue Severity Scale (FSS)-3. I am easily fatigued.
fss_4	Psychometrics-Fatigue Severity Scale (FSS)-4. Fatigue interferes with my physical functioning.
fss_5	Psychometrics-Fatigue Severity Scale (FSS)-5. Fatigue causes frequent problems for me.
fss_6	Psychometrics-Fatigue Severity Scale (FSS)-6. My fatigue prevents sustained physical functioning.
fss_7	Psychometrics-Fatigue Severity Scale (FSS)-7. Fatigue interferes with carrying out certain duties and responsibilities.
fss_8	Psychometrics-Fatigue Severity Scale (FSS)-8. Fatigue is among my three most disabling symptoms.
fss_9	Psychometrics-Fatigue Severity Scale (FSS)-9. Fatigue interferes with my work, family, or social life.
fss_fatigue_severity_scale_complete	Psychometrics-Fatigue Severity Scale (FSS)-Complete?

Table S1

variable code	Explanation
walk12_gehfhigkeit_timestamp	Psychometrics-Twelve Item MS Walking Scale (MSWS-12)-Survey timestamp
walk12_01	Psychometrics-Twelve Item MS Walking Scale (MSWS-12)-1. Limited your ability to walk?
walk12_02	Psychometrics-Twelve Item MS Walking Scale (MSWS-12)-2. Limited your ability to run?
walk12_03	Psychometrics-Twelve Item MS Walking Scale (MSWS-12)-3. Limited your ability to climb up and down stairs?
walk12_04	Psychometrics-Twelve Item MS Walking Scale (MSWS-12)-4. Made standing when doing things more difficult?
walk12_05	Psychometrics-Twelve Item MS Walking Scale (MSWS-12)-5. Limited your balance when standing or walking?
walk12_06	Psychometrics-Twelve Item MS Walking Scale (MSWS-12)-6. Limited how far you are able to walk?
walk12_07	Psychometrics-Twelve Item MS Walking Scale (MSWS-12)-7. Increased the effort needed for you to walk?
walk12_08	Psychometrics-Twelve Item MS Walking Scale (MSWS-12)-8. Made it necessary for you to use support when walking indoors (eg holding on to furniture, using a stick, etc.)?
walk12_09	Psychometrics-Twelve Item MS Walking Scale (MSWS-12)-9. Made it necessary for you to use support when walking outdoors (eg using a stick, a frame, etc.)?
walk12_10	Psychometrics-Twelve Item MS Walking Scale (MSWS-12)-10. Slowed down your walking?
walk12_11	Psychometrics-Twelve Item MS Walking Scale (MSWS-12)-11. Affected how smoothly you walk?
walk12_12	Psychometrics-Twelve Item MS Walking Scale (MSWS-12)-12. Made you concentrate on your walking?
walk12_gehfhigkeit_complete	Psychometrics-Twelve Item MS Walking Scale (MSWS-12)-Complete?
fsmc_fatigue_skala_fr_motorik_und_kognition_timestamp	Psychometrics-Fatigue Scale for Motor and Cognitive Functions (FSMC)-Survey timestamp
fsmc_1	Psychometrics-Fatigue Scale for Motor and Cognitive Functions (FSMC)-1. When I concentrate for a long time, I get exhausted sooner than other people of my age.
fsmc_2	Psychometrics-Fatigue Scale for Motor and Cognitive Functions (FSMC)-2. When I am experiencing episodes of exhaustion, my movements become noticeably clumsier and less coordinated.
fsmc_3	Psychometrics-Fatigue Scale for Motor and Cognitive Functions (FSMC)-3. Because of my episodes of exhaustion, I now need more frequent and/or longer rests during physical activity than I used to.
fsmc_4	Psychometrics-Fatigue Scale for Motor and Cognitive Functions (FSMC)-4. When I am experiencing episodes of exhaustion, I am incapable of making decisions.
fsmc_5	Psychometrics-Fatigue Scale for Motor and Cognitive Functions (FSMC)-5. When faced with stressful situations, I now find that I get physically exhausted quicker than I used to.
fsmc_6	Psychometrics-Fatigue Scale for Motor and Cognitive Functions (FSMC)-6. Because of my episodes of exhaustion, I now have considerably less social contact than I have considerably less social contact than I used to.
fsmc_7	Psychometrics-Fatigue Scale for Motor and Cognitive Functions (FSMC)-7. Because of my episodes of exhaustion, I now find it more difficult to learn new things than I used to.
fsmc_8	Psychometrics-Fatigue Scale for Motor and Cognitive Functions (FSMC)-8. The demands of my work exhaust me mentally more quickly than they used to.
fsmc_9	Psychometrics-Fatigue Scale for Motor and Cognitive Functions (FSMC)-9. I feel the episodes of exhaustion particularly strongly in my muscles.
fsmc_10	Psychometrics-Fatigue Scale for Motor and Cognitive Functions (FSMC)-20. During episodes of exhaustion, I am noticeably more forgetful.
fsmc_11	Psychometrics-Fatigue Scale for Motor and Cognitive Functions (FSMC)-19. When it is hot, my main feeling is one of extreme physical weakness and lack of energy.
fsmc_12	Psychometrics-Fatigue Scale for Motor and Cognitive Functions (FSMC)-10. I no longer have the stamina for long periods of physical activity that I used to have.
fsmc_13	Psychometrics-Fatigue Scale for Motor and Cognitive Functions (FSMC)-11. My powers of concentration decrease considerably when I'm under stress.
fsmc_14	Psychometrics-Fatigue Scale for Motor and Cognitive Functions (FSMC)-12. When I am experiencing episodes of exhaustion, I am less motivated than others to start activities that involve physical effort.
fsmc_15	Psychometrics-Fatigue Scale for Motor and Cognitive Functions (FSMC)-13. My thinking gets increasingly slow when it is hot.
fsmc_16	Psychometrics-Fatigue Scale for Motor and Cognitive Functions (FSMC)-14. When I am experiencing an episode of exhaustion, my movements become noticeably slower.
fsmc_17	Psychometrics-Fatigue Scale for Motor and Cognitive Functions (FSMC)-15. Because of my episodes of exhaustion, I now feel less like doing things which require concentration.
fsmc_18	Psychometrics-Fatigue Scale for Motor and Cognitive Functions (FSMC)-16. When an episode of exhaustion comes on, I am simply no longer able to react quickly.
fsmc_19	Psychometrics-Fatigue Scale for Motor and Cognitive Functions (FSMC)-17. When I am experiencing episodes of exhaustion, certain words simply escape me.
fsmc_20	Psychometrics-Fatigue Scale for Motor and Cognitive Functions (FSMC)-18. When I am experiencing episodes of exhaustion, I lose concentration considerably quicker than I used to.
fsmc_fatigue_skala_fr_motorik_und_kognition_complete	Psychometrics-Fatigue Scale for Motor and Cognitive Functions (FSMC)-Complete?
mfis_modifizierter_fatigue_impact_scale_timestamp	Psychometrics-Modified Fatigue Impact Scale (MFIS)-Survey timestamp
mfis_01	Psychometrics-Modified Fatigue Impact Scale (MFIS)-1. I have been less alert. 0 1 2 3 4
mfis_02	Psychometrics-Modified Fatigue Impact Scale (MFIS)-2. I have had difficulty paying attention for long periods of time.
mfis_03	Psychometrics-Modified Fatigue Impact Scale (MFIS)-3. I have been unable to think clearly.
mfis_04	Psychometrics-Modified Fatigue Impact Scale (MFIS)-4. I have been clumsy and uncoordinated.
mfis_05	Psychometrics-Modified Fatigue Impact Scale (MFIS)-5. I have been forgetful. 0 1 2 3 4
mfis_06	Psychometrics-Modified Fatigue Impact Scale (MFIS)-6. I have had to pace myself in my physical activities.
mfis_07	Psychometrics-Modified Fatigue Impact Scale (MFIS)-7. I have been less motivated to do anything that requires physical effort.
mfis_08	Psychometrics-Modified Fatigue Impact Scale (MFIS)-8. I have been less motivated to participate in social activities.
mfis_09	Psychometrics-Modified Fatigue Impact Scale (MFIS)-9. I have been limited in my ability to do things away from home.
mfis_10	Psychometrics-Modified Fatigue Impact Scale (MFIS)-10. I have had trouble maintaining physical effort for long periods.
mfis_11	Psychometrics-Modified Fatigue Impact Scale (MFIS)-11. I have had difficulty making decisions.
mfis_12	Psychometrics-Modified Fatigue Impact Scale (MFIS)-12. I have been less motivated to do anything that requires thinking.
mfis_13	Psychometrics-Modified Fatigue Impact Scale (MFIS)-13. my muscles have felt weak.
mfis_14	Psychometrics-Modified Fatigue Impact Scale (MFIS)-14. I have been physically uncomfortable.
mfis_15	Psychometrics-Modified Fatigue Impact Scale (MFIS)-15. I have had trouble finishing tasks that require thinking.
mfis_16	Psychometrics-Modified Fatigue Impact Scale (MFIS)-16. I have had difficulty organizing my thoughts when doing things at home or at work.
mfis_17	Psychometrics-Modified Fatigue Impact Scale (MFIS)-17. I have been less able to complete tasks that require physical effort.
mfis_18	Psychometrics-Modified Fatigue Impact Scale (MFIS)-18. my thinking has been slowed down.
mfis_19	Psychometrics-Modified Fatigue Impact Scale (MFIS)-19. I have had trouble concentrating.
mfis_20	Psychometrics-Modified Fatigue Impact Scale (MFIS)-20. I have limited my physical activities.
mfis_21	Psychometrics-Modified Fatigue Impact Scale (MFIS)-21. I have needed to rest more often or for longer periods
mfis_modifizierter_fatigue_impact_scale_complete	Psychometrics-Modified Fatigue Impact Scale (MFIS)-Complete?
bdiia_beck_depressions_inventar_ia_timestamp	Psychometrics-Beck's depressions inventar (BDI)-Survey timestamp
bdi1a_a	Psychometrics-Beck's depressions inventar (BDI)-Question 1
bdi1a_b	Psychometrics-Beck's depressions inventar (BDI)-Question 2
bdi1a_c	Psychometrics-Beck's depressions inventar (BDI)-Question 3
bdi1a_d	Psychometrics-Beck's depressions inventar (BDI)-Question 4
bdi1a_e	Psychometrics-Beck's depressions inventar (BDI)-Question 5
bdi1a_f	Psychometrics-Beck's depressions inventar (BDI)-Question 6
bdi1a_g	Psychometrics-Beck's depressions inventar (BDI)-Question 7
bdi1a_h	Psychometrics-Beck's depressions inventar (BDI)-Question 8
bdi1a_i	Psychometrics-Beck's depressions inventar (BDI)-Question 9

Table S1

variable code	Explanation
bdi1a_j	Psychometrics-Beck's depressions inventar (BDI)-Question 10
bdi1a_k	Psychometrics-Beck's depressions inventar (BDI)-Question 11
bdi1a_l	Psychometrics-Beck's depressions inventar (BDI)-Question 12
bdi1a_m	Psychometrics-Beck's depressions inventar (BDI)-Question 13
bdi1a_n	Psychometrics-Beck's depressions inventar (BDI)-Question 14
bdi1a_o	Psychometrics-Beck's depressions inventar (BDI)-Question 15
bdi1a_p	Psychometrics-Beck's depressions inventar (BDI)-Question 16
bdi1a_q	Psychometrics-Beck's depressions inventar (BDI)-Question 17
bdi1a_r	Psychometrics-Beck's depressions inventar (BDI)-Question 18
bdi1a_s	Psychometrics-Beck's depressions inventar (BDI)-Question 19
bdi1a_s_control	Psychometrics-Beck's depressions inventar (BDI)-Question 20
bdi1a_t	Psychometrics-Beck's depressions inventar (BDI)-Question 21
bdi1a_u	Psychometrics-Beck's depressions inventar (BDI)-Question 22
bdiia_beck_depressions_inventar_ia_complete	Psychometrics-Beck's depressions inventar (BDI)-Question 23
bdiii_beck_depressions_inventar_timestamp	Psychometrics-Beck's depressions inventar (BDI)-Question 24
mohr_a	Psychometrics-Beck's depressions inventar (BDI)-a) In the past 2 weeks, have you felt depressed or oppressed most days and most of the day?
mohr_b	Psychometrics-Beck's depressions inventar (BDI)-b) In the last 2 weeks, have you had no interest in doing anything or have you not enjoyed doing things that you otherwise enjoyed?
bdi2_1	Psychometrics-Beck's depressions inventar (BDI)-1.) Sadness
bdi2_2	Psychometrics-Beck's depressions inventar (BDI)-2.) Pessimism
bdi2_3	Psychometrics-Beck's depressions inventar (BDI)-3.) Feelings of failure
bdi2_4	Psychometrics-Beck's depressions inventar (BDI)-4. Loss of joy
bdi2_5	Psychometrics-Beck's depressions inventar (BDI)-5.) Feelings of guilt
bdi2_6	Psychometrics-Beck's depressions inventar (BDI)-6.) Feelings of punishment
bdi2_7	Psychometrics-Beck's depressions inventar (BDI)-7.) Self-rejection
bdi2_8	Psychometrics-Beck's depressions inventar (BDI)-8.) Self-reproach
bdi2_9	Psychometrics-Beck's depressions inventar (BDI)-9. Thoughts of suicide
bdi2_10	Psychometrics-Beck's depressions inventar (BDI)-10.) Crying
bdi2_11	Psychometrics-Beck's depressions inventar (BDI)-11.) Restlessness
bdi2_12	Psychometrics-Beck's depressions inventar (BDI)-12.) Loss of interest
bdi2_13	Psychometrics-Beck's depressions inventar (BDI)-13.) Incapacity to make decisions
bdi2_14	Psychometrics-Beck's depressions inventar (BDI)-14.) Worthlessness
bdi2_15	Psychometrics-Beck's depressions inventar (BDI)-15.) Loss of energy
bdi2_16	Psychometrics-Beck's depressions inventar (BDI)-16.) Changes in sleeping habits
bdi2_17	Psychometrics-Beck's depressions inventar (BDI)-17.) Irritability
bdi2_18	Psychometrics-Beck's depressions inventar (BDI)-18.) Changes in appetite
bdi2_19	Psychometrics-Beck's depressions inventar (BDI)-19. Difficulty concentrating
bdi2_20	Psychometrics-Beck's depressions inventar (BDI)-20.) Fatigue or exhaustion
bdi2_21	Psychometrics-Beck's depressions inventar (BDI)-21.) Loss of sexual interest
bdiii_beck_depressions_inventar_complete	Psychometrics-Beck's depressions inventar (BDI)-Complete?
mpq_mcgill_pain_questionnaire_timestamp	Psychometrics-McGill pain questionnaire-Survey timestamp
mcgill_0	Psychometrics-McGill pain questionnaire-
mcgill_1	Psychometrics-McGill pain questionnaire-Group 1 Flickering, Pulsing, Quivering, Throbbing, Beating, Pounding
mcgill_2	Psychometrics-McGill pain questionnaire-Group 2 Jumping, Flashing, Shooting
mcgill_3	Psychometrics-McGill pain questionnaire-Group 3 Pricking, Boring, Drilling, Stabbing
mcgill_4	Psychometrics-McGill pain questionnaire-Group 4 Sharp, Cutting, Lacerating
mcgill_5	Psychometrics-McGill pain questionnaire-Group 5 Pinching, Pressing, Gnawing, Cramping, Crushing
mcgill_6	Psychometrics-McGill pain questionnaire-Group 6 Tugging, Pulling, Wrenching
mcgill_7	Psychometrics-McGill pain questionnaire-Group 7 Hot, Burning, Scalding, Searing
mcgill_8	Psychometrics-McGill pain questionnaire-Group 8 Tingling, Itchy, Smarting, Stinging
mcgill_9	Psychometrics-McGill pain questionnaire-Group 9 Dull, Sore, Hurting, Aching, Heavy
mcgill_10	Psychometrics-McGill pain questionnaire-Group 10 Tender, Taut (tight), Rasping, Splitting
mcgill_11	Psychometrics-McGill pain questionnaire-Group 11 Tiring, Exhausting
mcgill_12	Psychometrics-McGill pain questionnaire-Group 12 Sickening, Suffocating
mcgill_13	Psychometrics-McGill pain questionnaire-Group 13 Fearful, Frightful, Terrifying
mcgill_14	Psychometrics-McGill pain questionnaire-Group 14 Punishing, Grueling, Cruel, Vicious, Killing
mcgill_15	Psychometrics-McGill pain questionnaire-Group 15 Wretched, Blinding
mcgill_16	Psychometrics-McGill pain questionnaire-Group 16 Annoying, Troublesome, Miserable, Intense, Unbearable
mcgill_17	Psychometrics-McGill pain questionnaire-Group 17 Spreading, Radiating, Penetrating, Piercing
mcgill_18	Psychometrics-McGill pain questionnaire-Group 18 Tight, Numb, Squeezing, Drawing, Tearing
mcgill_19	Psychometrics-McGill pain questionnaire-Group 19 Cool, Cold, Freezing
mcgill_20	Psychometrics-McGill pain questionnaire-Group 20 Nagging, Nauseating, Agonizing, Dreadful, Torturing
mcgill_21	Psychometrics-McGill pain questionnaire-
mpq_mcgill_pain_questionnaire_complete	Psychometrics-McGill pain questionnaire-Complete?
pdq_pain_detect_schmerzfragebogen_timestamp	Psychometrics-painDETECT (PDQ) questionnaire for neuropathic pain detection-Survey timestamp
pdq_1	Psychometrics-painDETECT (PDQ) questionnaire for neuropathic pain detection-Do you suffer from a burning sensation in the areas mentioned?
pdq_2	Psychometrics-painDETECT (PDQ) questionnaire for neuropathic pain detection-Do you have a tingling or tingling sensation (such as pins and needles, electrical tingling) in the areas of your pain
pdq_3	Psychometrics-painDETECT (PDQ) questionnaire for neuropathic pain detection-Is light touch (clothing, duvet) painful in these areas?
pdq_4	Psychometrics-painDETECT (PDQ) questionnaire for neuropathic pain detection-Have flash-like, electrifying pain attacks in the areas of your pain
pdq_4dd___0	Psychometrics-painDETECT (PDQ) questionnaire for neuropathic pain detection-Is cold or heat (bath water) occasionally painful in these areas?
pdq_4dd___1	Psychometrics-painDETECT (PDQ) questionnaire for neuropathic pain detection-Is cold or heat (bath water) occasionally painful in these areas?
pdq_4dd___2	Psychometrics-painDETECT (PDQ) questionnaire for neuropathic pain detection-Is cold or heat (bath water) occasionally painful in these areas?
pdq_4dd___3	Psychometrics-painDETECT (PDQ) questionnaire for neuropathic pain detection-Is cold or heat (bath water) occasionally painful in these areas?
pdq_4dd___4	Psychometrics-painDETECT (PDQ) questionnaire for neuropathic pain detection-Is cold or heat (bath water) occasionally painful in these areas?
pdq_4dd___5	Psychometrics-painDETECT (PDQ) questionnaire for neuropathic pain detection-Is cold or heat (bath water) occasionally painful in these areas?
pdq_4dd___6	Psychometrics-painDETECT (PDQ) questionnaire for neuropathic pain detection-Is cold or heat (bath water) occasionally painful in these areas?
pdq_5a	Psychometrics-painDETECT (PDQ) questionnaire for neuropathic pain detection-Do you suffer from numbness in the areas you mentioned?
pdq_6a	Psychometrics-painDETECT (PDQ) questionnaire for neuropathic pain detection-Releases light pressure e.g. feel pain with your fingers in this area?
pdq_6b	Psychometrics-painDETECT (PDQ) questionnaire for neuropathic pain detection-Releases light pressure e.g. feel pain with your fingers in this area?
pdq_6c	Psychometrics-painDETECT (PDQ) questionnaire for neuropathic pain detection-Releases light pressure e.g. feel pain with your fingers in this area?
pdq_6d	Psychometrics-painDETECT (PDQ) questionnaire for neuropathic pain detection-Releases light pressure e.g. feel pain with your fingers in this area?
pdq_6e	Psychometrics-painDETECT (PDQ) questionnaire for neuropathic pain detection-Releases light pressure e.g. feel pain with your fingers in this area?
pdq_6f	Psychometrics-painDETECT (PDQ) questionnaire for neuropathic pain detection-Releases light pressure e.g. feel pain with your fingers in this area?

Table S1

variable code	Explanation
pdq_6g	Psychometrics-painDETECT (PDQ) questionnaire for neuropathic pain detection-Releases light pressure e.g. feel pain with your fingers in this area?
pdq_pain_detect_schmerzfragebogen_complete	Psychometrics-painDETECT (PDQ) questionnaire for neuropathic pain detection-Complete?
brief_pain_inventory_timestamp	Psychometrics-brief pain inventory (BPI)-Survey timestamp
bpi_item01	Psychometrics-brief pain inventory (BPI)-1.) Most of us have pain from time to time (e.g. headache, toothache, sprains). Did you have any other pain than this everyday pain today?
bpi_item02_file	Psychometrics-brief pain inventory (BPI)-Here the edited picture is uploaded by an employee.
bpi_item03	Psychometrics-brief pain inventory (BPI)-3.) Please mark the number that describes your worst pain in the last 24 hours:
bpi_item04	Psychometrics-brief pain inventory (BPI)-4.) Please mark the number that describes your least pain in the last 24 hours:
bpi_item05	Psychometrics-brief pain inventory (BPI)-5.) Please mark the number that describes your average pain over the last 24 hours:
bpi_item06	Psychometrics-brief pain inventory (BPI)-6.) Please mark the number that says what pain you are in at this moment:
bpi_item07_yn	Psychometrics-brief pain inventory (BPI)-7.) Are you receiving medication or other therapy for your pain?
bpi_item07	Psychometrics-brief pain inventory (BPI)-7.) Please specify: Which treatments or medications do you receive for your pain?
bpi_item08	Psychometrics-brief pain inventory (BPI)-8.) Please think about the past 24 hours. How much pain relief have you experienced from treatments or medication? Please mark the percentage that best shows the pain relief.
bpi_item09	Psychometrics-brief pain inventory (BPI)-9.) General activity
bpi_item10	Psychometrics-brief pain inventory (BPI)-10.) Mood
bpi_item11	Psychometrics-brief pain inventory (BPI)-11.) Walking ability
bpi_item12	Psychometrics-brief pain inventory (BPI)-12.) Normal work (both outside the home and housework)
bpi_item13	Psychometrics-brief pain inventory (BPI)-13.) Relationship with other people
bpi_item14	Psychometrics-brief pain inventory (BPI)-14.) Sleep
bpi_item15	Psychometrics-brief pain inventory (BPI)-15.) Joy of life
bpi_comments	Psychometrics-brief pain inventory (BPI)-Comments:
brief_pain_inventory_complete	Psychometrics-brief pain inventory (BPI)-Complete?
psqi_schlafqualittsfragebogen_timestamp	Psychometrics-quality of sleep (PSQI)-Survey timestamp
psqi_1	Psychometrics-quality of sleep (PSQI)-1. During the past four weeks, when have you usually gone to bed at night?
psqi_2	Psychometrics-quality of sleep (PSQI)-2. During the past four weeks, how long has it usually taken you to fall asleep at night?
psqi_3	Psychometrics-quality of sleep (PSQI)-3. In the past four weeks, when did you usually get up in the morning?
psqi_4	Psychometrics-quality of sleep (PSQI)-4. How many hours have you actually slept each night during the past four weeks? (This doesn't have to match the number of hours you spent in bed.)
psqi_5a	Psychometrics-quality of sleep (PSQI)-a) ... because you couldn't fall asleep within 30 minutes?
psqi_5b	Psychometrics-quality of sleep (PSQI)-b) ... because you woke up in the middle of the night or early in the morning?
psqi_5c	Psychometrics-quality of sleep (PSQI)-c) ... because you had to get up to go to the toilet?
psqi_5d	Psychometrics-quality of sleep (PSQI)-d) ... because you had difficulty breathing?
psqi_5e	Psychometrics-quality of sleep (PSQI)-e) ... because you coughed or snored loudly?
psqi_5f	Psychometrics-quality of sleep (PSQI)-f) ... because you were too cold?
psqi_5g	Psychometrics-quality of sleep (PSQI)-g) ... because you felt too warm?
psqi_5h	Psychometrics-quality of sleep (PSQI)-h) ... because you had a bad dream?
psqi_5i	Psychometrics-quality of sleep (PSQI)-i) ... because you were in pain?
psqi_5j	Psychometrics-quality of sleep (PSQI)-j) ... for other reasons?
psqi_5j_comments	Psychometrics-quality of sleep (PSQI)-Please describe:
psqi_6	Psychometrics-quality of sleep (PSQI)-6. Overall, how would you rate the quality of your sleep over the past four weeks?
psqi_7	Psychometrics-quality of sleep (PSQI)-7. How often have you taken sleeping pills (doctor-prescribed or over-the-counter) in the past four weeks?
psqi_8	Psychometrics-quality of sleep (PSQI)-8. How often have you had difficulty staying awake in the last four weeks, such as driving, eating, or at social gatherings?
psqi_9	Psychometrics-quality of sleep (PSQI)-9. During the past four weeks, have you had problems doing normal day-to-day tasks with enough momentum?
psqi_10	Psychometrics-quality of sleep (PSQI)-10. Do you sleep alone in your room?
psqi_11a	Psychometrics-quality of sleep (PSQI)-a) Loud snoring
psqi_11b	Psychometrics-quality of sleep (PSQI)-b) Long pauses in breathing during sleep
psqi_11c	Psychometrics-quality of sleep (PSQI)-c) twitching or jerking movements of the legs while sleeping
psqi_11d	Psychometrics-quality of sleep (PSQI)-d) Nightly P
psqi_11e	Psychometrics-quality of sleep (PSQI)-comments
psqi_schlafqualittsfragebogen_complete	Psychometrics-quality of sleep (PSQI)-Complete?
neivfq_fragebogen_zur_sehkraft_timestamp	Neurophysiology-visual acuity questionnaire (NEIVFQ)-Survey timestamp
vfq_01	Neurophysiology-visual acuity questionnaire (NEIVFQ)-Item 1
vfq_02	Neurophysiology-visual acuity questionnaire (NEIVFQ)-Item 2
vfq_03	Neurophysiology-visual acuity questionnaire (NEIVFQ)-Item 3
vfq_04	Neurophysiology-visual acuity questionnaire (NEIVFQ)-Item 4
vfq_05	Neurophysiology-visual acuity questionnaire (NEIVFQ)-Item 5
vfq_06	Neurophysiology-visual acuity questionnaire (NEIVFQ)-Item 6
vfq_07	Neurophysiology-visual acuity questionnaire (NEIVFQ)-Item 7
vfq_08	Neurophysiology-visual acuity questionnaire (NEIVFQ)-Item 8
vfq_09	Neurophysiology-visual acuity questionnaire (NEIVFQ)-Item 9
vfq_10	Neurophysiology-visual acuity questionnaire (NEIVFQ)-Item 10
vfq_11	Neurophysiology-visual acuity questionnaire (NEIVFQ)-Item 11
vfq_12	Neurophysiology-visual acuity questionnaire (NEIVFQ)-Item 12
vfq_13	Neurophysiology-visual acuity questionnaire (NEIVFQ)-Item 13
vfq_14	Neurophysiology-visual acuity questionnaire (NEIVFQ)-Item 14
vfq_15	Neurophysiology-visual acuity questionnaire (NEIVFQ)-Item 15
vfq_15a	Neurophysiology-visual acuity questionnaire (NEIVFQ)-Item 16
vfq_15b	Neurophysiology-visual acuity questionnaire (NEIVFQ)-Item 17
vfq_15c	Neurophysiology-visual acuity questionnaire (NEIVFQ)-Item 18
vfq_16	Neurophysiology-visual acuity questionnaire (NEIVFQ)-Item 19
vfq_17	Neurophysiology-visual acuity questionnaire (NEIVFQ)-Item 20
vfq_18	Neurophysiology-visual acuity questionnaire (NEIVFQ)-Item 21
vfq_19	Neurophysiology-visual acuity questionnaire (NEIVFQ)-Item 22
vfq_20	Neurophysiology-visual acuity questionnaire (NEIVFQ)-Item 23
vfq_21	Neurophysiology-visual acuity questionnaire (NEIVFQ)-Item 24
vfq_22	Neurophysiology-visual acuity questionnaire (NEIVFQ)-Item 25
vfq_23	Neurophysiology-visual acuity questionnaire (NEIVFQ)-Item 26
vfq_24	Neurophysiology-visual acuity questionnaire (NEIVFQ)-Item 27
vfq_25	Neurophysiology-visual acuity questionnaire (NEIVFQ)-Item 28
vfq_a1	Neurophysiology-visual acuity questionnaire (NEIVFQ)-Item a1
vfq_a2	Neurophysiology-visual acuity questionnaire (NEIVFQ)-Item a2
vfq_a3	Neurophysiology-visual acuity questionnaire (NEIVFQ)-Item a3
vfq_a4	Neurophysiology-visual acuity questionnaire (NEIVFQ)-Item a4
vfq_a5	Neurophysiology-visual acuity questionnaire (NEIVFQ)-Item a5
vfq_a6	Neurophysiology-visual acuity questionnaire (NEIVFQ)-Item a6
vfq_a7	Neurophysiology-visual acuity questionnaire (NEIVFQ)-Item a7
vfq_a8	Neurophysiology-visual acuity questionnaire (NEIVFQ)-Item a8
vfq_a9	Neurophysiology-visual acuity questionnaire (NEIVFQ)-Item a9

Table S1

variable code	Explanation
vfq_a10	Neurophysiology-visual acuity questionnaire (NEIVFQ)-Item a10
vfq_a11a	Neurophysiology-visual acuity questionnaire (NEIVFQ)-Item a11a
vfq_a11b	Neurophysiology-visual acuity questionnaire (NEIVFQ)-Item a11b
vfq_a12	Neurophysiology-visual acuity questionnaire (NEIVFQ)-Item a12
vfq_a13	Neurophysiology-visual acuity questionnaire (NEIVFQ)-Item a13
neivfq_fragebogen_zur_sehkraft_complete	Neurophysiology-visual acuity questionnaire (NEIVFQ)-Complete?
vfqnos_neuroophthalmologische_ergnzung_timestamp	Neurophysiology-neuro-ophthalmologic addition (VFQNOS)-Survey timestamp
nos_01	Neurophysiology-neuro-ophthalmologic addition (VFQNOS)-Item 1
nos_02	Neurophysiology-neuro-ophthalmologic addition (VFQNOS)-Item 2
nos_03	Neurophysiology-neuro-ophthalmologic addition (VFQNOS)-Item 3
nos_04	Neurophysiology-neuro-ophthalmologic addition (VFQNOS)-Item 4
nos_05	Neurophysiology-neuro-ophthalmologic addition (VFQNOS)-Item 5
nos_06	Neurophysiology-neuro-ophthalmologic addition (VFQNOS)-Item 6
nos_07	Neurophysiology-neuro-ophthalmologic addition (VFQNOS)-Item 7
nos_08	Neurophysiology-neuro-ophthalmologic addition (VFQNOS)-Item 8
nos_09	Neurophysiology-neuro-ophthalmologic addition (VFQNOS)-Item 9
nos_10	Neurophysiology-neuro-ophthalmologic addition (VFQNOS)-Item 10
nos_a1	Neurophysiology-neuro-ophthalmologic addition (VFQNOS)-Item a1
nos_a2	Neurophysiology-neuro-ophthalmologic addition (VFQNOS)-Item a2
nos_a3	Neurophysiology-neuro-ophthalmologic addition (VFQNOS)-Item a3
vfqnos_neuroophthalmologische_ergnzung_complete	Neurophysiology-neuro-ophthalmologic addition (VFQNOS)-Complete?
dropout_reason	general patient characteristic-dropout-reason for dropout
dropout_comments	general patient characteristic-dropout-comments
drop_out_complete	general patient characteristic-dropout-Complete?

Table S2. Demographic data, disease characteristics and autoantibody status

		general characteristics					education. job situation and disability			substance use and abuse	
	number of patients (N)	mean sex; percentage female (%)	mean body height (cm)	mean body weight (kg)	handedness; percentage left-handed (%)	ethnicity; non-caucasian (%)	mean maximal acquired education level (AU)	job situation; percentage employed (%)	official approved disability (%)	percentage smoking (%)	percentage of alcohol consumption (%)
anti-AQP4-IgG+ and anti-MOG-IgG+: double AQP4+MOG-IgG positive	1	100	162	65	0	100	0	0	0	100	0
anti-AQP4-IgG+ and anti-MOG-IgG-: single AQP4-IgG positive	14	93	167	74	21	0	3	36	68	57	67
anti-AQP4-IgG- and anti-MOG-IgG+: single MOG-IgG positive	17	59	174	76	0	0	3	82	26	77	77
anti-AQP4-IgG- and anti-MOG-IgG-: no autoantibody	10	78	169	76	13	0	3	50	14	60	70
anti-AQP4-IgG+	34	91	166	70	12	0	3	47	53	48	62
anti-AQP4-IgG-	29	64	172	76	7	0	3	70	24	68	72

	autoantibodies at first visit. baseline						autoantibodies at first relapse		autoantibodies at second relapse	
	percentage anti-AQP4-IgG positive (%)	percentage anti-MOG-IgG positive (%)	anti-AQP4-IgG titer (AU)	anti-MOG-IgG titer (AU)	anti-MOG-IgM titer (AU)	anti-MOG-IgA titer (AU)	anti-AQP4-IgG titer (AU)	anti-MOG-IgG titer (AU)	anti-AQP4-IgG titer (AU)	anti-MOG-IgG titer (AU)
anti-AQP4-IgG+ and anti-MOG-IgG+: double AQP4+MOG-IgG positive	100	100	2.264	n.a.	0	0	0	0	0	0
anti-AQP4-IgG+ and anti-MOG-IgG-: single AQP4-IgG positive	100	0	0.067	0	0	0	0.1	0	0.048	0
anti-AQP4-IgG- and anti-MOG-IgG+: single MOG-IgG positive	0	100	0	0.111	0.005	0	0	0.096	0	0.032
anti-AQP4-IgG- and anti-MOG-IgG-: no autoantibody	0	0	0	0	0	0.005	0	0	0	0
anti-AQP4-IgG+	100	7	0.259	0	0	0	0.208	0	0.163	0
anti-AQP4-IgG-	0	63	0	0.066	0.003	0.002	0	0.069	0	0.016

	autoimmune disorders					metabolic diseases		cancer		other pre-existing conditions	
	diagnosis thyroiditis (N)	diagnosis systemic lupus erythematosus (SLE. N)	diagnosis myasthenia gravis (N)	diagnosis Sjögren syndrome (N)	diagnosis of other immune disorder (N)	diagnosis diabetes (N)	diaagnosis hypertension (N)	diagnosis of a malignome (N)	probable paraneoplastic disease as cause of NMO (N)	diagnosis traumatic injury (N)	diagnosis of any other disorder (N)
anti-AQP4-IgG+ and anti-MOG-IgG+: double AQP4+MOG-IgG positive	0	0	0	0	0	0	0	0	0	0	1
anti-AQP4-IgG+ and anti-MOG-IgG-: single AQP4-IgG positive	2	2	2	1	1	1	4	0	0	1	6
anti-AQP4-IgG- and anti-MOG-IgG+: single MOG-IgG positive	1	0	0	0	0	0	4	0	0	2	8
anti-AQP4-IgG- and anti-MOG-IgG-: no autoantibody	0	0	0	0	1	1	3	2	0	2	6
anti-AQP4-IgG+	4	5	2	2	3	1	7	0	0	1	16
anti-AQP4-IgG-	1	0	0	0	1	1	8	3	0	4	14

	mean age of NMO onset			mean age at therapy		
	mean age at symptom onset (years)	mean age at first diagnosis (years)	mean age at first visit date (years)	mean age at start of current therapy (years)	mean age at start of first therapy (years)	mean age at stop of current therapy (years)
anti-AQP4-IgG+ and anti-MOG-IgG+: double AQP4+MOG-IgG positive	34	35	41	40	39	40
anti-AQP4-IgG+ and anti-MOG-IgG-: single AQP4-IgG positive	45	48	56	51	46	48
anti-AQP4-IgG- and anti-MOG-IgG+: single MOG-IgG positive	38	43	45	41	42	43
anti-AQP4-IgG- and anti-MOG-IgG-: no autoantibody	51	53	57	48	53	56
anti-AQP4-IgG+	42	43	49	45	44	46
anti-AQP4-IgG-	42	47	49	44	44	45

	information of relapses				relapse treatment and success					
	mean relapse count (N)	mean age at relapse 1 (years)	mean age at relapse 2 (years)	mean age at relapse 3 (years)	percentage treated with rituximab (%)	percentage treated with Azathioprin (%)	percentage treated with Mycophenolat-mofetil (%)	percentage of therapy success during relapse 1	percentage of therapy success during relapse 2	percentage of therapy success during relapse 3
anti-AQP4-IgG+ and anti-MOG-IgG+: double AQP4+MOG-IgG positive	3	34	36	40	0	0	0	100	100	100
anti-AQP4-IgG+ and anti-MOG-IgG-: single AQP4-IgG positive	2.57	46	52	53	50	7	7	57	43	50
anti-AQP4-IgG- and anti-MOG-IgG+: single MOG-IgG positive	2.88	40	39	35	47	6	6	59	41	24
anti-AQP4-IgG- and anti-MOG-IgG-: no autoantibody	2.70	51	46	38	30	20	10	90	60	20
anti-AQP4-IgG+	2.71	42	44	43	56	15	3	53	38	38
anti-AQP4-IgG-	2.76	43	42	37	38	14	7	69	48	24

	functional scores				fatigue and depression scores			
	Multiple Sclerosis Functional Composite (MSFC)	The Short Form (36) Health Survey (SF-36)	visual analog scale (VAS). general	visual analog scale (VAS) cognition	fatigue severity scale (FSS)	Fatigue Scale for Motor and Cognitive Functions (FSMC)	Beck Depression Inventory (BDI)	visual analog scale (VAS) fatigue
anti-AQP4-IgG+ and anti-MOG-IgG+: double AQP4+MOG-IgG positive	9	97	3	69	15	27	5	50
anti-AQP4-IgG+ and anti-MOG-IgG-: single AQP4-IgG positive	9	98	41	32	35	38	9	41
anti-AQP4-IgG- and anti-MOG-IgG+: single MOG-IgG positive	8	99	43	25	32	34	10	41
anti-AQP4-IgG- and anti-MOG-IgG-: no autoantibody	8	101	46	38	35	39	14	50
anti-AQP4-IgG+	8	98	39	32	35	37	9	37
anti-AQP4-IgG-	8	99	46	32	35	37	12	47

	pain scores				visual function scores	
	McGill Pain Questionnaire (MPQ)	painDETECT questionnaire (PDQ)	Brief Pain Inventory (BPI)	visual analog scale (VAS) pain	National Eye Institute Visual Function Questionnaire (NEI-VFQ)	National Eye Institute Visual Function Questionnaire (NEI-VFQ) and Neuro-Ophthalmic Supplement (NOS)
anti-AQP4-IgG+ and anti-MOG-IgG+: double AQP4+MOG-IgG positive	44	42	54	51	111	51
anti-AQP4-IgG+ and anti-MOG-IgG-: single AQP4-IgG positive	28	28	32	33	112	46
anti-AQP4-IgG- and anti-MOG-IgG+: single MOG-IgG positive	22	21	30	25	113	44
anti-AQP4-IgG- and anti-MOG-IgG-: no autoantibody	38	30	39	35	115	47
anti-AQP4-IgG+	31	28	35	35	108	47
anti-AQP4-IgG-	29	26	36	32	114	45
